# Advances in Biologic Therapies for Allergic Diseases: Current Trends, Emerging Agents, and Future Perspectives

**DOI:** 10.3390/jcm14041079

**Published:** 2025-02-08

**Authors:** Ewa Alska, Dariusz Łaszczych, Katarzyna Napiórkowska-Baran, Bartłomiej Szymczak, Alicja Rajewska, Aleksandra Ewa Rubisz, Paulina Romaniuk, Katarzyna Wrzesień, Natalia Mućka, Zbigniew Bartuzi

**Affiliations:** 1 Department of Allergology, Clinical Immunology and Internal Diseases, Collegium Medicum Bydgoszcz, Nicolaus Copernicus University Torun, 85-067 Bydgoszcz, Poland; e.alska@icloud.com (E.A.); zbartuzi@cm.umk.pl (Z.B.); 2 Student Research Club of Clinical Immunology, Department of Allergology, Clinical Immunology and Internal Diseases, Collegium Medicum Bydgoszcz, Nicolaus Copernicus University Torun, 85-067 Bydgoszcz, Poland; laszczychdariusz@gmail.com (D.Ł.); bartlomiej.szymczak1@gmail.com (B.S.); alicja.p.rajewska@gmail.com (A.R.); aleksandra.rubisz98@gmail.com (A.E.R.); romaniuk_paulina@wp.pl (P.R.); krzyzakkatarzyna0@gmail.com (K.W.); mnata.99@gmail.com (N.M.)

**Keywords:** biologics, monoclonal antibodies, allergy, cytokines, asthma, atopic dermatitis, spontaneous urticaria, chronic rhinosinusitis, allergic rhinitis

## Abstract

Biologic therapies have revolutionized the treatment of severe allergic diseases, including asthma, atopic dermatitis (AD), chronic spontaneous urticaria (CSU), chronic rhinosinusitis with nasal polyps (CRSwNP), eosinophilic gastrointestinal diseases (EGIDs), and allergic rhinitis (AR). These molecularly targeted agents provide significant benefits for patients unresponsive to conventional treatments by addressing underlying immune mechanisms, particularly type 2 inflammation driven by cytokines such as IL-4, IL-5, and IL-13. Recent advancements include biologics targeting alarmins like thymic stromal lymphopoietin (TSLP) and IL-33, which may address both type 2 and non-type 2 inflammation, broadening their therapeutic scope. Despite their effectiveness, biologics remain expensive, posing socioeconomic challenges, and there are concerns regarding long-term safety and inter-individual variability in responses. Promising innovations such as bispecific antibodies and ultra-long-acting agents are under investigation, alongside digital health tools like remote biomarker monitoring and AI-driven decision support systems, which aim to enhance personalized care. However, disparities in access, particularly for underserved populations, underscore the need for policy reforms and affordable biosimilars. This review synthesizes recent findings and emerging trends, highlighting the evolving role of biologics in transforming allergic disease management and offering insights into future research directions.

## 1. Introduction

Allergic diseases, including asthma, atopic dermatitis (AD), chronic spontaneous urticaria (CSU), chronic rhinosinusitis with nasal polyps (CRSwNP), non-esophageal eosinophilic gastrointestinal disorders (non-EoE-EGIDs), and allergic rhinitis (AR), are a growing global health concern [1]. These conditions are characterized by immune system overreaction to environmental allergens, leading to inflammation, tissue damage, and chronic symptoms [2]. Allergic diseases represent one of the most common chronic diseases worldwide, significantly affecting individuals’ quality of life (QoL) and imposing a substantial economic burden on healthcare systems [3].

The prevalence of allergic diseases has risen significantly in industrialized countries, and the overall burden is increasing globally, particularly in urban populations. Asthma affects up to 10% of the global population, with higher rates in children and a marked increase in recent decades [4]. AD affects up to 4% of children (approximately 102.78 million patients) and 2% of adults (approximately 101.27 million individuals) in the global context with an unequal geographical distribution [5]. The prevalence of CRSwNP reaches up to 20% in some regions [6], with an incidence of 1–4% in developed countries such as the United States [7]. Chronic urticaria impacts around 0.5–1.5% of the population [8]. The prevalence of eosinophilic gastrointestinal diseases (EGIDs) varies depending on the anatomical location of the lesions, with eosinophilic esophagitis being the most common subtype and eosinophilic colitis the rarest [9]. Additionally, AR affects 10–30% of adults and more than 40% of children worldwide [10].

The economic burden of allergic diseases is considerable, with asthma alone accounting for billions of dollars in direct and indirect costs annually, including hospitalizations, medications, and lost productivity [11]. This growing prevalence, combined with the chronic nature of these diseases, underscores the urgent need for better treatment options and more effective therapies.

Traditional therapies for allergic diseases primarily focus on controlling symptoms rather than addressing the underlying immunologic causes. These treatments include antihistamines, corticosteroids (CS), leukotriene modifiers, and allergen immunotherapy. While these therapies can relieve symptoms, they are often insufficient for patients with severe or uncontrolled allergic conditions. In particular, severe asthma (i.e., asthma that remains symptomatic despite optimized and maximalized intensive treatment) affects up to 10% of asthmatic patients [12]. For AD, standard treatments like topical steroids often fail to control symptoms, especially in cases with significant flare-ups adequately. Moderate-to-severe AD, which is an indication for systemic therapy, accounts for nearly 40% of all AD cases in the United States [13]. Similarly, CSU (up to 50% of patients non-responding to standard antihistamine treatment) [14] and AR (approximately 40% with moderate-to-severe symptoms severity) [15] frequently do not respond to first-line therapies, highlighting the need for more effective and targeted interventions like biological therapies.

Biological therapies represent a paradigm shift in the treatment of allergic diseases by targeting specific immune components involved in the pathogenesis of these chronic conditions. Unlike traditional therapies, which broadly suppress inflammation, biologics are designed to interfere with specific cytokines, immune cells, and signaling pathways that drive allergic inflammation [16].

In asthma, biologics like omalizumab, an IgE monoclonal antibody (mAb), block the interaction between IgE and mast cells, preventing allergic reactions. Mepolizumab and reslizumab, which target IL-5, reduce the production and activation of eosinophils, a key driver of inflammation in eosinophilic asthma [17]. Dupilumab, which attenuates Th2-driven inflammation through inhibition of both IL-4 and IL-13 signaling, has demonstrated efficacy in a wide range of allergic conditions, including asthma and AD [18]. Tralokinumab, which also targets IL-13, has shown significant efficacy in reducing eczema severity and improving QoL [19]. In CSU, omalizumab has proven highly effective in symptom alleviation by preventing mast cell degranulation [19]. Currently, none of the biologics are approved for the treatment of Non-EoE-EGIDs. However, omalizumab is frequently used off-label with clinical efficiency [20]. To date, several biologics, including dupilumab, omalizumab, and mepolizumab, have shown efficiency and safety in the treatment of CRSwNP and have been approved by the U.S. Food and Drug Administration (FDA) [21].

Biologics have revolutionized the treatment of severe allergic diseases but pose significant socioeconomic challenges due to their high costs, representing up to 40% of medication expenditures while being a small fraction of prescriptions [22]. Although they reduce indirect costs like hospitalizations and productivity losses, their Incremental Cost-Effectiveness Ratio (ICER) often exceeds thresholds for cost-effectiveness [22]. Addressing these challenges requires policy reforms, biosimilar adoption, and innovations like at-home administration. While biologics show promise, their long-term safety remains uncertain. Real-world studies support tolerability, but risks like infections, autoimmune reactions, and malignancies require further investigation [23]. Response variability among patients is another challenge. Factors like genetics, biomarker profiles, and disease characteristics influence outcomes, with biomarkers like blood eosinophil count (BEC) and periostin linked to better responses but lacking universal applicability [24]. Advances in multi-omics and machine learning hold promise for personalized treatment but require broader validation [25].

Future directions in biologics focus on precision medicine, expanding therapeutic targets, and improving accessibility. Multi-target biologics, such as bispecific antibodies, offer the potential for complex endotypes of allergic diseases [2]. Biosimilars may address cost and accessibility issues while integrating real-world data and machine learning, which could enhance patient stratification and outcomes [25,26]. These advancements reflect the ongoing shift toward personalized, cost-effective, and comprehensive management of allergic diseases.

This review aims to provide a comprehensive overview of the current advancements in biological treatments for selected allergic diseases, including asthma, AD, CSU, non-EoE-EGIDs, CRSwNP, and AR. It highlights their mechanisms of action, clinical efficacy and safety, limitations, and emerging therapeutic approaches, with a particular focus on studies published between 2019 and 2024, offering a thorough and up-to-date synthesis of the latest findings.

## 2. The Biological Therapy in Selected Allergic Diseases: Current Standards and Future Perspectives

### 2.1. Asthma

#### 2.1.1. Epidemiology and Current Landscape

**Figure 1:**
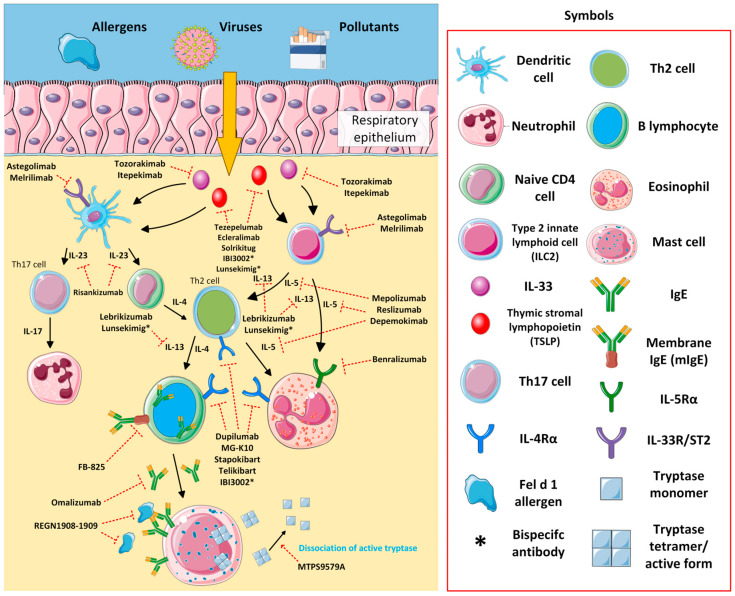
Cytokine network and biologics targeting inflammatory pathways in asthma. This Figure illustrates the role of key cytokines and immune cells involved in asthma pathogenesis, particularly the interplay between T2 and non-T2 inflammatory responses following exposure to allergens, viruses, and pollutants. Alarmins, such as thymic stromal lymphopoietin (TSLP) and IL-33, are released from the respiratory epithelium and initiate the inflammatory cascade by activating various immune cell types, including dendritic cells, Th17 cells, naive CD4 cells, and innate lymphoid cells (ILC2). These pathways drive cytokine production, such as IL-4, IL-5, IL-13, and IL-17, leading to airway inflammation, mucus production, and tissue remodeling. Biologics reviewed in this manuscript are highlighted in black text, with their molecular targets indicated by red dashed lines. Key biologics include those targeting TSLP (tezepelumab, ecleralimab, solrikitug), IL-33 (tozorakimab, itepekimab), IL-5 (mepolizumab, reslizumab, depemokimab), IL-4R (dupilumab, MG-K10, stapokibart, telikibart), IL-33R/ST2 (astegolimab, melrilimab) and IgE (omalizumab). Bispecific antibodies (IBI3002, lunsekimig) and emerging therapies, such as tryptase inhibitors and allergen-specific therapies, are also depicted. For detailed mechanisms and references, please refer to the relevant sections in the text. Symbols for immune cells and molecules are defined on the right panel for clarity.

Asthma is a heterogeneous, chronic disease characterized by persistent inflammation of the airways. It is associated with excessive mucus secretion in the airways, varying degrees of obstruction, and irreversible tissue remodeling, causing significant consequences that reduce QoL [27]. Asthma is estimated to affect more than 300 million people worldwide, with approximately 8–10% of patients developing into the severe type [12,28]. Approximately 250,000 people die prematurely every day due to asthma, so it is a major global health problem that reduces QoL but also contributes to increased healthcare costs [29]. Currently, the mainstay of asthma treatment is inhaled corticosteroids (ICS) and a long-acting β-receptor agonist. Unfortunately, despite the maintenance of appropriate asthma management procedures, there is a group of patients for whom standard treatment is unsuccessful and symptom control is ineffective [27]. The appearance of biological therapies in the treatment of asthma provides a promising therapeutic strategy, especially for patients with severe and difficult-to-treat asthma, whose disease is poorly controlled and whose standard treatment is unsuccessful [30]. Figure 1 illustrates the mechanisms of action of existing and emerging biologics in asthma.

#### 2.1.2. Asthma Phenotypes: T2 vs. Non-T2

Biologics revolutionized the management of severe, uncontrolled asthma. However, it is important to note that several factors influence the efficiency of biological treatment in patients with asthma. One of the key factors is the inflammatory phenotype of asthma: T2-high phenotype (which includes allergic and eosinophilic asthma) and non-T2/low-T2 phenotype [28]. The T2-high allergic phenotype is defined by the Th2 inflammatory response (elevated IgE levels, increased BEC of ≥150 μL, and elevated FeNO ≥ 20 ppb) [31]. Patients with the T2-high phenotype of asthma frequently struggle with increased symptoms, reduced lung function, frequent exacerbations, and more common resistance to high doses of conventional therapies. On the other side, patients with confirmed Th2 inflammation may benefit from biologics targeting these inflammatory pathways, including anti-IgE, anti-IL-5, and anti-IL4Rα agents [32]. The T2-low phenotype is often defined by an abundant granulocytic profile, with normal eosinophil levels, reduced FeNO levels, peripheral neutrophilia, and a poor response to glucocorticoids. This type of asthma is rarely associated with allergy and is suspected to be associated with obesity, chronic sinusitis, and nasal polyps [33]. Non-T2 asthma is mediated by several immune cells and cytokines. Due to the promotion of neutrophilic airway infiltration and mucus hypersecretion, the Th17 axis (involving Th17 cells, IL-23, IL-17, and IL-6) is considered a key pathway involved in non-T2 asthma [34]. In T2-low asthma, the role of biologics is less established compared to T2 asthma [33]. Long-acting muscarinic antagonists are often considered key components for non-T2 asthma treatment. However, several options may specifically target non-T2 inflammatory pathways. Among them, erythromycin, a macrolide antibiotic that reduces exacerbation rates in adults with severe asthma, and imatinib, due to its inhibitory effect on mast cells and promising results of phase 2 trial, are currently under investigation in non-T2 asthma [35]. Furthermore, several biologics are under investigation in non-T2 asthma [36].

#### 2.1.3. Biologic Therapies for T2-High Asthma

According to the GINA 2024 recommendations for the treatment of severe and difficult-to-treat allergic T2-high phenotype asthma, the current first-line treatment for biologics are anti-IgE omalizumab, anti-IL-5/anti-IL-5R benralizumab, mepolizumab, reslizumab, anti-IL4Rα dupilumab, and anti-thymic stromal lymphopoietin (TSLP) tezepelumab [37].

Omalizumab is the first mAb approved for asthma treatment, and it blocks the free serum IgE, which limits its binding to the receptor located on mast cells and basophils; ultimately, this leads to weakening the inflammatory response caused by resulting from a response to contact with an allergen [38]. The effect of omalizumab action also includes reducing the number of eosinophils, and this is why it shows high effectiveness in patients with asthma who have higher levels of eosinophils. IgE blocking lowers susceptibility to infections and, as a result, protects young patients with asthma from exacerbations [38]. Omalizumab is recommended in ≥6-year-old patients. The dosage of omalizumab is determined by the patient’s body weight and IgE serum, and it is served subcutaneously every 2 or 4 weeks [37].

Benralizumab, mepolizumab, and reslizumab are humanized mAb targeting the IL-5 pathway, inhibiting eosinophils’ differentiation and survival and migration from the bone marrow into the systemic circulation [28]. Benralizumab binds precisely to the α subunit of the IL-5 receptor. It is administered subcutaneously at a dose of 30 mg every 4 weeks (in 3 doses) and every 8 weeks in adults and children aged ≥12 years with BEC ≥ 150 cells/μL [39]. Mepolizumab is a recombinant humanized IgG1, which blocks the interaction between IL-5 and its receptor, preventing IL-5 signaling [40]. Mepolizumab has been approved in adults and children ≥6 years with BEC ≥ 150 cells/μL, and it is administered subcutaneously with 100 mg every 4 weeks [37]. Similarly, reslizumab binds circulating IL-5 and is indicated for use in adults as an intravenous infusion at a dose of 3 mg/kg every 4 weeks [37].

Dupilumab is a mAb targeting the α subunit of the IL-4 receptor (IL-4Rα), leading to blocking the action of the IL-4 and IL-13 [41]. These cytokines are involved in the recruitment of eosinophils in the airways, as well as cup cell hypertrophy and mucus production, remodeling the airways [41]. According to GINA severe asthma guidelines, dupilumab can be used in patients with severe eosinophilic asthma in patients aged ≥12 years old with exacerbations within the past year with a BEC ranging between 150–1500 cells/μL or FeNO > 25 ppb or requirement for maintenance OCS [37]. It is used as a subcutaneous injection of 200 mg or 300 mg every 2 weeks. In severe asthma dependent on OCS or concomitant moderate-to-severe atopic dermatitis (AD), a dose of 300 mg is indicated [37].

Tezepelumab is a humanized IgG2λ mAb that binds to thymic stromal lymphopoietin (TSLP), preventing it from interacting with its receptor [42]. TSLP, similarly to IL-33, is a bronchial epithelium-derived alarmin that induces the production of T2 and non-T2 cytokines responsible for asthma symptoms [42]. Tezepelumab is recommended by GINA for asthma treatment in patients aged ≥12 by subcutaneous injection at a dose of 210 mg every 4 weeks [37].

Although several biologics are currently approved for asthma therapy, numerous novel drugs are under investigation. Table 1 summarizes the findings from recent clinical trials of these emerging biologics in asthma. The detailed summary of the discussed studies is presented in Table S1.

Depemokimab is a biological drug characterized by an ultra-long duration of action that allows the drug to be effectively dosed at 6-month intervals [43]. The drug has an affinity for the interleukin 5 receptor (IL-5R), which is responsible for the growth, differentiation, activation, and survival of eosinophils and affects the activity of inflammatory cells [44]. In 2022, the results of phase 1, a double-blind trial evaluating the safety, tolerability, pharmacokinetics, and pharmacodynamics of depemokimab, were published. Adverse events incidence was lower in the depemokimab-treated group (81%) than in the placebo-treated group (92%), with no adverse events leading to the treatment discontinuation. A single depemokimab dose was associated with a >48% reduction in BEC after 24 h. The study demonstrated an extended half-life of depemokimab and less frequent dosing compared to available anti-IL-5/IL-5R drugs like benralizumab, mepolizumab, and reslizumab, which may increase adherence to the therapy and decrease the therapy-related costs [45]. Recently, the results of two multicentre, randomized phase 3a trials SWIFT-1 and SWIFT-2 confirmed a reduction in exacerbation rates in patients with severe eosinophilic asthma treated with depemokimab compared to the placebo group. Depemokimab has been shown to dose-dependently inhibit BEC for approximately 26 weeks rapidly and permanently, and administration of depemokimab at 6-month intervals for 52 weeks was associated with a significant reduction in annualized asthma exacerbation rate (AAER) [43]. Studies on depemokimab to date build upon the existing evidence for biologic therapies targeting IL-5 or its receptor (e.g., mepolizumab, reslizumab, and benralizumab), further emphasizing their efficacy in treating eosinophilic asthma [44]. Head-to-head trials are necessary to compare the efficacy of depemokimab with existing anti-IL-5 and anti-IL-5R therapies.

Lebrikizumab is an IgG4 mAb directed against interleukin 13 (IL-13), which in the field of allergic diseases is mainly used for the treatment of moderate to severe uncontrolled asthma in adults and adolescents [46]. IL-13 is secreted by type-2 helper T cells (Th2), mast cells, and basophils. This cytokine is involved in IgE production, promotion of eosinophil migration, maturation of airway goblet cells, and airway smooth muscle hyperplasia, proliferation, and contractility. Given the significant role of IL-13 in asthma pathophysiology, its inhibition with mAb is a promising therapeutic avenue in the diseases [47]. While initial results from clinical trials of lebrikizumab appear promising, a considerable proportion of studies have not demonstrated a consistent reduction in the rate of asthma exacerbations. The randomized, placebo-controlled phase 2 CLAVIER study, which assessed the effect of lebrikizumab on airway eosinophilic inflammation and remodeling in patients with uncontrolled asthma, did not demonstrate a reduction in subepithelial eosinophil count in response to the treatment [48]. Nevertheless, pre-specified exploratory analyses revealed that lebrikizumab treatment reduces airway fibrosis, confirming the importance of the IL-13 pathway in reducing airway remodeling [48]. In a notable development, the results of three other phase 3 trials, LAVOLTA I, LAVOLTA II, and ACOUSTICS, have been published [49,50]. However, their results did not show a consistent reduction in asthma exacerbations. Despite the promising results observed in phase 2 clinical trial, where lebrikizumab demonstrated a significant reduction in exacerbations and improvement in FEV1 in patients with uncontrolled asthma, these findings were not replicated in phase 3 trials, where a reduction in asthma exacerbations was not observed in patients despite the evident ability to inhibit IL-13 [49]. Recently, the results of a post hoc reanalysis of phase 3 trials have been published, demonstrating that lebrikizumab at 125 mg and 37.5 mg significantly reduced AAER compared with placebo in patients with a BEC of ≥300 cells/mL and ≥1 asthma exacerbations in the preceding year [51]. Lebrikizumab appears to be a preferred option for patients with T2 asthma who experience disease exacerbations. However, as one of the few anti-IL-13 therapies available for asthma, there is an urgent need to assess whether lebrikizumab may be preferred over existing biologics and under what circumstances, considering potential biomarkers and clinical factors.

Tozorakimab (MEDI3506) is a human mAb with a dual mechanism of action directed against IL-33. It is a relatively new therapeutic agent that inhibits IL-33red and IL-33ox signaling, indirectly reducing inflammation and airway epithelial dysfunction. The efficacy and safety of this drug are also being investigated in asthma [52]. IL-33 is a constitutively expressed alarmin, a type of cytokine released in response to tissue injury caused by allergens, airway pollutants, or viral and bacterial exposure. In asthma, IL-33 is upregulated in airway epithelial cells and serves as a biomarker for disease severity. Upon binding to its receptor (ST2/IL1RL1/IL-33R), which is expressed on various immune cells, including neutrophils, eosinophils, basophils, and mast cells, IL-33 promotes both T2 and non-T2 inflammation. It facilitates eosinophil migration, mucus hypersecretion, secretion of IL-4, IL-5, and IL-13, IgE production by B cells, and airway hyperresponsiveness. Moreover, IL-33 directly activates mast cells, in addition to its indirect effects mediated through IgE. Furthermore, IL-33 also activates neutrophils, contributing to non-T2 airway inflammation [53]. The results of the phase 2a trial FRONTIER-3, which evaluated the efficacy and safety of tozorakimab in moderate-to-severe early-onset asthma (diagnosed <25 years of age), have shown that tozorakimab did not meet his primary endpoint, i.e., the change from baseline to week 16 in pre-treatment FEV1 compared to placebo [54]. However, tozorakimab improved FEV1 in patients with a history of ≥2 exacerbations in the previous 12 months. Furthermore, tozorakimab treatment decreased rescue medication use compared to the placebo group. Notable, tozorakimab treatment was associated with a significant decrease in type 2 inflammatory biomarkers, including FeNO and BEC [54].

Itepekimab is a relatively new mAb targeting IL-33 [55]. It has been proven that this specific medication can diminish the inflammatory response within the airways, which results in a reduction of tissue damage and a decrease in BEC [56]. A recent phase 2 trial study showed that treating with itepekimab improved the patients’ QoL and the control of asthma exacerbations (NCT03387852) [55].

Another mAb that suppresses IL-33 signaling is melrilimab (GSK3772847/CNTO-7160). It inhibits the IL-33 pathway by binding to the extracellular domain of the ST2/IL-33R, thereby reducing the activation of immune cells and secretion of pro-inflammatory cytokines [57,58]. Phase 1 studies have demonstrated preliminary safety and biological activity, resulting in sustained inhibition of p38 basophil phosphorylation and suppression of free sIL-33R/soluble suppressor of tumorigenicity 2 (sST2), which leads to overall IL-33R/ST2 signal downregulation and ultimately decrease in airway inflammation. However, no effect on the clinical picture of the subjects was observed. Notably, at least one treatment-emergent adverse event (TEAE) occurred in 82.4% of subjects who received a single dose of melrilimab compared to 64.7% in the placebo group. All TAEAs were mild to moderate. The incidence of TAEAs was not related to the dose of melrilimab [58]. The phase 2a study (NCT03393806), which evaluated the efficacy of melrilimab in patients with moderate-to-severe asthma accompanied by allergic fungal airway disease, confirmed alterations in ST2 levels. Specifically, the study observed a reduction in free sST2 and an increase in total sST2 in the melrilimab-treated group, thereby validating the target engagement of this novel agent. Nevertheless, no discernible impact on reducing BEC and FeNO levels was observed, a finding that contrasts with studies on alternative mAb targeting IL-33 axis like itepekimab and astegolimab [55,57]. An additional phase 2a study that also evaluated the safety and efficacy of melrilimab in the treatment of asthma observed a reduction in the proportion of participants with loss of asthma control (LoAC) and prolonged median time to LoAC compared to the control group. In addition, lower levels of free sIL-33R and eosinophils in the circulation were observed [59]. The incidence of AE was slightly higher in the placebo arm than in the melrilimab arm (45% vs. 39%). Treatment-related AE was higher in the melrilimab group (10% vs. 4%) [59]. Further research on melrilimab, particularly in comparison with other biologics targeting IL-33 signaling, is warranted, given its potential as a treatment for asthma.

Astegolimab (MSTT1041A) is a fully human IgG2 mAb targeting ST2/IL-33R, thereby inhibiting IL-33 signaling, a key alarmin involved in asthma pathogenesis [60]. The phase 2b ZENYATTA trial, conducted in adults with severe asthma, demonstrated that 52-week treatment with astegolimab significantly reduced AAER, with the most pronounced reduction observed in the 490 mg regimen (43% decrease) [61]. Notable, AAER reduction was even higher in the eosinophil-low subgroup (<300 cells/mL) with a 54% reduction over placebo. On the other hand, the eosinophil-high subgroup did not achieve significant improvement in AER compared to placebo. Astegolimab prolonged time to the first asthma exacerbation compared to placebo, but there was no significant improvement in prebronchodilatator FEV1 at week 54 compared to placebo. Astegolimab was well-tolerated, and the incidence of adverse events was similar in astegolimab- and placebo-treated groups [61].

Risankizumab is a mAb against interleukin 23 (IL-23), which has previously shown efficacy in psoriasis and Crohn’s disease [62]. It has been noted that IL-23 levels are high in patients with asthma and are associated with worsened lung function. The IL-23 pathway, directly through the promotion of Th17 cells and the subsequent release of IL-17, stimulates the proliferation of fibroblasts and airway smooth muscle, thereby contributing to airway remodeling [63]. Notable, independent of the Th17 axis, IL-23 promotes T2 inflammation and eosinophil infiltration [64]. Despite this, the published phase 2a trial (NCT02443298) did not demonstrate a beneficial effect of risankizumab on the frequency of exacerbations in severe asthma [65]. The study did not meet his primary endpoint, i.e., the time to the first asthma worsening. Notable, the time to the first asthma worsening was significantly shorter in risankizumab than in placebo (median, 40 days vs. 86 days). AAER was higher in the risankizumab group. Sputum transcriptomic analysis showed that the risankizumab-treated group was characterized by the downregulation of genes involved in activating natural killer cells and cytotoxic T cells. Risankizumab treatment was well-tolerated, with a similar incidence of adverse events and serious adverse events in the two groups [65]. These results, therefore, call into question the role of the IL-23 pathway in the pathogenesis of asthma.

Ecleralimab (CSJ117) is the first inhaled anti-TSLP antibody fragment. Ecleralimab represents a promising alternative drug-delivering method compared to subcutaneous or intravenous injections [66]. Ecleralamib is a pharmacological powder covered by hard capsules that is delivered into airways with a dry-powder inhaler. Since asthma is associated with inflammation within the bronchial mucosa, this innovative administration route may increase the efficiency of treatment while limiting systemic treatment-related adverse events. In addition, inhaled administration enhances patient adherence and facilitates home-based therapy. However, improper inhalation techniques can significantly compromise treatment efficacy; therefore, patient education is crucial [66]. The results of the phase 1 randomized, double-blind, placebo-controlled bronchoprovocation trial, which aimed to evaluate the safety, tolerability, pharmacokinetics, and pharmacodynamics of ecleralimab in adults with mild atopic asthma, were recently published. The primary end-points were allergen-induced change in FEV1 during the late asthmatic response (LAR), maximum percentage decrease of LAR (LAR%) on day 84, and the safety of ecleralimab. Ecleralimab significantly decreased LAR, LAR%, and allergen-induced sputum eosinophil count. A significant decrease from baseline FeNO was observed. The majority of adverse events were mild in severity; the incidence and severity of TEAEs were similar between ecleralimab-treated and placebo groups [67]. Later, two phase 2 trials were conducted to evaluate the efficacy and safety of ecleralimab in severe uncontrolled asthma (NCT04410523, NCT04946318). Unfortunately, both trials were terminated prematurely due to sponsor decisions with no official publication in medical journals to date.

REGN1908 and REGN1909 are fully human IgG4 mAb that independently and non-competitively bind to the cat allergen Fel d 1, thereby preventing the binding of IgE to the antigen and consequently reducing the IgE-dependent allergic reaction [68]. The administration of a 1:1 mixture of monoclonal allergen-specific mAb represents a novel approach to passive immunization strategies for treating cat allergy [69]. The efficacy of this therapy, in terms of its ability to reduce ocular cat allergy signs and symptoms in patients, is currently under evaluation in phase 3 clinical trials (NCT06602726). The results of the first and second phase clinical trials reported promising results in terms of tolerability, safety, and efficacy in the treatment of cat allergy-induced rhinitis in patients with mild asthma. The phase 1, first-in-human study (NCT01922661) showed that REGN1908-1909 has a favorable tolerability and safety profile, with a single dose of 600 mg maintaining mean serum levels of total mAb above target (average ~10 mg/L) for 8–12 weeks. TAEA occurred in 55.6% (*n* = 10) of the REGN1908-1909 arm compared to 66.7% (*n* = 6) in the placebo arm. None of the TEAEs was serious, and all were mild-to-moderate in severity. No case of TAEAs leading to the treatment discontinuation was reported [68]. A year later, the findings of a phase 2 randomized, double-blind study (NCT03838731) evaluating the efficacy of REGN1908/1909 in preventing cat allergen-induced early asthmatic responses (EARs) were published [69]. The results demonstrated that a 600 mg dose of the antibody mixture (1:1 ratio, 300 mg each) effectively protected against allergen-induced FEV1 decline for up to 85 days post-dosing. This treatment not only prevented early asthmatic responses (EARs) but also significantly increased allergen tolerance in cat-allergic patients, with a threefold improvement in tolerance to higher allergen levels and a marked reduction in skin reactivity to cat allergen [69].

MTPS9579A is a novel, humanized IgG4 mAb targeting tryptase activity through irreversibly dissociating active tetramers into nonactive monomers [70]. Tryptase is a serine protease stored in secretory granules of human mast cells. Tryptase exacerbates the inflammatory process by promoting the recruitment of eosinophils and mononuclear cells and degrading the extracellular matrix. It plays a pivotal role in the pathophysiology of conditions associated with mast cell degranulation, such as asthma and anaphylaxis. Therefore, targeting tryptase activity may be a novel therapeutic approach in allergic diseases [71]. In 2021, the first-in-human phase 1 study in healthy individuals, MTPS9579A, appeared with a good safety and tolerability profile together with a rapid and dose-dependent reduction in the upper airway tryptase activity. Sixty-three participants who received MTPS9579A (76.8%) reported a total of 339 TEAEs compared to 19 participants (79.2%) who received a placebo and experienced 74 TEAEs. There were no reports of deaths or serious or life-threatening AEs. The majority of TEAEs were mild in severity and not related to MTPS9579A [72]. Later, Rymut et al., using a mechanistic pharmacokinetic/pharmacodynamic model, showed that MTPS9579A in a dose of 900 mg or greater administered IV once every 4 weeks may completely neutralize active tryptase in the upper airways of asthmatic patients [73]. Recently, the results of the phase 2a trial, including individuals with a history of ≥ 2 asthma exacerbations within the last 12 months and therapy consisting of daily ICS and at least one additional controller therapy, have been published. The primary endpoint was time to the first composite exacerbation event [74]. Unfortunately, MTPS9579A did not meet the primary endpoint, i.e., no significant difference in time to the first composite asthma exacerbation between MTPS9579A and the placebo group was demonstrated. In addition, the lower airway/bronchial concentration of MTPS9579A was 6.8-fold lower, and bronchial tryptase levels were 119-fold higher compared to upper airway/nasal levels. The proportions of patients experiencing AEs, serious AEs (6.2% in placebo vs. 7.2% in MTPS9579A arm), or death were comparable between MTPS9579A and placebo groups. There was one case in each arm of COVID-19 pneumonia-related deaths, which were not related to the treatment of the study [74]. In conclusion, this study demonstrated that the MTPS9579A dose predicted in earlier studies was insufficient to inhibit tryptase activity in the lower airway, which was associated with a lack of clinical benefit in asthma patients. However, due to their specific mechanisms of action, anti-tryptase therapies hold significant promise for asthma and other allergic diseases, warranting further investigation.

#### 2.1.4. Biologic Therapies for Non-T2 Asthma

According to the GINA 2024 recommendations for severe and difficult-to-treat asthma, biologic therapies such as anti-IL4Rα dupilumab and anti-TSLP tezepelumab should be considered in patients with non-T2 phenotype asthma, especially those requiring oral CS for maintenance [37]. In a post hoc analysis of the LIBERTY ASTHMA QUEST trial, it has been demonstrated that 200 mg dupilumab every 2 weeks (Q2W) may be more beneficial in asthmatic patients who did not meet the criteria for allergic asthma, i.e., IgE ≥ 30 IU/mL and ≥1 positive perennial aeroallergen–specific IgE value (≥0.35 kU/L) at baseline. In this subgroup, dupilumab significantly decreased AAER by up to 60% compared to placebo, while in the subgroup who met allergic asthma criteria, AAER was decreased only by 37% compared to placebo. Similarly, dupilumab 200 mg Q2W significantly improved prebronchodilator FEV1 by 0.14 L in the non-allergic asthma subgroup and by 0.13 L in the allergic asthma subgroup compared to placebo. In addition, asthma control at week 24 was slightly higher in the non-allergic asthma subgroup compared to the allergic asthma subgroup [75]. In the phase 2 CASCADE trial, tezepelumab was associated with significantly reduced airway hyperresponsiveness independent of baseline BEC, although no differences in submucosal neutrophil counts were observed [76]. In the phase 2 PATHWAY trial, tezepelumab in a dose of 210 mg Q4W reduced AAER by 84% (compared to placebo) in patients with low T2 status, i.e., IgE ≤ 100 IU/mL or eosinophil count < 140 cells/µL [77]. In NAVIGATOR, a phase 3 study, tezepelumab in a dose of 210 mg Q4W reduced AAER by 39% in patients with BEC < 150 cells/µL and by 61% in patients with BEC ≥ 150 cells/µL compared to placebo. Furthermore, AAER decrease was 32% in the subgroup with baseline FeNO < 25 ppb versus placebo [78]. A pooled analysis of PATHWAY and NAVIGATOR trials showed that tezepelumab is effective in the double and triple T2-low biomarker subgroups (defined as BEC < 150 cells/µL, FeNO < 25 ppb, and absence of perennial aeroallergen sensitization) with an AAER decrease of 34–45% compared to placebo. In the BEC < 150 cells/µL subgroup, tezepelumab was also associated with a 60% decrease in exacerbations requiring hospitalizations or emergency department visits compared to placebo (in the ≥150 cells/µL subgroup, this decrease was 86%) [79]. Evidence from clinical trials highlights tezepelumab and dupilumab as effective add-on therapies for severe and difficult-to-treat non-T2 phenotype asthma. Importantly, future trials should focus exclusively on non-T2 asthma populations, as current findings are largely derived from subgroup and exploratory analyses.

To date, the efficiency and safety of several biologics in non-T2 asthma have been evaluated. However, most of them, including anti-IL17 agents such as secukinumab and brodalumab and anti-TNF-α golimumab, have shown no clinical benefits [80]. On the other side, some of them exhibited promising effects and emerged as potential novel non-T2 therapeutics. In a double-blind, placebo-controlled study involving 17 healthy individuals, anakinra (anti-IL1R agent) significantly reduced airway neutrophil counts compared with placebo. In addition, anakinra treatment was associated with a significant reduction of IL-1β, IL-6, and IL-8 levels in sputum [81]. In the phase 2b trial, it has been demonstrated that astegolimab (anti-IL33R) in patients with low eosinophil counts (<300 cells/μL) was associated with a significant reduction in asthma exacerbation rate (AER) compared to placebo (54% and 35% for 490 mg and 70 mg doses, respectively). Notably, in the high eosinophil subgroup, none of the tested astegolimab doses showed significant improvement in AER compared to placebo. In addition, patients with blood eosinophil counts <150 cells/μL had higher FEV1 improvement compared to patients with >150 cells/μL eosinophils [61]. Recently, the anti-IL-23 mAb risankizumab was evaluated in severe persistent asthma. Unfortunately, risankizumab did not improve asthma control and was inferior to placebo. Subgroup analyses showed that the hazard ratio (HR) for the time to first asthma worsening in risankizumab-treated patients was higher in the high BEC subgroup (≥200 cells/mm^3^, HR 1.76) compared to the lower BEC subgroup (<200 cells/mm^3^, HR 1.10). Furthermore, risankizumab reduced sputum neutrophil counts. Transcriptomic analysis of sputum revealed that risankizumab downregulated the expression of the Retinoic acid-related orphan receptor gamma t (RORC) gene, a transcription factor involved in Th17 cell differentiation. Additionally, risankizumab downregulated the expression of genes involved in activating pro-inflammatory cytotoxic T cells and natural killer cells [65]. Despite the lack of clinical efficacy of risankizumab in severe asthma, the observed anti-inflammatory activity and the pivotal role of the Th17 axis in non-T2 asthma pathophysiology suggest that targeting this pathway with anti-IL-23 or anti-IL-17 agents represent a promising therapeutic avenue for non-T2/low-T2 asthma.

Biologics have shown potential as add-on therapies for severe non-T2 asthma, but current evidence remains limited and primarily derived from subgroup analyses. Clinical decisions in these specific asthma subgroups should align with current GINA 2024 guidelines; however, due to the paucity of data, physicians may need to rely on their clinical experience with particular biologics. Future research should focus on conducting dedicated trials for non-T2 asthma and advancing novel therapeutic strategies to meet unmet clinical needs.

#### 2.1.5. Emerging Biologics in Asthma

Currently, several novel drugs are being tested for efficacy and safety in the treatment of asthma, targeting both T2 and non-T2 inflammatory pathways (Table 2). A detailed summary of currently ongoing trials in asthma is present in Table S1.

MG-K10 is a humanized mAb whose target is IL-4Rα. Currently, there is an ongoing phase 1b/2 interventional, placebo-controlled clinical trial (NCT05382910) on safety, pharmacokinetics, and preliminary efficacy in adults with moderate to severe asthma. As of now, no results from this trial have been published. CM310, also known as stapokibart, is a recombinant humanized mAb that binds to IL-4Rα and suppresses IL-4 and IL-13 signaling, thereby inhibiting the inflammatory response mediated by Th2 cytokines. The potential efficacy of stapokibart has already been studied in AR and AD [82]. A multicenter phase 2/3 study is being conducted (NCT05761028) to evaluate the safety, characteristics, and immunogenicity of CM310 in patients with moderate to severe asthma. Completion of the study is scheduled for 05-2032. Investigational FB-825 is a humanized mAb that targets the CεmX domain of membrane IgE (mIgE), resulting in the downregulation of mIgE-positive B cells and the production of IgE [83]. A multi-center trial is underway (NCT05008965) testing the efficacy and safety of FB825 in adult patients with moderate to severe allergic asthma. Solrikitug (MK-8226) is a next-generation anti-TSLP mAb with potentially higher clinical efficiency and improved safety profile compared to tezepelumab [84]. Currently, phase 2a randomized, double-blind, placebo-controlled trial is recruiting to evaluate the safety, tolerability, pharmacokinetics, immunogenicity, and pharmacodynamics in adults with asthma (NCT06496607). The efficacy and safety of depemokimab in severe eosinophilic asthma are being evaluated in ongoing, phase 3 clinical studies (NCT04718389, NCT05243680) in order to determine whether depemokimab compares in efficacy to already established anti-IL-5 biologics in asthma treatment, benralizumab, and mepolizumab (NCT04718389; active comparator trial) or the placebo (NCT05243680), as a follow-up of already completed SWIFT-1 (NCT04719832) and SWIFT-2 (NCT04718103) studies. IBI 3002 is a humanized bispecific mAb targeting both cell surface IL-4Rα and the alarmin TSLP, giving it superiority over other commercially available mAb with a single molecular target [85]. Currently, a first-in-human, single-ascending dose phase 1 study is ongoing to evaluate its safety, tolerability, pharmacokinetics, and pharmacodynamics in healthy individuals and mild-to-moderate asthma (NCT06213844). Lunsekimig is a novel bispecific molecule designed to inhibit both TSLP and IL-13, two key mediators involved in the pathophysiology of asthma. Recently, the results of the first-in-human, phase 1 trial assessing the safety, tolerability, pharmacokinetics, pharmacodynamics, and immunogenicity of lunsekimig in healthy individuals have been published [86]. Lunsekimig was well-tolerated with common TEAEs, including COVID-19 infection, nasopharyngitis, injection site reactions (mild severity), and headache. Lunsekimig exhibited low immunogenicity: 11.1% of individuals in a single ascending dose subgroup developed antidrug antibodies (ADA), while in multiple ascending dose subgroups, ADA was present in 43.8% of subjects [86]. Currently, there are three recruiting phase 2 trials investigating the efficacy and safety of lunsekimig in adults with mild-to-moderate high-risk asthma (NCT06676319) and moderate-to-severe asthma (NCT06102005, NCT06609239).

#### 2.1.6. Role of Biomarkers in the Biological Treatment of Asthma

Biomarkers play an important role in asthma phenotyping, which is crucial for selecting appropriate biological treatments and monitoring responses to therapy [87]. Currently, the biomarkers primarily assessed are those related to the T2-high phenotype of asthma. The most widely used biomarkers include BEC, FeNO, and sputum eosinophils [37]. Alongside T2 biomarkers, clinical data on comorbidities, body weight, and evidence of allergen sensitization (via skin prick test or specific IgE) also influence the choice of biologics. According to the recent GINA guidelines, omalizumab is preferred in patients with a history of sensitization to inhaled allergens and conditions such as CRSwNP and CSU. Predictors of a good response to omalizumab include BEC ≥ 260/μL, FeNO ≥ 19.5 ppb, and childhood-onset asthma [37]. On the other hand, anti-IL-5 therapies such as mepolizumab and reslizumab, or anti-IL5Rα therapies like benralizumab, are preferred in patients with BEC ≥ 150 or 300/μL or comorbidities such as CRSwNP and eosinophilic granulomatosis. Predictors of a good response to anti-IL-5 or anti-IL5Rα therapies include higher BEC, adult-onset asthma, a history of nasal polyps, and FEV1 < 65%. Similarly, anti-IL4Rα therapy with dupilumab is indicated for individuals with BEC ranging from 150 to 1500/μL or FeNO ≥ 25 ppb, those requiring maintenance OCS, or patients with comorbidities such as moderate-to-severe AD, CRSwNP, and eosinophilic esophagitis. Predictive factors for a good response to dupilumab include higher BEC and FeNO levels [37]. Lastly, anti-TSLP therapy with tezepelumab is not based on any of the currently available T2 biomarkers; however, it is preferred in patients with a history of severe exacerbations in the past year. Notably, as one of the few available options for non-T2 asthma, tezepelumab should be considered for patients with no evidence of type 2 inflammation [37]. A pooled analysis of phase 2b and three trials of tezepelumab suggests it is more beneficial for patients with T2-high asthma, as indicated by a greater reduction in AAER among T2-high biomarker subgroups compared to T2-low subgroups [79]. Predictors of a good response to tezepelumab include higher BEC and FeNO levels [37]. 

The development of novel biologic therapies for asthma, such as anti-IL-13, anti-IL-33, and anti-IL-23 agents, necessitates the identification of reliable biomarkers to guide treatment decisions. The novel agents targeting upstream pathways or non-T2 mechanisms could benefit from biomarker identification to refine patient selection and optimize outcomes.

For anti-IL-13 therapy, periostin has been proposed as a potential predictive biomarker of good response. Periostin, a cellular matrix protein involved in airway remodeling and subepithelial fibrosis, is secreted in response to IL-13 [88]. However, its role as a biomarker for anti-IL-13 biologics remains uncertain. In Phase 3 LAVOLTA studies investigating lebrikizumab, periostin failed to predict asthma exacerbations reliably [49]. Similarly, in the STRATOS studies investigating tralokinumab (an anti-IL-13 agent) for severe, uncontrolled asthma, periostin failed to predict treatment response. A lack of predictive value was also demonstrated for another biomarker associated with the IL-13 axis, dipeptidyl peptidase 4 (DPP-4) [89]. Some authors suggest that methacholine/mannitol hyperresponsiveness or CT mucus evaluation may serve as potential biomarkers for anti-IL-13 therapies in asthma, but further studies are needed to establish their predictive value [90].

Similarly, anti-IL-33 therapy also lacks specific predictive biomarkers in asthma [55]. However, results from recent trials of astegolimab indicate that BEC may be a potential predictor of treatment response. Patients with low BEC (<300 cells/μL) experienced a significant reduction in AER, while those with high BEC (≥300 cells/μL) showed no significant difference compared to placebo [61]. Evaluating IL-33 or soluble IL-33R concentrations in sputum or serum could potentially aid in selecting appropriate biologics. Additionally, IL-33 levels may serve as a prognostic biomarker, as higher serum IL-33 levels have been linked to more severe disease courses [91]. Future clinical analyses should address the role of IL-33 or IL-33R as predictive biomarkers for anti-IL-33 therapy.

To date, anti-IL-23 therapy has shown no clinical efficacy in asthma. However, transcriptomic data from the risankizumab trial suggest that individuals with non-T2 asthma may benefit more from biologics targeting the IL-23 axis [65]. Machine learning methods leveraging clinical data hold great promise for developing drug-specific prediction models, including those for anti-IL-23 agents. For example, Morikubo et al. recently presented an ustekinumab-specific treatment response prediction model for ulcerative colitis, achieving a positive predictive value of 68.8% and a negative predictive value of 71.4% [92]. Future research should focus on evaluating treatment response prediction models that incorporate multiple clinical features to optimize patient selection for anti-IL-23 therapies in asthma.

### 2.2. Atopic Dermatitis

#### 2.2.1. Epidemiology, Clinics, and Current Standard of Care

AD, otherwise known as eczema, is a chronic disease associated with the development of inflammatory dermatosis when an allergenic agent comes into contact with the skin. It affects about 20% of pediatric patients [93] and about 7% of adult patients in the United States [94]. Symptoms that occur during an allergic reaction include erythema, pruritus, and the appearance of papules, blisters, or oozing wounds. The Eczema Area and Severity Index (EASI) is a tool widely used to assess the severity of AD. It evaluates the extent and severity of skin lesions by considering factors such as erythema, skin thickness, dryness, cracking, and pruritus. The EASI score is calculated by evaluating four areas of the body (face/neck, upper body, lower body, and extremities) and assigning appropriate scores according to symptom severity [95].

According to the recommendations of the American Academy of Dermatology (AAD) 2024, the primary form of treatment for AD is the use of emollients and prescription topical therapies. When the symptoms of the disease are more severe, when AD occupies a large area of the skin, or when the symptoms of the disease are associated with a significant reduction in QoL, phototherapy or systemic therapy is recommended. Systemic therapies may include immunosuppression, corticosteroids, antimetabolites, Janus kinase (JAK) inhibitors, and biologics [96].

In the case of moderate-to-severe AD, the AAD recommends using dupilumab if a patient decides to initiate biologic therapy. Dupilumab is a mAb targeting IL-4Rα, administered at a standard dosage of 600 mg subcutaneously at initiation, followed by 300 mg every two weeks [96]. According to the FDA, dupilumab is indicated in patients aged 6 months and older [97]. 

The second biological drug included in the recommendation is tralokinumab, a mAb targeting IL-13. The standard dosing of tralokinumab is 600 mg at initiation, followed by 300 mg every two weeks. It is possible to reduce the dose to 300 mg every four weeks after 16 weeks of treatment if an adequate response is achieved [96]. Tralokinumab may be used in patients aged 12 years and older. Notable, tralokinumab may be combined with topical steroids [98]. 

According to the recommendations, both drugs have high-certainty evidence supporting their efficacy, appear to be safe, and do not require laboratory monitoring. A common adverse event observed with both drugs is conjunctivitis, which is generally self-limiting and often requires only the use of artificial tears [96].

#### 2.2.2. Emerging Biologic Agents for Atopic Dermatitis

**Figure 2:**
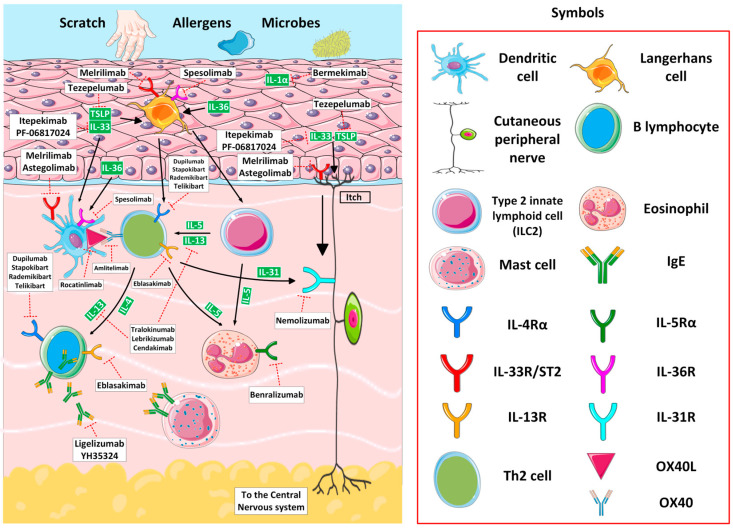
Cytokine network and biologics targeting inflammatory pathways in atopic dermatitis (AD). This Figure illustrates the key cytokines, immune cells, and therapeutic targets involved in the pathogenesis of AD, highlighting the role of allergens, microbes, and scratching in triggering skin inflammation. Stressful stimuli activate epithelial-derived alarmins, such as thymic stromal lymphopoietin (TSLP), IL-33, and IL-36, which initiate downstream signaling pathways involving immune cells like dendritic cells, Langerhans cells, mast cells, type 2 innate lymphoid cells (ILC2), and Th2 lymphocytes. These pathways drive the release of cytokines, including IL-4, IL-5, IL-13, and IL-31, contributing to skin inflammation, eosinophil recruitment, and pruritus. Biologics and targeted therapies reviewed in the context of AD are labeled in green boxes with white text, with their molecular targets indicated by red dashed lines. Notable agents include anti-IL-4Rα therapies (dupilumab, stapokibart, rademikibart, telikibart), anti-IL-13 signaling therapies (cendakimab, lebrikizumab, tralokinumab, eblasakimab), anti-IL-5R therapies (benralizumab), and anti-IL-31 therapies (nemolizumab). Agents targeting alarmins, such as TSLP (tezepelumab) and IL-33 signaling (itepekimab, melrilimab, PF-06817024), as well as novel anti-IL-36 therapies like spesolimab, are also shown. Additionally, therapies directed at IgE (e.g., ligelizumab, YH35324) and OX40/OX40L pathways (e.g., rocatinlimab, amitlelimab) highlight innovative strategies in AD management. For further details on mechanisms of action and clinical evidence, please refer to the relevant sections of the text. Symbols for immune cells and cytokine receptors are defined in the legend on the right for clarity.

While approved biologics are a milestone in the treatment of AD, some patients do not respond clinically to these agents. In the last few years, several novel agents targeting cytokines, their receptors, and signaling pathways have emerged (Figure 2). This paragraph covers emerging biologics in development for the treatment of AD. The results of recently published trials on novel biologics for AD are presented in Table 1, while ongoing trials are summarized in Table 2. The detailed versions are available in Table S1, and Table S2, respectively.

Bermekimab is a human-derived recombinant mAb that blocks interleukin-1α (IL-1α) activity. Four phase 2 studies evaluated the drug’s efficacy and safety. Bermekimab was administered to patients with moderate-to-severe AD at doses ranging from 200 mg to 800 mg per week. Despite the drug’s potential in preclinical studies, larger controlled trials failed to demonstrate its efficiency [99]. According to the safety profile reported during the GENESIS trial, a randomized, placebo- and active-comparator controlled study, 66% of patients in the bermekimab-treated group, 54.5% in the placebo group, and 50.8% in dupilumab-treated group experienced at least 1 AEs, respectively. While no serious AEs were reported in the placebo and dupilumab arm, four serious AEs were reported in the bermekimab arm. However, none of the serious AEs were related to the study intervention [99].

Lebrikizumab is a drug that selectively binds to IL-13. The drug has been tested in two clinical studies, ADvocate1 and ADvocate2 [100]. The dose of the mAb was 250 mg every two weeks for 16 weeks. After this period, patients were re-randomized to 250 mg of lebrikizumab every two weeks (Q2W), 250 mg of lebrikizumab every four weeks (Q4W), and placebo every two weeks. EASI75 (achievement of ≥75% reduction in the EASI score compared to baseline) in the Q2W group was maintained by 78.4%, in the Q4W group by 81.7%, and by 66.4% in the placebo group at week 52 after drug discontinuation. Throughout the study, during both treatment induction and maintenance, 63% of patients reported adverse events (AEs), of which 93.1% were mild or moderate in severity [100]. A 2024 meta-analysis showed that lebrikizumab was not associated with significantly greater changes in EASI, POEM (Patient-Oriented Eczema Measure), DLQI (Dermatology Life Quality Index), or PPNRS (Peak Pruritus Numerical Rating Scale) scores compared anti-IL-4Rα agent dupilumab in adults with AD during a 16-week treatment period indicating that both agents are similarly effective [101]. Currently, five phase 3 trials investigating the efficiency and safety of lebrikizumab in moderate-to-severe AD are ongoing (NCT05916365, NCT04392154, NCT05369403, NCT06526182, NCT05990725).

Cendakimab selectively targets IL-13 by inhibiting its binding to receptors IL-13Rα1 and IL-13Rα2. In a phase 2 study conducted on adult patients with moderate-to-severe AD, cendakimab was demonstrated as effective, well-tolerated, and generally safe for patients. The study met its primary endpoint, i.e., significant changes in EASI score from baseline to week 16, particularly in the 720 mg Q1W group, and consistent improvements were seen across all dosing groups over the 16-week treatment period [102]. TEAEs were reported for 40 patients (74.1%) with cendakimab 720 mg Q1W, 41 (74.5%) treated with 720 mg Q2W, 38 (69.1%) treated with 360 mg Q2W, and 41 (73.2%) treated with placebo. The majority of TEAEs were mild-to-moderate in severity. Severe TEAEs were reported for nine patients (4.1%) [102].

Eblasakimab is a mAb that binds to IL-13Rα1, thereby blocking the signaling pathways of both interleukin-4 and interleukin-13. According to the results of the TREK-AD phase 2b study, eblasakimab 600 mg Q4W and 400 mg Q2W significantly improved EASI scores compared to placebo [103]. Adverse events, including conjunctivitis and injection site reactions, were slightly more frequent in the eblasakimab-treated groups than in the placebo group [103]. Currently, the phase 2 study is recruiting to evaluate the efficiency and safety of eblasakimab in adults with moderate-to-severe AD who were previously treated with dupilumab (NCT05694884).

Stapokibart (CM310) is a humanized mAb targeting IL-4Rα, similar to dupilumab. In a phase 2 study, it showed promising results in treating patients with moderate-to-severe AD. Patients received a loading dose of 600 mg subcutaneously, followed by 300 mg Q2W for up to 52 weeks [82]. At week 52, the EASI75 maintenance was 96.3%. There was also a significant reduction in PPNRS scores in the stapokibart-treated group. This study demonstrated the high efficacy of stapokibart and its favorable long-term safety profile. A total of 417 TEAEs were reported in 112 (88.2%) patients. 8.7% reported at least grade 3 TEAEs and 5.5% had at least one serious adverse event. No TEAEs leading to the treatment discontinuation or death were reported. Among the most common TEAEs were COVID-19, upper respiratory tract infection, conjunctivitis, and urinary tract infection. The majority of TEAEs were mild-to-moderate in severity [82]. Currently, phase 2 (NCT06116565) and phase 3 (NCT06495229, NCT06277765) trials are ongoing to investigate the efficiency and safety of stapokibart in moderate-to-severe AD.

Rademikibart (CBP-201) is a next-generation mAb that targets IL-4Rα. The WW001 phase 2 study evaluated its efficacy in reducing EASI scores at week 16. Patients receiving 300 mg Q2W demonstrated significant reductions in EASI scores as early as week 2. Similar efficacy was observed in the group receiving 300 mg Q4W. Both the treatment and placebo groups exhibited comparable rates of adverse events [104]. The 52-week phase 2 SEASIDE CHINE study reinforced these findings, demonstrating sustained efficacy with 300 mg Q4W dosing and clinically meaningful improvements in pruritus and QoL measures over time [105]. Currently, the phase 2 study is ongoing to evaluate the efficiency and safety of rademikibart in Chinese adults with moderate-to-severe AD (NCT05905133).

Itepekimab is a human-derived mAb against interleukin-33 (IL-33). Two phase 2 studies- one evaluating itepekimab as monotherapy and the other as a combination therapy with dupilumab- were conducted on patients with moderate-to-severe AD. Both studies were terminated after an interim analysis of the proof-of-concept study failed to demonstrate efficacy [106]. The pharmacodynamics of the drug were dose-dependent, and at a dose of 300 mg Q2W or Q4W, itepekimab achieved saturated binding to IL-33. Despite this, no clinical benefits were observed in patients who received itepekimab. The conclusion drawn from the study suggests that IL-33 is likely not a key driver in the pathogenesis of AD [106].

PF-06817924 is a high-affinity humanized mAb that binds IL-33. A phase 1 study evaluated the drug’s pharmacokinetics, immunogenicity, and pharmacodynamics across escalating single and limited repeat doses. The study included three participant groups: healthy individuals, patients with CRSwNP, and those with severe AD [107]. PF-06817924 was well tolerated across all groups, with linear pharmacokinetics observed for intravenous doses ranging from 10 mg to 1000 mg. Among the three groups, participants with AD exhibited the lowest percentage of anti-drug antibody production. Fifty-one participants (75%) in the PF-06817924 group and 25 (86.2%) in the placebo group experienced at least 1 TEAE, respectively. Most of them were mild-to-moderate in severity and were not related to the treatment. Despite favorable safety and tolerability data, late-phase studies are needed to determine its clinical efficacy in treating AD [107].

Astegolimab is a fully human IgG2 mAb that inhibits IL-33 signaling by binding to its receptor, ST2 (also known as IL-33R or IL1RL1). IL-33/ST2 signaling plays a key role in the release of inflammatory mediators, which contributes to AD pathogenesis [108]. In a phase 2 study, despite good tolerability, astegolimab at a dose of 490 Q4W did not demonstrate efficacy in improving EASI scores or other clinical endpoints, indicating limited therapeutic potential for this drug in AD. Safety profile analysis revealed that the proportion of patients who experienced at least one AE was higher in the placebo group (58.1%) compared to the astegolimab group (41%). All AEs were mild-to-moderate in severity. No death or study discontinuation due to AE was reported [108].

Melrilimab (CNTO 7160) is a mAb targeting the IL-33R. A phase 1 study evaluated its safety, tolerability, pharmacokinetics, pharmacodynamics, and immunogenicity in two parts [58]. Part 1 involved healthy participants receiving a single intravenous dose ranging from 0.001 to 10 mg/kg or placebo. Part 2 involved participants with mild asthma or AD who received three intravenous doses of 3 mg/kg or 10 mg/kg every two weeks or placebo [58]. The drug demonstrated good tolerability, with only one reported severe adverse event (cellulitis) in a participant receiving 3 mg/kg dose. Pharmacodynamic modeling suggested that a dose of 1 mg/kg administered intravenously Q2W would be sufficient to maintain sustained inhibition of p38 phosphorylation in basophils. Although the target engagement was confirmed, no clear clinical benefits were observed in AD patients, necessitating further studies to determine the drug’s efficacy [58].

Nemolizumab is a humanized mAb that blocks the α subunit of the interleukin 31 receptor (IL-31Rα). The IL-31 pathway plays a significant role in AD-related pruritus, with IL-31 being primarily secreted by Th2 cells. Its receptor, IL-31R, is expressed in various cell types, including immune cells (eosinophils), peripheral sensory nerves, and epidermal keratinocytes. When IL-31 binds to its receptor on skin sensory neurons, it mediates the sensation of itch, leading to scratching behavior. Additionally, IL-31 impairs skin barrier integrity and promotes inflammation by inducing the secretion of cytokines and chemokines [109]. Given its central role in the development of pruritus, targeting IL-31 offers significant potential for treating pruritus-related skin conditions such as AD. In the ARCADIA 1 and ARCADIA 2 phase 3 studies, patients over the age of 12 with moderate-to-severe AD, associated pruritus, and inadequate response to topical steroids were randomized into two groups: the first group received nemolizumab 30 mg subcutaneously Q4W after a starting dose of 60 mg, while the second group received a matching placebo Q4W [110]. After 16 weeks, the study results showed that Investigator Global Assessment Scale (IGA) scores improved by ≥2 points, and EASI75 was achieved in ≥75% of participants [110]. A 2022 meta-analysis reviewed the outcomes of six studies involving 14 cohorts of participants. The analysis showed significant reductions in pruritus scores on the Visual Analog Scale (VAS) and EASI scores in patients treated with nemolizumab compared to placebo. Importantly, there were no significant differences in the incidence of adverse events between the nemolizumab and placebo groups [111]. Despite the promising clinical efficiency of nemolizumab, there are increasing concerns regarding its safety profile. While most adverse events are mild and transient, including nasopharyngitis, upper respiratory tract infections, or peripheral edema, 2–5% of nemolizumab-treated patients experienced worsening asthma. However, no de novo asthma cases were reported during nemolizumab treatment [112]. The safety of nemolizumab as a biological treatment in patients with AD and comorbid asthma should be confirmed through future large-cohort trials. Currently, a phase 2 trial aiming to evaluate the efficiency and safety of nemolizumab in children aged 2–11 is enrolling (NCT04921345). Furthermore, the phase 3 trial, including adolescent and adult patients with moderate-to-severe AD, is ongoing (NCT03989206).

Another biologics targeting Th2 immunity (next to anti-IL-4 and anti-IL-13 agents) is benralizumab. Benralizumab is a mAb targeting the α chain of the IL-5 receptor (IL-5Rα). Inhibition of IL-5R leads to the depletion of eosinophils and results in the alleviation of allergic symptoms [113]. The potential efficiency of benralizumab was recently investigated in AD. However, in a 52-week phase 2 study (HILLIER trial) in patients with moderate-to-severe AD, benralizumab did not meet both primary endpoints—an IGA score 0/1 and a decrease in IGA score of ≥2 points at week 16 compared to baseline- and secondary endpoint-changes in EASI75 and EASI90 scores. Despite the lack of significant impact on disease severity, benralizumab resulted in a significant depletion of eosinophils [114].

Ligelizumab is a high-affinity anti-IgE mAb. In a randomized trial, 22 patients with moderate-to-severe AD received 280 mg of ligelizumab Q2W for 12 weeks [115]. The drug demonstrated good efficacy in suppressing serum and cell-bound IgE levels and in reducing skin reactivity in allergic skin prick tests. However, ligelizumab did not show significantly greater efficacy in alleviating AD symptoms compared to placebo. Interestingly, patients with higher baseline IgE levels responded better to the treatment than those with lower baseline IgE levels [115].

Since AD is a chronic inflammatory process mediated by T cells, targeting molecular pathways associated with T cell activation has recently emerged as a promising therapeutic avenue for the disease. One such signaling pathway is the OX40-OX40L axis. OX40 is an inducible co-stimulatory receptor expressed on the various T cell subsets, including Th2, Th1, and Th17. After antigen stimulation, OX40 is upregulated in effector and memory T cells. Upon binding with its ligand, named OX40L, expressed on antigen-presenting cells (APC), including B cells and Langerhans cells, OX40 enhances the proliferation, differentiation, and survival of pathogenic effector and memory T cells, contributing to persistent inflammation [116]. It seems that targeting the OX40-OX40L axis may be a promising therapeutic avenue to inhibit chronic T cell-mediated inflammation in AD. Amlitelimab is a mAb that targets the OX40 ligand (OX40L) in antigen-presenting cells (APCs). In a recent phase 2a study involving patients with moderate-to-severe AD, amlitelimab showed good tolerability, with no hypersensitivity events reported. Significant reductions in EASI scores were observed, with the most substantial improvements occurring between weeks 2 and 16 [117]. Currently, one phase 2 (NCT05769777), one phase 2/3 (NCT05492578), and five phase 3 (NCT06241118, NCT06224348, NCT06407934, NCT06181435, NCT06130566) trials are recruiting participants with moderate-to-severe AD to evaluate the efficiency and safety of amlitelimab.

Rocatinlimab is an anti-OX40 mAb tested in a phase 2b study involving patients with moderate-to-severe AD [118]. Rocatinilimab in doses ranging from 150 Q4W to 600 mg Q2W resulted in significant changes in EASI scores compared to baseline in all groups compared to placebo. Common adverse events included pyrexia (17% of patients), nasopharyngitis (14%), chills (11%), headache (9%), aphthous ulcers (7%), and nausea (6%) [118]. Recently, a phase 3 study (ROCKET-Horizon) evaluating the efficacy, safety, and tolerability of rocatinlimab monotherapy in adult subjects with moderate-to-severe AD has been completed, with results under development (NCT05651711). At the time of manuscript submission, six phase 3 trials investigating the efficiency and safety of rocatinlimab in moderate-to-severe AD are underway (NCT05633355, NCT06224192, NCT05398445, NCT05724199, NCT05882877, NCT05704738).

IL-36 signaling is involved in various inflammatory skin diseases, including psoriasis, hidradenitis suppurativa, and AD. IL-36 is expressed by various cell types, including keratinocytes and T cells. In AD, exposure to *S. aureus* induces IL-36 expression and promotes IL-17 secretion by T cells, contributing to chronic skin inflammation. In addition, *S. aureus*-mediated IL-36 upregulation enhances IgE production [119]. Spesolimab is an anti-interleukin 36 receptor (IL-36R) mAb. In a phase 2a study involving patients with moderate-to-severe AD, spesolimab at a dose of 600 mg every 4 weeks significantly reduced the EASI score at week 16 in corticosteroids-free patients and demonstrated a good tolerability profile. Further investigation in larger studies is required to better determine the drug’s efficacy and long-term safety in the treatment of AD [120].

YH35324 is a long-acting IgETrap-Fc fusion protein developed for allergic diseases mediated by IgE. In a phase 1 study with an active comparator as an omalizumab, the safety, tolerability, pharmacokinetics, and pharmacodynamics of YH35324 were evaluated in healthy participants and patients with mild AR, AD, food allergies, or urticaria [121]. Participants were divided into two parts: Part A included subjects with serum IgE levels between 30–700 IU/mL, and Part B included those with IgE levels > 700 IU/mL. The study demonstrated a strong safety profile, with most TEAEs being mild (grades 1–2) and no reports of serious adverse events, discontinuations, or anaphylaxis [121]. YH35324 significantly suppressed serum-free IgE levels (<25 ng/mL, *p* < 0.05) and demonstrated a longer duration of effect compared to omalizumab [121]. A phase 1b study evaluating ascending doses of YH35324 is currently underway (NCT05564221).

In recent years, there has been a growing interest in targeted therapies aimed at alarmins, a group of epithelial-derived cytokines constitutively expressed in tissues such as the respiratory epithelium and epidermis. Alarmins play a critical role in the defense against various pathogens but are also implicated in the pathogenesis of allergic diseases, including AD [122]. Among these, TSLP has garnered particular attention as a therapeutic target for AD. In the phase 2a trial (NCT02525094) involving patients with moderate-to-severe AD, treatment with anti-TSLP tezepelumab combined with topical corticosteroids (TCS) led to a reduction in EASI-50 scores compared to TCS alone. However, the improvement did not reach statistical significance. Safety analyses revealed similar rates of TEAEs between the tezepelumab and placebo groups [123]. Despite its promise, the small sample size of the trial limited definitive conclusions. A subsequent phase 2b trial (NCT03809663), designed to assess tezepelumab as both monotherapy and adjunct therapy with TCS, was terminated after failing to meet pre-established efficacy targets for the patient population. These results underscore the need for further research to clarify the role of TSLP inhibition in AD management. Another anti-TSLP agent, MK-8226/solrikitug, was investigated in the MK-8226-003 phase 1b trial (NCT01732510), which aimed to evaluate its safety, efficacy, pharmacokinetics, pharmacodynamics, and immunogenicity. However, the study was prematurely terminated due to business decisions, and no official publication of the results is available to date. Preliminary data posted on the clinical trial website suggest that solrikitug at a dose of 3 mg/kg was associated with a significant reduction in EASI scores compared to placebo after 12 weeks in patients with moderate-to-severe AD. These preliminary results of efficiency, though promising, require further validation in late-phase clinical studies.

**Table 1:** Summary of clinical trials investigating novel biologics in asthma and atopic dermatitis (2019–2024).

Drug	Molecular Target	Study Register	Study Type	Phase	Main Findings/Outcomes	Ref.
**Asthma**						
Depemokimab	IL-5	NCT04719832 NCT04718103	Randomized, double-blind, placebo-controlled, parallel-group, multicenter study	3a	Reduced AER; no improvement in QoL (SGRQ score)	[43]
Lebrikizumab	IL-13	NCT01875003	Randomized, double-blind, placebo-controlled, parallel-group, multicenter study	3	Lebrikizumab (37.5 and 125 mg) reduced exacerbation rates by 40–51%, with greater effect in patients with higher eosinophil counts (≥300 cells/μL).	[50]
Tozorakimab (MEDI3506)	IL-33	NCT04570657	Randomized, double-blind, placebo-controlled, parallel-group, multicenter study	2a	Tozorakimab improved prebronchodilator FEV1 in patients with ≥2 exacerbations but showed no overall improvement at week 16 compared to placebo.	[54]
Astegolimab	ST2/IL-33R	NCT02918019	Randomized, double-blind, placebo-controlled, multicenter, multi-arm study	2b	Astegolimab (490 mg) reduced AER by 43% overall and 54% in the eosinophil-low subgroup compared to placebo.	[61]
Itepekimab	IL-33	NCT03387852	Randomized, double-blind, placebo-controlled, parallel-group, multicenter study	2	Itepekimab monotherapy improved FEV1, asthma control, and QoL compared to placebo, with lower asthma control loss (22% vs. 41%). Adverse events were mild-to-moderate and similar across groups.	[56]
Melrilimab (CNTO 7160)	IL33R	NCT03393806	Randomized, double-blind, placebo-controlled, parallel-group, multicenter study	2a	The study terminated early due to high screen failure and low enrollment; melrilimab reduced free sST2 concentration but showed no change in eosinophil count compared to placebo at week 12.	[57]
Melrilimab (CNTO 7160)	IL33R	NCT03207243	Randomized, double-blind, placebo-controlled, parallel-group, multicenter study	2a	Melrilimab reduced asthma control loss by 18% over 16 weeks but had higher TRAE incidence (10% vs. 4%), primarily cardiac and musculoskeletal disorders.	[59]
Risankizumab	IL-23	NCT02443298	Randomized, double-blind, placebo-controlled, parallel-group, multicenter study	2a	Risankizumab was well-tolerated but associated with a shorter time to asthma worsening (HR 1.46) and higher annualized worsening rates compared to placebo.	[65]
Ecleralimab (CSJ117)	TSLP	NCT04410523	Randomized, double-blind, placebo-controlled, parallel-group, multicenter, multi-national study	2	Only the 0.5 mg dose of Ecleralimab achieved a 12% FEV1 improvement at week 8; no significant changes in FeNO were observed for any dose.	NA
REGN1908-190 (1:1 cocktail of monoclonal antibodies)	Fel d 1	NCT03838731	Randomized, double-blind, placebo-controlled, parallel-group, single-center study	2	A single dose of REGN1908/1909 prevented cat allergen-induced FEV1 decline (days 8–85) and early asthmatic response, increasing allergen tolerance on day 85 (54 vs. 12.9 ng).	[69]
MTPS9579A	Tryptase	NCT04092582	Randomized, double-blind, placebo-controlled, multicenter study	2a	MTPS9579A failed to meet the primary or secondary endpoints, showing no significant difference from placebo over 48 weeks.	[74]
**Atopic dermatitis**						
Bermekimab	IL-1α	NCT03496974 NCT04021862 NCT04791319 NCT04990440	(1) Open-label, dose-escalation study (2) Double-blind, placebo-controlled, randomized study (3) Double-blind, placebo- and active-comparator-controlled, randomized study (4) Double-blind, placebo-controlled, randomized study	2	No efficacy in larger controlled trials.	[99]
Lebrikizumab	IL-13	NCT04146363 NCT04178967	Randomized, double-blind, placebo-controlled study	3	Lebrikizumab maintained IGA and EASI-75 improvements (71.2–81.7%) with Q2W and Q4W dosing; 63% of patients reported mild-to-moderate TEAEs.	[100]
Stapokibart (CM310)	IL-4	NCT04893707	Multicenter, open-label, nonrandomized study	2	High clinical efficacy of stapokibart treatment with acceptable safety profile of the drug.	[84]
Itepekimab	IL-33	NCT03738423 NCT03736967	(1) Dose-ranging, randomized, double-blind, placebo-controlled, parallel-group study (2) Proof-of-concept randomized, double-blind, placebo-controlled study	2	Trials were prematurely terminated due to a lack of clinical benefits with itepekimab.	[106]
Nemolizumab	IL-31Rα	NCT03985943 NCT03989349	Replicate, randomized, double-blind, placebo-controlled, multicenter, parallel-group study	3	Nemolizumab significantly reduced pruritus (VAS) and improved EASI scores, with no increase in adverse event incidence compared to placebo.	[110]
Cendakimab	IL-13Rα1 and IL-13Rα2	NCT04800315	Randomized, double-blind, placebo-controlled, parallel-group, dose-ranging study	2	Cendakimab was effective, well-tolerated, and generally safe.	[102]
Rademikibart (CBP-201)	IL-4Rα	NCT04444752 NCT05017480	Randomized, double-blind, placebo-controlled, multi-centered study	2	Studies showed sustained efficacy, continued improvement in outcomes, and meaningful benefits in pruritus and QoL.	[104,105]
Benralizumab	IL-5Rα	NCT04605094	Randomized, double-blind, placebo-controlled, parallel-group, multinational study	2	Benralizumab showed no differences from the placebo in primary (IGA response) or secondary endpoints, including EASI scores, itch improvement, symptom control, and HRQoL.	[114]
Eblasakimab	IL-13Rα1	NCT05158023	Randomized, double-blind, placebo-controlled study	2b	Eblasakimab improved EASI scores (−65% vs. −27%); 71% of patients reported mild-to-moderate TEAEs compared to 47% in the placebo group.	[103]
PF-06817924	IL-33	NCT02743871	Randomized, placebo-controlled study	1	The drug was well tolerated, exhibited linear pharmacokinetics, and showed the lowest anti-drug antibody production in AD patients.	[107]
Ligelizumab	IgE	EudraCT Number 2011-002112-84	Randomized, double-blind, placebo-controlled, parallel-group, proof-of-concept study	2	Ligelizumab efficacy was not superior to placebo but showed better responses in patients with high baseline IgE levels.	[115]
Amlitelimab	OX40	NCT03754309	Multi-center, randomized, double-blind, placebo-controlled, parallel-group study	2a	Amlitelimab was well tolerated with no hypersensitivity events and showed the greatest EASI score improvements at weeks 2–16.	[117]
Rocatinlimab	OX40	NCT03703102	Multicenter, randomized, placebo-controlled, double-blind, parallel-group study	2b	Rocatinlimab improved AD symptoms, with benefits maintained after discontinuation, and was well tolerated.	[118]
Spesolimab	IL-36R	NCT03822832	Multicenter, randomized, double-blind, placebo-controlled	2a	The study showed that the drug was well tolerated, and at week 16, the EASI score decreased.	[120]
Astegolimab	IL-33/ST2	NCT03747575	Randomized, double-blind, placebo-controlled multicenter	2	Despite the good tolerability of the drug, astegolimab did not show clinical efficacy in patients with AD.	[108]
Melrilimab (CNTO 7160)	IL-33R	NCT02345928.	Randomized, double-blind, placebo-controlled study	1	The drug was well tolerated, with one severe adverse event, but showed no clear clinical activity in asthma or AD.	[58]
YH35324	IgE	NCT05061524	Randomized, double-blind, placebo/active-controlled, single ascending dose study	1	YH35324 demonstrated a good safety profile and reduced serum-free IgE levels in subjects with atopic conditions, including AD.	[121]
Tezepelumab	TSLP	NCT02525094	Randomized, double-blind, placebo-control, multicenter study	2a	Tezepelumab plus TCS increased EASI50 achievement (64.7% vs. 48.2%) without statistical significance; TEAE incidence was similar between groups.	[123]
Tezepelumab	TSLP	NCT03809663	Randomized, double-blind, placebo-controlled, dose-ranging study	2b	The study failed to meet primary endpoints, including an IGA score of 0/1 and a 75% reduction in EASI75 at week 16.	NA
Solrikitug (MK-8226)	TSLP	NCT01732510	Randomized, double-blind, placebo-controlled, multiple rising dose study	1b	Solrikitug (3 mg/kg) significantly reduced EASI scores after 12 weeks; adverse events were common (10/13 vs. 7/8) but did not lead to discontinuation.	NA

**Table 2:** Summary of ongoing clinical trials investigating novel biologics in asthma and atopic dermatitis.

Drug	Molecular Target	Study Register	Study Type	Phase	Status
**Asthma**					
MG-K10	IL-4Rα	NCT05382910	Randomized, placebo-controlled study	1b/2	Recruiting
Telikibart (GR1802)	IL-4Rα	NCT06642961	Randomized, double-blind, placebo-controlled, multicenter study	2	Recruiting
FB825	CεmX domain of membrane IgE (mIgE)	NCT05008965	Randomized, double-blind, placebo-controlled study	2	Recruiting
Stapokibart (CM310)	IL-4Rα	NCT05761028	Randomized, double-blind, placebo-controlled, multicenter study	2/3	Recruiting
Solrikitug (MK-8226)	IlL-4Rα	NCT06496607	Randomized, double-blind, placebo-controlled, multiple dose-ranging study	2a	Recruiting
Depemokimab vs. mepolizumab or benralizumab	IL-5	NCT04718389	Randomized, double-blind, double-dummy, parallel-group, multicenter, non-inferiority study	3	Active, not recruiting
Depemokimab	IL-5	NCT05243680	Open-label, single-arm, multicenter, extension study	3	Active, not recruiting
IBI3002	IL-4Rα, TSLP	NCT06213844	Randomized, double-blind, placebo-controlled, single-center, single-ascending dose study	1	Recruiting
Lunsekimig (SAR443765)	IL-13/TSLP	NCT06676319	Randomized, double-blind, placebo-controlled, parallel-group, two-arm study	2	Recruiting
Lunsekimig (SAR443765)	IL-13/TSLP	NCT06102005	Randomized, double-blind, placebo-controlled, parallel-group, dose-ranging, multicenter study	2	Recruiting
**Atopic dermatitis**					
Lebrikizumab	IL-13	NCT05916365	Interventional, single-group assignment, open-label study	3	Active, not recruiting
Lebrikizumab	IL-13	NCT06526182	Interventional, single-group assignment, open-label study	3	Recruiting
Stapokibart (CM310)	IL-4Rα	NCT06277765	Multi-center, randomized, double-blind, placebo-controlled study	3	Not yet recruiting
Telikibart (GR1802)	IL-4Rα	NCT06216392	Randomized, double-blind, placebo-controlled, multicenter study	3	Not yet recruiting
Nemolizumab	IL-31Rα	NCT03989206	Interventional, prospective, multicenter, long-term study	3	Active, not recruiting
Rademikibart (CBP-201)	IL-4Rα	NCT05905133	Interventional, single-arm, open-label, multicenter study	2	Active, not recruiting
Eblasakimab (ASLAN004)	IL-13Rα1	NCT05694884	Multicenter, randomized, double-blind, placebo-controlled, parallel-arm study	2	Recruiting
Amlitelimab	OX40	NCT06241118	Parallel-group, multinational, multicenter, randomized, double-blind, placebo-controlled study	3	Recruiting
Amlitelimab	OX40	NCT06407934	Multinational, multicenter, randomized, double-blind, placebo-controlled, parallel-group study	3	Recruiting
Amlitelimab	OX40	NCT06181435	Parallel-group, multinational, multicenter, randomized, double-blind, placebo-controlled, three-arm study	3	Recruiting
Rocatinlimab	OX40	NCT05633355	Interventional, single-group assignment, open-label study	3	Active, not recruiting
Rocatinlimab	OX40	NCT05724199	Interventional, randomized, placebo-controlled, double-blind study	3	Active, not recruiting
YH35324	IgETrap-Fc	NCT05564221	Randomized, double-blind, placebo/active-controlled, multiple ascending dose study	1	Active, not recruiting

#### 2.2.3. Role of Biomarkers in Biological Treatment of Atopic Dermatitis

Biomarkers, as discussed earlier, play a significant role in predicting treatment responses and monitoring outcomes in patients receiving biologics. Similarly, several predictive and pharmacological response biomarkers are currently under investigation in AD.

A recent meta-analysis evaluated factors associated with favorable responses to dupilumab, defined as achieving EASI75. These factors include lower BEC and clinical features such as younger age, absence of AR, and lower body mass index (BMI), indicating that dupilumab may be particularly effective in these patient subgroups [124]. Notably, other biomarkers associated with T2 inflammation, such as baseline total serum IgE, failed to predict treatment responses to dupilumab in patients with moderate-to-severe AD [124]. Recently, Wu et al. demonstrated that baseline concentrations of CD25/soluble IL-2Rα, IL-31, and IL-36β may serve as potential predictive biomarkers, as higher levels of these cytokines were associated with a better response to dupilumab [125].

For tralokinumab, the role of predictive biomarkers is less established. Based on results from a phase 2b study, increased baseline concentrations of IL-13-related biomarkers, such as periostin (≥29.8 ng/mL) and DPP-4 (≥265.8 ng/mL), were associated with greater improvements in EASI scores in the tralokinumab-treated group compared to placebo [126]. Recently, analysis using machine learning-based deep phenotyping identified eosinophilia (>6%) and EASI scores between 5.5 and 17 as risk factors for elevated IL-13 biomarkers (periostin and DPP-4) [127]. Additionally, assessing tissue IL-13 or IL-13R expression could serve as a potential common biomarker for all biologics targeting this inflammatory pathway; however, further validation is needed to confirm its utility.

On the other hand, the role of predictive biomarkers for novel investigational biologics targeting distinct inflammatory pathways remains largely unknown. For anti-IL-5 therapies like benralizumab, it appears that patients with high eosinophil counts may benefit more from treatment, but further subgroup analyses are required to confirm this. Interestingly, ligelizumab, a novel anti-IgE biologic, appears to be more effective (EASI reduction) in patients with high baseline IgE levels (>1500 IU/mL) compared to those with lower levels (<1500 IU/mL) [115]. Furthermore, recent work by Sidbury et al. suggests that increased expression of protein biomarkers, including CCL18, CCL20, CCL22, CCL27, VEGF-A, and IL-1RA, may predict a better response to nemolizumab treatment, as indicated by changes in EASI75 from baseline [128]. The results of the phase 2a ALLEVIAD study suggest that tezepelumab may be more effective (assessment with EASI50, EASI75, EASI90, and IGA scores) in certain patient subgroups, including high DPP-4, low periostin, low CCL17, and high IgE levels at the baseline [123]. 

In summary, while biomarkers such as BEC, periostin, and IgE levels have shown potential in guiding treatment decisions for established biologics like dupilumab and tralokinumab, their predictive value remains inconsistent and requires further validation. The development of reliable biomarkers for novel investigational biologics, particularly those targeting distinct inflammatory pathways, is essential to optimize patient selection and enhance therapeutic outcomes in AD.

### 2.3. Chronic Spontaneous Urticaria

#### 2.3.1. Epidemiology, Symptoms, and Current Treatment Options

Chronic spontaneous urticaria (CSU) is a chronic condition defined by recurrent itchy wheals and/or angioedema lasting for six weeks or longer without an identifiable trigger [129]. The incidence of CSU in the general population is 0.5–1%, with a lifetime prevalence of 1.4% [8]. CSU is associated with a significant reduction in QoL, together with a higher prevalence of anxiety, depression, dysregulated daily activities, and poor sleep quality [130,131]. The current first-line treatment involves the use of second-generation H1-antihistamine drugs at a standard dose, which may be increased to up to four times the daily dosage if necessary. However, up to 60% of patients with CSU remain symptomatic despite escalating therapy, requiring effective second-line treatments [132].

#### 2.3.2. Biologics in CSU-Current Recommendations and Future Perspectives

When high doses of antihistamine drugs fail to control symptoms, the current guidelines provided by the Dermatology Section of the European Academy of Allergology and Clinical Immunology (EAACI), the Global Allergy and Asthma European Network (GA^2^LEN), Urticaria and Angioedema Centers of Reference and Excellence (UCAREs and ACAREs), the European Dermatology Forum (EDF), and the Asia Pacific Association of Allergy, Asthma, and Clinical Immunology (APAAACI) recommend omalizumab as the preferred second-line treatment. Omalizumab is typically initiated at 300 mg Q4W, with the possibility of dose escalation up to 600 mg Q2W if needed [133]. Recent network meta-analyses indicate that 150 mg of omalizumab Q4W may also be effective for CSU management [134]. Omalizumab inhibits IgE, preventing the formation of wheals and angioedema, improving QoL, and offering long-term efficacy [133]. It significantly improves Urticaria Activity Score (UAS) and weekly UAS (UAS7) scores, with an average complete response rate of 72.2% [135]. Omalizumab also reduces the use of rescue medications and the occurrence of drug-related serious AEs [136]. Although guidelines do not recommend measuring IgE concentrations before initiating omalizumab, recent meta-analyses suggest that baseline total IgE levels may serve as biomarkers to predict treatment response. However, the precise IgE threshold favoring omalizumab response remains unknown [137].

At present, omalizumab is the only biological treatment approved for CSU. However, several other biologics are currently under investigation. The results of recently published trials on novel biologics for CSU are presented in Table 3, while ongoing trials are summarized in Table 4. The detailed summary of recently published and currently ongoing trials for CSU are presented in Table S1 and Table S2, respectively.

Dupilumab modulates the signaling pathways of IL-4 and IL-13 through inhibition of IL-4Rα [138]. It is FDA-approved for the treatment of AD, asthma, CRSwNP, eosinophilic esophagitis, prurigo nodularis, and chronic obstructive pulmonary disease [97]. The efficacy and safety of dupilumab in CSU are currently under investigation. Recently, the results of two phase 3 trials, LIBERTY-CSU CUPID A and B, were published. Study A enrolled patients who were omalizumab-naive, while Study B focused on patients who were either intolerant or incomplete responders to omalizumab. Participants in both trials remained symptomatic despite the regular use of H1-antihistamines. In Study A, dupilumab significantly improved both UAS7 and Itch Severity Score (ISS7) scores compared to placebo at week 24, while in Study B, UAS7 showed significant improvement, but ISS7 changes did not reach statistical significance at week 24 [139]. Notable, dupilumab improved ISS7, UAS7, and hives severity score over 7 days (HSS7) scores regardless of baseline serum IgE level (<100 or >IU/mL) [140]. Currently, an ongoing phase 3 trial named LIBERTY-CSU CUPIDKids is evaluating the pharmacokinetics and safety of dupilumab in children aged 2-12 years (NCT05526521).

Benralizumab is a novel therapy approved for severe eosinophilic asthma and eosinophilic granulomatosis with polyangiitis. Benralizumab reduces eosinophil activity by modulating the IL-5R signaling pathway [141]. Since eosinophils play a role in CSU pathogenesis, their depletion could alleviate symptoms. However, results from the phase 2b ARROYO trial demonstrated that benralizumab did not significantly improve ISS7 or UAS7 scores compared to the placebo, although it effectively depleted blood eosinophils [142].

YH35324 is a long-acting fusion protein showing promise in IgE-mediated allergic diseases. It works through a dual mechanism: binding serum-free IgE and eliminating anti-FcεRIα autoantibodies [143]. Preclinical studies have demonstrated that YH35324 has a stronger binding affinity to human IgE compared to other anti-IgE agents like omalizumab [143]. Phase 1 single-ascending dose trials have shown that YH35324 has a favorable safety profile and effectively reduces serum-free IgE levels in individuals with allergic diseases, including CSU [121]. An ongoing phase 1b trial is investigating multiple ascending doses of YH35324 in individuals with CSU and other allergic conditions (NCT05564221).

Ligelizumab is a next-generation fully human anti-IgE mAb with higher affinity compared to omalizumab. Ligelizumab suppresses IgE-dependent allergic responses more effectively due to its distinct inhibition profile [144]. Its efficacy in CSU has been explored in several clinical trials. A phase 2b dose-finding trial showed that ligelizumab might outperform standard-dose omalizumab with a comparable safety profile [145]. However, phase 3 trials, PEARL-1 and PEARL-2, revealed that ligelizumab at doses of 72 mg and 120 mg was not superior to 300 mg omalizumab in improving UAS7 scores at week 12 [146]. A recent analysis suggested that while ligelizumab doses of 72 mg and 120 mg might outperform 150 mg omalizumab, the 300 mg dose of omalizumab remains more effective for CSU treatment [147].

UB-221 is an IgE-neutralizing mAb with a distinct pharmacodynamic profile compared to omalizumab and ligelizumab [148]. In a phase 1 trial, UB-221 was associated with reductions in UAS7 scores and serum-free IgE levels, with no serious adverse events or infusion reactions reported [148]. Currently, a phase 1 randomized trial is underway to evaluate the safety, tolerability, pharmacokinetics, and pharmacodynamics of a single dose of UB-221 as add-on therapy for antihistamine-refractory CSU (NCT04175704).

CMAB007, also referred to as omalizumab α, is a biosimilar to omalizumab. Early-phase trials have shown it to be effective and safe in reducing free IgE levels in healthy participants [149]. A phase 3 trial is currently recruiting to compare CMAB007 with original omalizumab (Xolair^®^) in terms of efficacy, immunogenicity, pharmacokinetics, pharmacodynamics, and safety (NCT06365879).

Lirentelimab, a humanized mAb targeting sialic acid-binding Ig-like lectin 8 (Siglec-8) expressed on eosinophils, basophils, and mast cells, is a novel therapeutic option for mast cell-mediated diseases like CSU. By binding Siglec-8, lirentelimab induces eosinophil depletion, inhibits IgE-mediated histamine, prostaglandin D, and chemokine release, and suppresses IL-33-mediated neutrophil recruitment [150]. In the CURSIG phase 2a study, lirentelimab significantly reduced the UAS7 score in antihistamine-refractory CSU, including omalizumab-naive and omalizumab-refractory patients [151]. However, the phase 2 MAVERICK trial was prematurely terminated. A post-hoc analysis of the MAVERICK trial posted on ClinicalTrials.gov suggests that the study did not meet its primary endpoint, a change from baseline in UAS7 scores at week 12 (NCT05528861).

Canakinumab is an interleukin 1β (IL-1β) blocker with significant anti-inflammatory properties approved for periodic fever syndromes, active Still disease, and gout flares [152]. URTICANA trial demonstrated that canakinumab had no significant effect on CSU symptoms, including wheals and angioedema, suggesting that IL-1β is not likely to play a key role in the pathogenesis of CSU [153].

### 2.4. Eosinophilic Gastritis, Enteritis, and Colitis

#### 2.4.1. Classification, Prevalence, Symptoms, and Treatment

Eosinophilic gastrointestinal disorders (EGIDs) are rare, chronic conditions characterized by abnormal eosinophil accumulation in various segments of the gastrointestinal (GI) tract, resulting in inflammation and tissue injury [154]. Based on a recent expert consensus, four main types of EGIDs have been identified: eosinophilic esophagitis (EoE) and non-EoE-EGIDs, which include eosinophilic gastritis (EoG), eosinophilic enteritis (EoN), and eosinophilic colitis (EoC). This classification emphasizes the importance of specifying the location of active eosinophilic inflammation in the GI tract during diagnosis [155]. Non-EoE-EGIDs are rare, with a prevalence of fewer than 10 cases per 100,000 people in Western countries. However, some studies suggest that nearly 2% of patients with suspected GI conditions may have non-EoE-EGIDs [20]. These conditions present a broad spectrum of symptoms that vary depending on the affected region, including abdominal pain, diarrhea, nausea, and vomiting. Such symptoms often overlap with other GI disorders, complicating diagnosis [20,154]. Treatment options for non-EoE-EGIDs remain challenging due to the absence of standardized therapeutic guidelines [156]. Management strategies are typically individualized, taking into account symptom severity and the extent of eosinophilic involvement [157]. Glucocorticosteroids are the first-line treatment, providing effective control of inflammation and symptom relief for most patients. However, their chronic use is associated with significant side effects, prompting the need for alternative approaches [158].

#### 2.4.2. The Role of Biologics in Non-EoE-EGIDs-Current State and Perspectives

Biological treatments are emerging as a key area of interest in the management of non-EoE-EGIDs. Although there are no official treatment guidelines, ongoing clinical trials and case reports indicate the efficiency of several biological agents targeting eosinophilic inflammation [156]. The results of recently published trials on novel biologics for non-EoE-EGIDs are presented in Table 3, while ongoing trials are summarized in Table 4.

Omalizumab is a mAb that binds specifically to circulating IgE, preventing its interaction with the high-affinity FcεRI receptor on mast cells and basophils. While omalizumab shows promise in allergic-type inflammation, its efficacy in non-EoE-EGIDs requires further validation [159]. In a trial involving nine patients with eosinophilic gastroenteritis (EGE), omalizumab significantly reduced peripheral eosinophil counts, but its effects on tissue eosinophilia were minimal. Some symptoms improved, but the results were inconsistent, emphasizing the importance of patient selection [160,161]. A recent case study reported a 33-month-old boy diagnosed with EoG who experienced symptom relief and reduced peripheral eosinophilia after receiving 150 mg of omalizumab Q2W. Despite these positive outcomes, the broader efficacy of omalizumab in treating active non-EoE-EGIDs remains unclear and requires additional confirmation [162]. Further research is needed to explore its potential, particularly in combination with therapies targeting multiple pathways driving eosinophilia.

Reslizumab is a humanized mAb targeting IL-5, a cytokine critical for eosinophil activation and survival. While its efficacy in eosinophilic asthma is well established, data on its use in non-EoE-EGIDs remain limited to case reports [163]. A pilot study by Prussin et al. demonstrated that reslizumab decreased both tissue and blood eosinophilia in patients with EGE. However, symptom improvement was minimal, underscoring the multifactorial nature of the disease [164]. Conversely, Kim et al. reported significant symptomatic and eosinophilic improvements in patients with EGE or hypereosinophilic syndrome, although rebound hypereosinophilia occurred in both studies [165]. These findings highlight the need for broader clinical trials to clarify reslizumab’s therapeutic potential.

Lirentelimab (AK002), an anti-Siglec-8 biologics due to its antagonist activity against eosinophils, basophils, and mast cells, is a promising therapy for allergic and eosinophilic diseases, including non-EoE-EGIDs [166]. Lirentelimab has demonstrated promising efficacy in phase 2 trials for the treatment of moderate to severe EoG and eosinophilic duodenitis (EoD) [167]. In the lirentelimab-treated groups, a significant reduction in tissue eosinophils was observed, with a mean 95% decrease compared to a 10% increase in the placebo group [167]. Despite encouraging phase 2 findings, the phase 3 ENIGMA2 trial produced mixed results (NCT04322604). While lirentelimab continued to demonstrate efficacy in reducing eosinophil counts in GI tissues, with 85% of the active treatment group achieving histological response compared to 5% in the placebo group, it failed to achieve statistically significant improvements in patient-reported symptomatic relief. These findings suggest that while lirentelimab is highly effective in reducing tissue eosinophilia, further research is needed to better understand its impact on symptom relief and overall clinical efficacy.

Benralizumab is an eosinophils-depleting mAb that acts through antibody-dependent cell-mediated cytotoxicity (ADCC) [168]. This mechanism makes it a compelling candidate for treating eosinophilic GI conditions. One trial (NCT05251909) aimed to evaluate the efficacy and safety of benralizumab in patients with EoG or EoE. However, the trial was prematurely discontinued by the sponsors based on findings from other independent studies. Related studies have provided valuable insights into benralizumab’s potential. For instance, an open-label study by Kuang et al. evaluated benralizumab in patients with hypereosinophilic syndrome (HES). The study demonstrated complete depletion of blood and tissue eosinophils, although clinical responses varied significantly among participants, highlighting the heterogeneity of treatment outcomes [169]. Similarly, a phase 2 trial (NCT03473977) assessed benralizumab in patients with EoG. Most participants achieved remission with a substantial reduction in gastric eosinophil counts compared to placebo. Benralizumab effectively depleted eosinophils in blood and tissue, and it improved histologic indices of GI inflammation [170]. Despite these promising results, persistent histologic, endoscopic, and molecular abnormalities, as well as lingering patient-reported symptoms, suggest that eosinophil-independent mechanisms may contribute to the disease pathogenesis. Post-hoc analyses have further confirmed that eosinophil depletion alone is insufficient to fully normalize disease features or consistently improve symptoms in many patients [170]. These observations emphasize the need for therapeutic strategies targeting broader type 2 immune pathways in EGIDs.

Preliminary data from ongoing trials (NCT03678545, NCT05831176) indicate that dupilumab may induce remission in EoG and EoD. A case study described a 34-year-old female with EoG who experienced symptom alleviation and histological remission after one month of dupilumab treatment [171]. Another study on 12 patients demonstrated significant reductions in eosinophil counts and symptom scores, with complete symptom remission in 40% of EoG patients and 33.3% of EoD patients [172]. These results suggest that the ongoing trials may find favorable results supporting the idea that dupilumab is safe and effective for inducing remission in patients with non-EoE-EGIDs.

The application of mepolizumab in non-EoE-EGIDs is still under investigation [156]. To date, most of the evidence comes from case reports or series. One case highlighted rapid symptom resolution and decreased eosinophil counts in a 46-year-old patient with EoG treated with a single 300 mg dose of mepolizumab [173]. This case highlights mepolizumab’s potential in managing EGIDs, but further studies are needed to establish its role as a standard treatment. Currently, cendakimab is being evaluated in a phase 3 trial to assess its remission in adults and adolescents with eosinophilic gastroenteritis (EGE) (NCT05214768).

Vedolizumab, a humanized mAb targeting α4β7 integrins on T lymphocytes, exhibits selective anti-inflammatory effects specifically within the gastrointestinal tract [174]. Although no formal clinical trials are currently underway, evidence from case reports and small-cohort retrospective studies suggests its potential efficacy in non-EoE-EGIDs. In a case series involving five patients with non-EoE-EGIDs who received vedolizumab off-label after failing all available therapies, clinical and histological improvements were observed in two patients. These improvements allowed for a reduction in SCS use [175]. A retrospective cohort study of 22 patients (including four steroid-refractory patients) with EGE further supports vedolizumab’s potential benefits. Clinical and histological improvements were observed in 75% of steroid-refractory cases [176]. The limited sample sizes and retrospective nature of these analyses underscore the need for larger, well-designed clinical trials to thoroughly evaluate vedolizumab’s safety, efficacy, and long-term outcomes in non-EoE-EGIDs [177].

### 2.5. Chronic Rhinosinusitis with Nasal Polyps

#### 2.5.1. Symptoms and Treatment Strategies

Chronic rhinosinusitis (CRS) is a group of diseases characterized by persistent inflammation affecting the paranasal sinuses [178]. CRSwNP is predominantly a Th2-dominant inflammatory process characterized by tissue eosinophilia and increased tissue mast cells, ILC2 cells, local IgE, and Th2 cytokines [178]. Among CRS patients, approximately 25–30% suffer from chronic rhinosinusitis with nasal polyps (CRSwNP). Nasal polyps are inflamed growths that extend into the nasal airway, usually bilaterally, and arise from the ethmoid sinus. The hallmark symptoms of CRSwNP include anterior or posterior rhinorrhea, nasal congestion, hyposmia, and/or facial pressure or pain [179]. CRSwNP affects approximately 2–4% of adults, significantly impairing QoL [180]. Despite the lack of a definitive cure, various management strategies are available [181].

Treatment strategies for CRSwNP typically include long-term intranasal corticosteroids (INCS), short courses of SCS, aspirin therapy, and, in severe cases, multiple sinonasal surgeries [182]. According to evidence-based guidelines [181], biologics are recommended over non-biologic therapies in specific cases. The decision to initiate biologics is guided by factors such as inadequate response to INCS after at least 4 weeks, higher baseline disease severity, patient preference to avoid the inefficacy or challenges of alternative therapies, and the convenience of avoiding frequent payments or insurance complexities. Biologics are also considered for patients who experience symptom improvement with other treatments like INCS, surgery, or aspirin therapy after desensitization, as well as those with comorbid atopic conditions such as asthma or other atopic diseases.

#### 2.5.2. Biological Treatment-Approved and Investigational Agents

The efficacy of biologics varies, influencing the selection of therapy. A network meta-analysis by Oykhman et al. [183] identified dupilumab and omalizumab as the most effective biologics for patient-relevant outcomes, including nasal symptom improvement, with mepolizumab also showing benefits [181]. The EPOS/EUFOREA guidelines recommend biological treatment for patients meeting ≥3 of the following five criteria [184]: evidence of type 2 inflammation, such as tissue eosinophils >10/hpf, blood eosinophils >150, or total IgE >100 IU/mL; a need for SCS, with two or more courses required per year, or contraindications to SCS use; significantly impaired QoL indicated by a SNOT-22 score greater than 40; significant loss of smell; or the presence of comorbid asthma.

Dupilumab is FDA-approved for treating type 2 inflammation-driven diseases, including AD and asthma. Dupilumab has been shown to alleviate symptoms of nasal congestion, obstruction, nasal discharge, and olfactory impairment in individuals with CRSwNP. It also enhances health-related quality of life (HR-QoL), reduces SCS use, and decreases the need for surgical intervention [185]. A phase 4 multicenter observational study involving 648 patients confirmed its safety and effectiveness in reducing polyp size, alleviating symptoms, and enhancing QoL over one year [186]. Additional evidence suggests that dupilumab reduces upper and lower respiratory tract infections in CRS patients [187]. Notably, its effectiveness is independent of prior surgical intervention and may be enhanced when used earlier post-surgery [188]. Comparative studies indicate that dupilumab outperforms omalizumab and mepolizumab in both primary and secondary outcomes for CRSwNP [189].

Omalizumab, another FDA-approved biologic used in CRSwNP, is currently under evaluation in the global phase 4 EVEREST trial, which seeks to compare its efficacy with dupilumab in patients with severe CRSwNP and comorbid asthma [190]. Notably, omalizumab has shown no increase in congenital anomalies or adverse outcomes in a registry of pregnant individuals with asthma treated with the drug, demonstrating its safety profile [191].

Mepolizumab was approved by the FDA for adults with CRSwNP as an add-on therapy to INCS [192]. Mepolizumab has shown significant efficacy and tolerability in patients with CRSwNP. As a mAb targeting IL-5, mepolizumab works by reducing eosinophilic inflammation, which plays a critical role in the pathogenesis of CRSwNP. Two phase 3 studies demonstrated that mepolizumab significantly improved clinical outcomes, including enhancing the sense of smell in patients with CRSwNP [193,194]. In addition, mepolizumab reduces the reliance on SCS, providing clinical benefits regardless of prior corticosteroid use [195]. The SYNAPSE trial further established mepolizumab’s efficacy in decreasing the risk of subsequent sinus surgeries in patients with recurrent, refractory, severe CRSwNP. Notably, clinical benefits from the treatment persisted for up to 24 weeks following cessation, as observed in the 52-week SYNAPSE study [196,197]. Several ongoing trials are investigating the potential of mepolizumab in various CRSwNP treatment scenarios. A multicenter phase 4 trial (NCT05923047) is examining differences in outcomes among patients who undergo nasal polypectomy followed by mepolizumab treatment, those receiving mepolizumab alone, and those treated solely with nasal polypectomy. Another study (NCT06069310) aims to compare the effects of mepolizumab in patients with CRSwNP and comorbid asthma, CRSwNP without asthma, and healthy individuals. There is an ongoing clinical trial (NCT05598814) to compare outcomes between mepolizumab monotherapy and mepolizumab combined with Functional Endoscopic Sinus Surgery (FESS) in patients with CRSwNP and asthma. These trials highlight the continued exploration of mepolizumab’s role in improving clinical outcomes for patients with CRSwNP under different therapeutic contexts.

Benralizumab is another mAb whose clinical potential in CRSwNP is currently being investigated. At the time of this manuscript, benralizumab has not been officially approved for CRSwNP treatment. The phase 3 OSTRO trial demonstrated benralizumab effectiveness in reducing eosinophils in both blood and nasal polyps [198]. The same study showed that benralizumab significantly improved nasal polyps score (NPS), nasal blockage score, and sense of smell score compared to placebo at week 40 [199]. While a Japanese phase 2 trial showed limited efficacy in primary endpoints, improvements in nasal polyps were observed [200]. The ANDHI phase 3b trial highlighted its benefits in patients with severe eosinophilic asthma and coexisting CRSwNP, improving SNOT-22 score and asthma outcomes [201].

PF-06817024 is a humanized mAb with a high affinity for IL-33, a proinflammatory cytokine associated with type 2 inflammation. Phase 1 study was carried out with results showing that PF-06817024 was generally well tolerated in patients with CRSwNP; it showed an acceptable safety profile [107]. Currently, there are two ongoing phase 2 trials (NCT06451640, NCT06036927) on the drug TQC2731 in the treatment of CRSwNP. TQC2731 inhibits TSLP, an epithelial cell-derived cytokine implicated in the initiation and persistence of Th2-inflammation in diseases like asthma [202]. Another ongoing phase 1 study, NCT05355207, analyzes the safety and activity of calpurbatug (TRL1068) in CRSwNP. Defects in the epithelial cell barrier, an increase in exposure to pathogenic and colonized bacteria, and dysregulation of the host immune system are all thought to play prominent roles in CRSwNP pathogenesis [203]. Colonization with *S. aureus* or *P. aureus* is associated with recalcitrant disease and biofilm formation, making eradication difficult. TRL1068 is anticipated to disrupt the biofilm that shields pathogens, significantly increasing the bacteria’s vulnerability to standard antibiotic therapies [204]. A phase 3 study, NCT04851964, designed to evaluate the efficacy and safety of tezepelumab in adults with severe CRSwNP, is being carried out in different parts of the world. To date, tezepelumab has shown efficacy in severe, uncontrolled asthma and may, due to common pathogenetic pathways, provide similar benefits in CRSwNP [78]. TQH2722, a novel IL-4Rα inhibitor, is currently being studied in phase 2 trials for CRS (with or without nasal polyps) (NCT06089278, NCT06439381). Summarizing, Table 3 presents the outcomes of recently published trials on novel biologics for CRSwNP, while Table 4 provides an overview of ongoing trials.

### 2.6. Allergic Rhinitis

#### 2.6.1. Clinical Presentation and Treatment Strategies

Allergic rhinitis (AR) is among the most common allergic diseases globally and is typically triggered by inhalant allergens. AR is caused by inflammation of the nasal mucosa and manifests through symptoms such as watery nasal secretions, often running down the back of the throat, nasal congestion, and sneezing. These symptoms can significantly impair the QoL and daily functioning [205]. The Korean Academy of Asthma, Allergy, and Clinical Immunology (KAAACI) has issued updated guidelines outlining the latest treatment recommendations for AR [206]. Both intranasal antihistamines (INAH) and oral antihistamines (OAH) are considered primary treatments, particularly in mild cases. For moderate and severe cases, INCS are recommended as the first-line treatment, given their ability to reduce nasal mucosa inflammation [206]. In cases where monotherapy with INCS is insufficient, combining INCS with INAH or OAH is advised [206]. However, KAAACI guidelines do not currently include recommendations on biological therapies for AR.

#### 2.6.2. The Role of Biologics in AR-Efficiency of Available Agents and Current Investigations

To date, biologics have not been approved by either the FDA or EMA for AR treatment. However, emerging evidence suggests the potential role of biologics in AR management. Table 2 presents the outcomes of recently published trials on novel biologics for AR, while Table 4 provides an overview of ongoing trials. 

Omalizumab has effectively controlled AR symptoms and improved patients’ QoL [207]. A meta-analysis of 12 randomized clinical trials showed that omalizumab treatment alleviated nasal and ocular symptoms, enhanced QoL, and reduced the need for H1 antihistamines in patients unresponsive to conventional therapies [208].

Dupilumab has also been studied for its role in AR. Phase 3 study SINUS-24 focused on patients with CRSwNP with coexisting AR and patients with CRSwNP without AR provided data for preliminary analysis of the effect after 24 weeks of dupilumab [209]. Dupilumab was shown to reduce symptom intensity, levels of circulating inflammatory biomarkers, frequency of CS use, and frequency of sinus surgery interventions compared to placebo. The effect was observed in both study groups. An ongoing SINUS-52 trial with a longer follow-up period of 52 weeks is expected to provide further data supporting dupilumab’s efficacy in ANN [209].

Another promising biologic is tezepelumab. A recent trial evaluated the effect of year-long tezepelumab combined with subcutaneous allergen immunotherapy (SCIT) in cat-allergen-induced ANN [210]. The combination therapy group showed reduced transcription of the tryptase gene TPSAB1, increased cat IgG4/IgE ratio, and decreased clinical symptoms up to one-year post-treatment [210]. These findings suggest that combined therapy modifies the nasal mucosa’s immune environment, enhancing immunotherapy’s effects [210]. A phase 2 study (NCT06189742) is currently underway to explore tezepelumab’s efficacy in severe asthma with coexisting AR.

REGN1908-1909, an anti-Feld1 (*Felis domesticus* allergen 1) mAb, was evaluated in a phase 1b trial for cat-allergen-induced AR [211]. REGN1908-REGN1909 is designed to inhibit the allergic reaction after exposure to cat allergens. The follow-up exposure to cat allergen extracts one week after admission of a single dose of REGN1908-1909 showed a significant clinical response. The suppression of the immune response after REGN1908-1909 application led to reduced type 2 cytokines (IL-4, IL-5, IL-13) and chemokines (CCL17/TARC, CCL5/RANTES) production, as well as inhibition of the allergic response mediated by FcεRI and FcεRII (CD23) and T-cell activation. While promising, further large-scale trials are needed to confirm its efficacy and safety [211].

Stapokibart (CM310), a human mAb targeting IL-4Rα, was evaluated in a phase 2 trial for uncontrolled seasonal AR (SAR) [212]. While stapokibart was well tolerated, it did not significantly improve clinical outcomes or QoL [212]. Recently, a phase 3 trial, including patients with inadequately controlled AR despite standard-of-care treatment, has been completed with no official results presented at the time of this manuscript submission (NCT05908032). Currently, three phases 2 (NCT06171074, NCT06300203, NCT06525597) are recruiting patients with AR to evaluate the efficacy and safety of stapokibart. Telikibart (GR1802) is another novel mAb targeting IL-4Rα. Currently, its efficiency and safety are being investigated in various allergic diseases including uncontrolled/poorly controlled AR (phase 2 trials: NCT06315426, NCT06028490), asthma (phase 2 trial: NCT06642961), AD (phase 3 trial: NCT06216392), and CRswNP (phase 2 trials: NCT06015243, NCT05873803; phase 3 trial: NCT06516302). 

REGN5713-5714-5715, a combination of monoclonal antibodies targeting Bet v 1 (the major allergen from *Betula verrucosa*) [213], was evaluated in the phase 3 trial NCT04709575 for its potential to alleviate symptoms of SAR in patients allergic to birch pollen. While no official publication detailing the results of this study is currently available, an analysis of data posted on the ClinicalTrials website suggests promising outcomes. Patients who received a single dose of REGN5713-5714-5715 experienced superior symptom control compared to placebo, as assessed by Total Nasal Symptom Score (TNSS), Total Ocular Symptom Score (TOSS), Daily Medication Score (DMS), and TEAEs. These results highlight the potential of REGN5713-5714-5715 as a therapeutic option for SAR. However, further research involving larger patient cohorts is necessary to confirm these findings and establish its clinical efficacy and safety profile.

**Table 3:** Summary of clinical trials investigating novel biologics in CSU, non-EoE-EGIDs, CRSwNP, and AR (2019–2024).

Drug	Molecular Target	Study Register	Study Type	Phase	Main Findings/Outcomes	Ref.
**Chronic spontaneous urticaria**						
Dupilumab	IL-4Rα	NCT04180488	Randomized, double-blind, placebo-controlled, parallel-group, multicenter study	3	In study A, UAS7 and ISS7 improved significantly vs. placebo, while study B showed minor, non-significant improvements.	[139]
YH35324	IgE	NCT05061524	Randomized, double-blind, placebo/active-controlled, single ascending dose study	1	YH35324 showed no serious AEs, discontinuations, or anaphylaxis and suppressed serum-free IgE longer than omalizumab.	[121]
Benralizumab	IL-5Rα	NCT04612725	Randomized, double-blind, placebo-controlled, parallel-group, multicenter study	2b	Benralizumab showed no changes in ISS7 or UAS7 scores at week 12 but significantly depleted blood eosinophil levels at week 24.	[142]
Ligelizumab	IgE	NCT03580369 NCT03580356	Randomized, double-blind, placebo-controlled, parallel-group, multicenter study	3	Both studies demonstrated significant improvement in UAS7 scores compared to placebos but not in omalizumab.	[146]
Lirentelimab	Siglec-8	NCT03436797	Open-label study	2a	Lirentelimab reduced disease activity (73% in omalizumab-naive; 47% in refractory) with UAS7 responses of 77% and 45% and no serious AEs.	[151]
Canakinumab	IL-1β	NCT01635127	Randomized, double-blind, placebo-controlled, single-center study	2	Canakinumab showed no significant improvements in UAS7 or clinical outcomes compared to placebo but was well tolerated with mild AEs.	[153]
**Non-esophageal eosinophilic gastrointestinal disorders**						
Lirentelimab (AK002)	Siglec-8	NCT04322604	Multi-center, randomized, double-blind, placebo-controlled	3	Lirentelimab met the primary endpoint for gastric eosinophils but failed to alleviate patient-reported symptoms.	NA
Lirentelimab (AK002)	Siglec-8	NCT04856891	Multi-center, randomized, double-blind, placebo-controlled	3	The trial met the histologic endpoint for duodenal eosinophils (≤15 eos/hpf) but failed to achieve significance in patient-reported symptoms.	NA
Lirentelimab (AK002)	Siglec-8	NCT04620811	Multi-center, open-label, extension study	3	Extended lirentelimab treatment improved TSS but showed lower eosinophil response compared to the placebebo+lirentelimab group (96.3% vs. 92.3%), with lower TAEA incidence compared to the placebo group (34.5 vs. 44%).	NA
Benralizumab	IL-5Rα	NCT03473977	Randomized, double-blind, placebo-controlled	2	Benralizumab improved remission rates (77% vs. 8%), EoG histology, inflammatory scores, and eosinophil levels, with no significant changes in SODA or PROMIS scores.	[170]
**Chronic rhinosinusitis with nasal polyps**						
Dupilumab	IL-4Rα	NCT04181190	Real-life, observational, multicenter study	4	Dupilumab reduced polyp size and improved QoL, severity of symptoms, nasal congestion, and smell.	[186]
Dupilumab	IL-4Rα	NCT02912468 NCT02898454	Randomized, multicenter, double-blind, placebo-controlled, parallel-group studies	3	Dupilumab improved NC, NPS, and LMK scores, particularly in patients with recent sinonasal surgery (<3 years), and reduced SCS use and sinonasal surgery rates compared to placebo.	[188]
Mepolizumab	IL-5	NCT04607005	Randomized, double-blind, placebo-controlled, parallel-group	3	Mepolizumab significantly improved nasal obstruction and ENPS scores and was well-tolerated with no serious adverse events in CRSwNP/ECRS patients.	[194]
Mepolizumab	IL-5	NCT03085797	Randomized, double-blind, placebo-controlled, parallel-group, multi-center trial	3	Mepolizumab improved nasal polyp size, obstruction, and smell/taste loss over 52 weeks, reduced SCS use (37.5% vs. 25.4%), and lowered prednisolone-equivalent doses regardless of prior sinus surgeries or eosinophilia.	[193,195]
Mepolizumab	IL-5	NCT03085797	Randomized, double-blind, placebo-controlled study	3	Mepolizumab provided partially sustained clinical benefits in symptoms, QoL, and corticosteroid use up to 24 weeks post-discontinuation compared to placebo.	[197]
Benralizumab	IL-5Rα	NCT03170271	Randomized, double-blind, placebo-controlled, parallel-group, multicenter study	3b	Benralizumab improved nasal symptoms in patients with high SNOT-22 scores, reduced AER by 69%, and enhanced SGRQ, FEV1, and ACQ-6 scores, with similar adverse event incidence to placebo.	[201]
Benralizumab	IL-5Rα	NCT03401229	Randomized, double-blind, placebo-controlled, parallel-group, international, multicenter study	3	Benralizumab nearly eliminated blood eosinophils (weeks 16–56) and reduced nasal polyp tissue eosinophils to 0 cells/mm^2^ at week 56 compared to placebo.	[198]
Benralizumab	IL-5Rα	NCT03401229	Randomized, double-blind, placebo-controlled, parallel-group, multi-center trial	3	Over 40 weeks, benralizumab improved NPS, nasal blockage, and sense of smell scores compared to placebo and was well tolerated, with mostly mild-to-moderate adverse events in both groups (77.3% vs. 78.8%).	[199]
PF-06817024	IL-33	NCT02743871	Randomized, double-blind, third-party open, placebo-controlled, dose-escalating study	1	Of 20 randomized CRSwNP patients, 16 completed treatment; 45.5% experienced mild-to-moderate TEAEs, primarily general disorders and administration site conditions.	[107]
**Allergic rhinitis**						
Dupilumab	IL-4Rα	NCT02912468 NCT02898454	Randomized, double-blind, placebo-controlled, parallel assignment study	3	Dupilumab improved CRSwNP measures and reduced steroid use and surgical treatment compared to placebo.	[209]
Tezepelumab	TSLP	NCT02237196	Randomized, triple-masking, placebo-controlled, parallel assignment study	2	Tezepelumab enhanced SCIT effectiveness and reduced clinical responses up to one-year post-therapy in AR patients.	[210]
REGN1908-1909	Fel d 1	NCT02127801	Randomized, double-blind, placebo-controlled study	1b	A single dose of REGN1908-1909 reduced nasal symptoms and inhibited FcεRI-, FcεRII-, and T-cell-mediated allergic responses.	[211]
Stapokibart (CM310)	IL-4Rα	NCT05470647	Randomized, double-blind, placebo-controlled, parallel assignment study	2	Administration of stapokibart did not significantly improve the clinical status of patients with uncontrolled seasonal AR.	[212]
REGN5713-5714-5715	Bet v 1	NCT04709575	Randomized, double-blind, placebo-controlled, parallel-group study	3	A single dose of REGN5713-5714-5715 improved symptom control compared to the placebo group.	NA

**Table 4:** Summary of ongoing clinical trials investigating novel biologics in CSU, non-EoE-EGIDs, CRSwNP, and AR.

Drug	Molecular Target	Study Register	Study Type	Phase	Status
**Chronic spontaneous urticaria**					
Dupilumab	IL-4Rα	NCT05526521	Single-arm, multi-center study	3	Recruiting
YH35324	IgE	NCT05564221	Randomized, double-blind, placebo/active-controlled, multiple ascending dose	1b	Active, not recruiting
UB-221	IgE	NCT04175704	Randomized, single-blind, placebo-controlled, parallel-group, single ascending dose study	1	Not yet recruiting
CMAB007	IgE	NCT06365879	Randomized, double-blind, positive parallel controlled, multicenter study	3	Recruiting
**Non-esophageal eosinophilic gastrointestinal disorders**					
Dupilumab	IL-4Rα	NCT03678545	Multi-center, randomized, double-blind, placebo-controlled trial	2	Active, not recruiting
Dupilumab	IL-4Rα	NCT05831176	Randomized, double-blind,placebo-controlled, 3-part study	2/3	Recruiting
Cendakimab	anti-IL-13Rα1 and α2	NCT05214768	Multicenter, randomized, double-blind, placebo-controlled trial	3	Active, not recruiting
**Chronic rhinosinusitis with nasal polyps**					
Dupilumab vs. Omalizumab	IL-4Rα	NCT04998604	Multicenter, randomized, double-blind, active-controlled trial	4	Active, not recruiting
TQC2731	anti-TSLP	NCT06451640 NCT06036927	Multicenter, randomized, double-blind, placebo-controlled	2	Recruiting
Calpurbatug (TRL1068)	Pathogen-protecting biofilm	NCT05355207	Interventional study	1	Not yet recruiting
Tezepelumab	anti-TSLP	NCT04851964	Multicentre, randomized, double-blind, placebo-controlled, parallel-group study	3	Active, not recruiting
TQH2722	IL-4Rα	NCT06439381 NCT06089278	Multicenter, randomized, continuing trial	2	Recruiting
Telikibart (GR1802)	IL-4Rα	NCT05873803	Randomized, double-blind, placebo-controlled, multicenter study	2	Active, not recruiting
Telikibart (GR1802)	IL-4Rα	NCT06516302	Randomized, double-blind, placebo-controlled, multicenter study	3	Not yet recruiting
Mepolizumab	IL-5	NCT05923047	Randomized, controlled multicenter trial	4	Not yet recruiting
Mepolizumab	IL-5	NCT05598814	Randomized, two-arm trial	4	Recruiting
**Allergic rhinitis**					
Tezepelumab	TSLP	NCT06189742	Non-randomized, single-group assignment, open-label study	2	Active, recruiting
Stapokibart (CM310)	IL-4Rα	NCT06300203	Randomized, double-blind, placebo-controlled study	2	Not yet recruiting
Stapokibart (CM310)	IL-4Rα	NCT06525597	Randomized, double-blind, placebo-controlled study	2	Recruiting
Telikibart (GR1802)	IL-4Rα	NCT06315426	Randomized, double-blind, placebo-controlled, multicenter study	2	Not yet recruiting
Telikibart (GR1802)	IL-4Rα	NCT06028490	Randomized, double-blind, placebo-controlled study	2	Recruiting

## 3. Discussion

Biologic therapies have revolutionized the management of allergic diseases, offering targeted interventions for severe and refractory cases. However, ongoing research continues to expand the potential applications of biologics, addressing unmet needs and optimizing treatment efficacy. Future directions include novel therapeutic targets, precision medicine approaches, cost and accessibility improvements, and innovations in delivery mechanisms.

Emerging biologics represent a significant advancement over existing therapies, introducing novel mechanisms of action, improved formulations, and broader applications. While currently approved biologics, such as anti-IgE, anti-IL-5, and anti-IL-4Rα agents, primarily target T2 inflammation, emerging therapies focus on upstream or alternative pathways, addressing gaps in current treatment strategies. Investigational agents such as anti-IL-33, anti-TSLP, anti-IL-23, and anti-OX40/OX40L target upstream mediators of inflammation, modulating both T2 and non-T2 pathways to benefit broader disease endotypes, including those resistant to corticosteroids or existing biologics. Furthermore, several novel biologics exhibit dual or multifunctional targeting capabilities. For instance, bispecific monoclonal antibodies like IBI 3002 (targeting IL-4Rα and TSLP) [85] and lunsekimig (targeting TSLP and IL-13) [86] aim to address both T2 and non-T2 inflammation in asthma. Additionally, improved pharmacokinetics characterize many emerging therapies, enabling less frequent dosing. Depemokimab, for example, demonstrates an extended half-life compared to existing anti-IL-5/IL-5R agents, such as benralizumab, mepolizumab, or reslizumab, enhancing patient adherence and reducing therapy-related costs [45]. Ecleralimab (CSJ117), the first inhaled anti-TSLP antibody fragment, introduces an innovative delivery method that directly targets airway inflammation. This inhaled approach enhances treatment efficiency, reduces systemic adverse events, and facilitates home-based therapy [66]. Similarly, biologics targeting specific allergens, such as REGN1908/1909 (targeting Fel d 1, the cat allergen) [69] and REGN5713-5714-5715 (targeting Bet v 1, the birch pollen allergen) [213], prevent IgE-mediated allergic reactions and have demonstrated promise in clinical trials, improving allergen tolerance and symptom control. Calpurbatug (TRL1068), currently under investigation in the phase 1 trial (NCT05355207) for CRSwNP, represents a novel approach targeting biofilm-associated pathogens. By disrupting the biofilm that shields *S. aureus* and *P. aeruginosa*, TRL1068 is expected to enhance bacterial susceptibility to standard antibiotics, addressing key challenges in managing recalcitrant disease associated with biofilm formation [204]. Nemolizumab exemplifies innovation in addressing symptom-specific pathways, targeting IL-31R signaling to alleviate pruritus in atopic dermatitis. Its efficacy in reducing disease severity and itch and its potential as a supportive treatment highlights a novel therapeutic approach to allergic diseases [110]. Meanwhile, a new class of monoclonal antibodies targeting the OX40-OX40L axis, such as amlitelimab and rocatinlimab, focuses specifically on T-cell-mediated inflammation, a significant departure from therapies primarily targeting eosinophils, mast cells, or neutrophils. As the first biologics to directly target T cells in allergic diseases, these agents hold great promise and are currently undergoing Phase 2/3 trials [116]. Highlighting the innovation of emerging biologics, lirentelimab offers a unique approach by targeting Siglec-8, a protein exclusively expressed in human eosinophils and mast cells. This cell-specific mechanism may reduce treatment-emergent adverse events (TEAEs) while providing a novel therapeutic option for eosinophil- and mast cell-driven conditions like CSU and non-EoE-EGIDs [150]. Future trials, particularly head-to-head comparisons with established biologics such as dupilumab, omalizumab, and anti-IL-5/IL-5R agents, will be essential to confirm lirentelimab’s efficacy and safety relative to current therapies.

Discussing the efficacy, retention rate, and safety of emerging biologics in allergic diseases compared to existing therapies is challenging due to several reasons. Firstly, there is a lack of head-to-head comparison trials evaluating novel agents directly against older biologics targeting similar inflammatory pathways. Secondly, emerging biologics often target distinct molecular mechanisms, such as IL-31R, OX40-OX40L, Siglec-8, or specific allergens, whereas existing therapies predominantly focus on T2 inflammation. Moreover, many emerging agents are in early-phase clinical trials primarily assessing safety and efficacy, with direct comparisons typically reserved for phase 3 studies. Despite these limitations, existing literature offers several key insights. Depemokimab, an anti-IL5R monoclonal antibody, demonstrates potential advantages over current agents like benralizumab, mepolizumab, and reslizumab due to its improved pharmacokinetic profile, enabling six-month dosing intervals that may enhance adherence and reduce costs [43]. However, whether it outperforms existing biologics in efficacy, safety, and retention rates remains unclear and is currently under investigation in a phase 3 trial (NCT04718389). Itepekimab, an anti-IL-33 monoclonal antibody, has been compared with dupilumab in moderate-to-severe asthma. Results showed itepekimab was slightly inferior, with a higher percentage of patients experiencing loss of asthma control (22% vs. 19%) and slightly lower improvement in prebronchodilator FEV1 (0.14 L vs. 0.16 L). The incidence of adverse events was similar, though injection site reactions were less frequent with itepekimab (1% vs. 5%) [55]. In the phase 2b GENESIS study, bermekimab, an anti-IL-1α agent, was inferior to dupilumab in atopic dermatitis (AD), with only 16.7% of patients in both 350 mg and 700 mg bermekimab groups achieving EASI-75 at week 16, compared to 51.2% for dupilumab. Bermekimab showed no significant symptom improvements over placebo, while dupilumab demonstrated greater efficacy. Adverse events occurred in 66% of bermekimab-treated patients versus 50.8% in the dupilumab group, with three serious adverse events and five treatment discontinuations reported only in the bermekimab groups [99]. In recent years, several novel agents targeting IL-13 or IL-13R in AD have been investigated, though direct comparisons with existing therapies like tralokinumab or dupilumab are lacking. A matching-adjusted indirect analysis by Rand et al. compared lebrikizumab Q4W to dupilumab Q1W/Q2W, showing equivalent or higher long-term maintenance of IGA 0/1 at week 52 for lebrikizumab (RR 1.334, *p* = 0.035) and similar EASI75 rates (RR 0.937, *p* = 0.490). Adverse event rates were comparable (RR 1.052, *p* = 0.526). These findings suggest lebrikizumab may be superior to dupilumab due to its similar efficacy, safety, and less frequent dosing [214]. However, direct comparisons in head-to-head trials are needed. An indirect comparison in Japanese patients with moderate-to-severe AD showed lebrikizumab had higher response rates than tralokinumab for IGA 0/1, EASI75, and itch NRS-4 and outperformed dupilumab for IGA 0/1 and itch NRS-4. Lebrikizumab was also the only agent superior to placebo across all outcome measures [215]. In preclinical studies, stapokibart, a novel mAb targeting IL-4Rα, showed comparable or slightly higher inhibitory activity to dupilumab. It may also have a better safety profile, with lower rates of conjunctivitis, though direct comparisons are needed to confirm its advantages in efficiency and safety [216,217]. Similarly, early studies on rademikibart, another anti-IL-4Rα biologic, suggest potential superiority to dupilumab, warranting further investigation [218]. Recently, Ye et al. conducted a phase 1 study assessing YH35324, a novel long-acting IgETrap-Fc fusion protein, in atopic diseases, including AD. YH35324 showed a slightly better safety profile than omalizumab, with fewer TEAEs and longer suppression of serum-free IgE levels, particularly in patients with serum IgE > 700 IU/mL. While these results suggest YH35324 may outperform omalizumab, further investigation is needed due to the study’s early phase and small cohort size [121]. Ligelizumab, another anti-IgE mAb, shows potential to outperform omalizumab in CSU, with a model-based meta-analysis indicating better efficacy at 72 mg and 120 mg doses compared to 150 mg omalizumab, though safety data comparisons are lacking [147]. Head-to-head trials are necessary to confirm these findings. UB-221, another anti-IgE biologic with dual IgE neutralization and CD-23-mediated IgE synthesis downregulation, has shown complex biological activity in early CSU studies, warranting further investigation to determine its superiority to omalizumab and ligelizumab in clinical settings [148]. Additionally, CMAB007, a biosimilar to omalizumab, is currently being evaluated for efficacy and safety in CSU in an ongoing trial (NCT06365879). In CRSwNP, while benralizumab has shown promise in early trials, a recent network analysis found it to be less effective than dupilumab, mepolizumab, and omalizumab in reducing nasal congestion severity and SNOT-22 scores at 24 weeks. These findings emphasize the need for continued comparative studies to better define the role of emerging biologics in allergic disease management [21].

Biomarker research is revolutionizing biologic therapies for allergic diseases by enabling precise patient stratification and personalized treatment approaches. Established biomarkers such as blood eosinophil count (BEC), FeNO, and serum IgE levels are pivotal in guiding biologic selection, particularly for T2 inflammation-targeted therapies, optimizing treatment efficacy in conditions like asthma and atopic dermatitis (AD). Emerging biologics targeting IL-5, IL-13, IL-23, IL-31R, IL-33, TSLP, and Siglec-8 pathways necessitate the identification of novel biomarkers to further refine patient selection. For example, periostin and DPP-4 are being explored as potential predictors for IL-13-targeting biologics in asthma [89], while IL-33 and soluble IL-33R levels in sputum or serum may aid in selecting therapies that inhibit IL-33 signaling [91]. In AD, IL-13-related biomarkers such as periostin and DPP-4, along with tissue expression of IL-13/IL-13R, may serve as universal biomarkers for biologics targeting this axis [126]. Additionally, proteins like CCL18, CCL20, CCL22, CCL27, VEGF, and IL-1RA are being investigated as predictive markers for nemolizumab efficacy [128]. Subgroup analyses suggest tezepelumab may be more effective in patients with specific biomarker profiles, including DPP-4, periostin, CCL17, and IgE levels [123]. Emerging biomarkers for well-established therapies like dupilumab include CD25, IL-31, and IL-36β [125]. Despite significant advances, further research is needed to validate and incorporate these emerging biomarkers into clinical practice. Such efforts will ensure treatments are tailored to individual disease phenotypes and endotypes, enhancing therapeutic precision and improving patient outcomes.

Interindividual variability in biologic therapy responses significantly affects both treatment efficacy and safety and arises from patient-specific, disease-related, and biologic-specific factors. Genetic polymorphisms, age, sex, and body weight influence how patients metabolize and respond to biologics, while comorbidities like obesity or allergic diseases engage shared inflammatory pathways, altering therapeutic outcomes. For instance, SNPs in IL1RL1 (rs1420101) and IL4RA (rs8832) are linked to enhanced responses to asthma biologics, particularly anti-IL-5 drugs, independent of other clinical factors [219]. Similarly, dupilumab response in AD is associated with female sex, younger age, low BMI, and absence of allergic rhinitis [124]. Disease heterogeneity, particularly T2-high versus non-T2 phenotypes, further contributes to variability, with biomarkers such as BEC, FeNO, and IgE guiding therapy selection and response [220]. Biologic-specific properties, including receptor binding affinity, pharmacokinetics, and dosing frequency, influence adherence and therapeutic consistency. For example, longer half-life agents like depemokimab provide more stable responses compared to those requiring frequent dosing [45]. Additionally, molecular target properties, such as expression levels, turnover rates, and genetic polymorphisms, play a role; baseline IgE levels predict omalizumab response, while SNPs in the *IL6* gene (rs12083537, rs2228145) are predictive for tocilizumab efficacy. Even practical factors, such as the anatomical site of subcutaneous injection, can impact drug absorption and biological activity, underscoring the complex interplay of variables influencing patient outcomes [221]. These findings underscore the complexity of interindividual variability in biological therapy responses, highlighting the need for personalized approaches to optimize treatment efficacy and safety. Continued research into genetic, clinical, and biologic-specific factors will be crucial for refining patient selection and improving therapeutic outcomes.

Safety is a cornerstone in the evaluation of biological therapies for allergic diseases, encompassing both established and emerging agents. Clinical trials have consistently highlighted their favorable safety profiles, with variability depending on the target pathway and patient population. A recent safety analysis of FDA-approved biologics for allergic diseases, including asthma, AD, CRSwNP, and CSU, revealed good overall safety, with anaphylaxis rates ranging from 0–1% [23]. However, gaps remain, particularly in understanding the risks associated with biologics in pregnant or breastfeeding patients. Additionally, there is no evidence that biologics increase the risk of parasitic infections. In terms of infectious diseases, biologics like dupilumab, mepolizumab, and benralizumab present a low risk of herpes zoster infection, and no significant evidence links biologics to an increased malignancy risk. However, due to the relatively short duration of clinical trials, the potential long-term risk of malignancy cannot be entirely excluded [23]. Drug-specific adverse events (AEs) should be noted, such as omalizumab-related arthralgia, mepolizumab-associated alopecia, benralizumab-induced hepatitis, and cytokine-release hypersensitivity reactions. Dupilumab is associated with eosinophilia (4.1% vs. 0.6% with placebo) and conjunctivitis, which affects 3–23% of recipients. Similarly, conjunctivitis is reported in up to 10% of patients treated with tralokinumab [23]. Safety concerns also extend to emerging biologics. For instance, bermekimab treatment in AD was linked to higher rates of AEs compared to placebo and dupilumab (66%, 54.5%, and 50.8%, respectively) [99]. Nemolizumab, a promising agent for AD that targets pruritus and inflammatory pathways, has been associated with peripheral or facial edema (1–2%) and asthma exacerbations in 2–5% of treated patients [222]. In conclusion, while biological therapies for allergic diseases generally exhibit favorable safety profiles, drug-specific adverse events and gaps in long-term safety data underscore the need for continued monitoring and research. As emerging biologics expand therapeutic options, careful evaluation of their safety, especially in diverse and comorbid patient populations, will be essential to optimize clinical outcomes. Given the chronic nature of allergic diseases, long-term studies are essential to evaluate the safety and efficacy of biologics. Monitoring for potential adverse effects, such as increased infection risk or immune dysregulation, is critical. Post-marketing surveillance and real-world studies will provide invaluable insights into the long-term outcomes of biologics therapies [223].

The advent of biologics and JAK inhibitors (JAKi) has revolutionized the management of AD, particularly in patients with moderate-to-severe disease and allergic comorbidities such as asthma or CRSwNP. Insights from the Delphi consensus, a scientific agreement among Italian dermatologists, offer valuable guidance on the comparative roles of these therapies and their importance in optimizing treatment strategies for diverse patient populations [224]. According to these recommendations, JAKi is indicated as a first-line treatment for patients with severe involvement in difficult-to-treat areas, including the face, neck, hands, and genitalia. Additionally, JAKi are preferred in the itch-dominant phenotype of AD (itch-NRS ≥7) and in patients presenting with concomitant psoriasis or an AD-psoriasis overlap pattern. Notably, JAKi should also be considered over dupilumab in patients with a history of recurrent conjunctivitis [224]. Based on clinical trials and real-world data, JAKi demonstrates faster response times and higher efficacy compared to currently available biologics. However, biologics remain the preferred option for patients with atopic comorbidities such as conjunctivitis, asthma, or allergic rhinitis due to their established efficacy in these conditions. JAKi are a viable alternative for patients with atopic comorbidities who are refractory to or contraindicated for biologics. Ongoing trials evaluating JAKi, including inhalable dry powder formulations, in patients with AD and concomitant asthma may provide further insights into their role alongside biologics in modern AD treatment [224]. Future head-to-head studies comparing these therapies, particularly in patients with allergic comorbidities, will be essential to further delineate their respective roles.

Despite significant benefits and advancements of biologics in the current treatment of allergic diseases, it is pertinent to acknowledge the limitations inherent in biologic drug research. A notable constraint is the substantial proportion of early phase 1 and 2 trials to the total pool of studies on the discussed novel biologic drugs in this manuscript, which results from the stage of advancement in the efforts to obtain regulatory approval for the molecule for widespread use and time-consuming process associated with drug research. These studies place minimal emphasis on long-term efficacy and safety. Instead, they primarily focus on delineating the optimal dosage of the therapeutic agent and evaluating its short-term safety and efficacy. The initial phases of such research processes are distinguished by a limited number of participants and the enrollment of specific patient groups with narrowly defined inclusion criteria. Moreover, a major part of the discussed trials involves the comparison of the effectiveness of biologics with placebo, without the active comparator, whereas the number of studies comparing the efficacy of various biological drugs is lacking. The interpretation of the results of such studies is difficult, and their impact on current clinical standards is limited. In addition, the multicenter character of clinical trials may not fully reflect the efficacy and safety profile of certain biologics, given the interindividual variability between human populations.

Notwithstanding the exorbitant expenses associated with research and development, the financial burden of treating patients with biological drugs remains higher than conventional treatment modalities, even though biological drugs have been shown to improve patients’ QoL and reduce the need for medical services, such as hospitalizations or medical consultations. In their analysis, Padilla-Galo et al. demonstrated that benralizumab treatment for asthma patients in Spain costs approximately €2499 more than standard treatment (€14,043 vs. €11,544) but reduces severe exacerbations by 88% [225]. Similarly, a Canadian study reported that adding tezepelumab to standard care resulted in a quality-adjusted life-year (QALY) gain of 1.077 compared to standard care alone, with an incremental cost-utility ratio of CAD 192,357/QALY. Notably, tezepelumab yielded the highest incremental QALYs and the lowest incremental cost among biologics for asthma, indicating a cost-effective advantage [226]. Tugay et al., in a real-world analysis, found that while omalizumab significantly increased per-patient costs (23,607.08 vs. 425,329.81 Turkish Lira for standard care vs. omalizumab), its incremental cost-effectiveness ratio of 52,427.04 Turkish Lira/life year and cost-utility ratio of 122,675.57 Turkish Lira/QALY fell below the willingness-to-pay threshold of 156,948 Turkish Lira, supporting its socioeconomic viability [227]. The high cost of biologics primarily stems from the intrinsic cost of the drugs themselves. Anderson et al. estimated that biologics prices in the USA would need to decrease by at least 60% to achieve cost-effectiveness [228]. Future reductions in biologic therapy costs may rely on the development of biosimilars, optimization of manufacturing processes, and improved patient stratification through machine learning-based predictive models [22].

From a future perspective, digital health tools are expected to play a pivotal role in biological therapies. Remote monitoring of biomarkers like FeNO and adherence tracking through wearable devices can enhance patient management. Artificial intelligence (AI)-driven decision-support systems may assist clinicians in selecting the most suitable biological therapy based on patient-specific data. Digital platforms for patient-reported outcome measures (PROMs) will further improve the monitoring of treatment efficacy and satisfaction [229].

The future of biologic therapies in allergic diseases is poised for significant advancements, driven by innovative therapeutic targets, precision medicine approaches, and efforts to improve cost-effectiveness and accessibility. As ongoing research addresses the unmet needs of diverse patient populations, biologics are expected to transform the management of allergic diseases, offering hope for better outcomes and improved QoL for millions of patients worldwide.

## 4. Conclusions

Biologic therapies have revolutionized the management of severe allergic diseases by targeting key immune pathways, offering effective options for patients with refractory conditions. Among the emerging therapies, novel agents targeting alarmins, such as TSLP and IL-33, are particularly promising, as they modulate upstream inflammatory cascades, benefiting both type 2 and non-type 2 inflammation. In addition, innovative approaches like anti-Siglec-8 antibodies aim to address eosinophil- and mast cell-mediated diseases, while therapies targeting specific allergens, such as cat or birch tree allergens, offer precision-based solutions for allergen-driven conditions. Antibiofilm strategies targeting pathogen-protecting biofilms represent a breakthrough in combating treatment-resistant infections, particularly in CRSwNP. Furthermore, novel delivery systems, including inhalation formulations, are advancing biological therapy by improving patient compliance and drug efficacy, particularly for respiratory conditions such as asthma. While these innovations highlight biologics’ potential to address unmet allergic disease needs, challenges related to affordability, long-term safety, and equitable access remain significant. Ongoing research and policy advancements are essential to integrate these novel therapies into routine practice, ensuring sustainable and effective management for diverse patient populations.

## Data Availability

No new data were created or analyzed in this study. Data sharing is not applicable to this article.

## Supplementary Materials

The following supporting information can be downloaded at: https://www.mdpi.com/article/10.3390/jcm14041079/s1, Table S1: Summary of clinical trials investigating novel biologics in allergic diseases (2019–2024). Table S2: Summary of ongoing clinical trials investigating novel biologics in allergic diseases.

## References

[1] Shin Y.H., Hwang J., Kwon R., Lee S.W., Kim M.S., Shin Y.H., Hwang J., Kwon R., Lee S.W., GBD 2019 Allergic Disorders Collaborators (2023). Global, regional, and national burden of allergic disorders and their risk factors in 204 countries and territories, from 1990 to 2019: A systematic analysis for the Global Burden of Disease Study. Allergy.

[2] Wang J., Zhou Y., Zhang H., Hu L., Liu J., Wang L., Wang T., Zhang H., Cong L., Wang Q. (2023). Pathogenesis of allergic diseases and implications for therapeutic interventions. Signal Transduct. Target. Ther..

[3] Dierick B.J.H., van der Molen T., Flokstra-de Blok B.M.J., Muraro A., Postma M.J., Kocks J.W.H., van Boven J.F. (2020). Burden and socioeconomics of asthma, allergic rhinitis, atopic dermatitis and food allergy. Expert Rev. Pharmacoecon. Outcomes Res..

[4] Song P., Adeloye D., Salim H., Dos Santos J.P., Campbell H., Sheikh A., Rudan I. (2022). Global, regional, and national prevalence of asthma in 2019: A systematic analysis and modelling study. J. Glob. Health.

[5] Tian J., Zhang D., Yang Y., Huang Y., Wang L., Yao X., Lu Q. (2024). Global epidemiology of atopic dermatitis: A comprehensive systematic analysis and modelling study. Br. J. Dermatol..

[6] Rank M.A., Wonnaparhown A.M., Freeman C.M. (2023). Recent guidelines addressing chronic rhinosinusitis with nasal polyps: Practical aspects. Pol. Arch. Intern. Med..

[7] Raciborski F., Arcimowicz M., Samolinski B., Pinkas W., Samel-Kowalik P., Śliwczyński A. (2020). Recorded prevalence of nasal polyps increases with age. Adv. Dermatol. Allergol..

[8] Fricke J., Ávila G., Keller T., Weller K., Lau S., Maurer M., Zuberbier T., Keil T. (2020). Prevalence of chronic urticaria in children and adults across the globe: Systematic review with meta-analysis. Allergy.

[9] Papaiakovou G., Papageorgiou A., Bakakos A., Sinaniotis A.C., Rovina N. (2024). Eosinophilic gastrointestinal diseases: Current perspectives on pathogenesis and management. Explor. Asthma Allergy.

[10] Alnahas S., Abouammoh N., Althagafi W., Abd-Ellatif E.E. (2023). Prevalence, severity, and risk factors of allergic rhinitis among schoolchildren in Saudi Arabia: A national cross-sectional study, 2019. World Allergy Organ. J..

[11] Wong A., Tavakoli H., Sadatsafavi M., Carlsten C., FitzGerald J.M. (2017). Asthma control and productivity loss in those with work-related asthma: A population-based study. J. Asthma.

[12] Rönnebjerg L., Axelsson M., Kankaanranta H., Backman H., Rådinger M., Lundbäck B., Ekerljung L. (2021). Severe Asthma in a General Population Study: Prevalence and Clinical Characteristics. J. Asthma Allergy.

[13] Fuxench Z.C.C., Block J.K., Boguniewicz M., Boyle J., Fonacier L., Gelfand J.M., Grayson M.H., Margolis D.J., Mitchell L., Silverberg J.I. (2019). Atopic Dermatitis in America Study: A Cross-Sectional Study Examining the Prevalence and Disease Burden of Atopic Dermatitis in the US Adult Population. J. Investig. Dermatol..

[14] Soong W., Patil D., Pivneva I., Signorovitch J., Wells M.A., Balp M.-M., Kuruvilla M. (2023). Disease burden and predictors associated with non-response to antihistamine-based therapy in chronic spontaneous urticaria. World Allergy Organ. J..

[15] Savouré M., Bousquet J., Leynaert B., Renuy A., Siroux V., Goldberg M., Zins M., Jacquemin B., Nadif R. (2023). Rhinitis phenotypes and multimorbidities in the general population: The CONSTANCES cohort. Eur. Respir. J..

[16] Ramírez-Jiménez F., Pavón-Romero G.F., Velásquez-Rodríguez J.M., López-Garza M.I., Lazarini-Ruiz J.F., Gutiérrez-Quiroz K.V., Teran L.M. (2023). Biologic Therapies for Asthma and Allergic Disease: Past, Present, and Future. Pharmaceuticals.

[17] Kavanagh J.E., Hearn A.P., Jackson D.J. (2022). A pragmatic guide to choosing biologic therapies in severe asthma. Breathe.

[18] Dubini M., Benzecry V., Rivolta F., Sangalli A., Marzano A.V., Pravettoni V., Tavecchio S., Ferrucci S.M. (2023). Asthma improvement in patients treated with dupilumab for severe atopic dermatitis. Front. Allergy.

[19] Ratchataswan T., Banzon T.M., Thyssen J.P., Weidinger S., Guttman-Yassky E., Phipatanakul W. (2021). Biologics for Treatment of Atopic Dermatitis: Current Status and Future Prospect. J. Allergy Clin. Immunol. Pract..

[20] Kinoshita Y., Sanuki T. (2023). Review of Non-Eosinophilic Esophagitis-Eosinophilic Gastrointestinal Disease (Non-EoE-EGID) and a Case Series of Twenty-Eight Affected Patients. Biomolecules.

[21] Cai S., Xu S., Lou H., Zhang L. (2022). Comparison of Different Biologics for Treating Chronic Rhinosinusitis With Nasal Polyps: A Network Analysis. J. Allergy Clin. Immunol. Pract..

[22] Azzano P., Dufresne É., Poder T., Bégin P. (2021). Economic considerations on the usage of biologics in the allergy clinic. Allergy.

[23] Sitek A.N., Li J.T., Pongdee T. (2023). Risks and safety of biologics: A practical guide for allergists. World Allergy Organ. J..

[24] Runnstrom M., Pitner H., Xu J., Lee F.E.-H., Kuruvilla M. (2022). Utilizing Predictive Inflammatory Markers for Guiding the Use of Biologicals in Severe Asthma. J. Inflamm. Res..

[25] Usuba K., Zhang L., Liu X., Han T., Nightingale N., Tehrani A., Zhang S., Howarth P., Alfonso-Cristancho R. (2024). Predicting real-world response to mepolizumab in severe asthma using machine learning. Eur. Respir. J..

[26] Bonini S., Bonini M. (2017). Biosimilars and drug development in allergic and immunologic diseases. J. Allergy Clin. Immunol..

[27] Miller R.L., Grayson M.H., Strothman K. (2021). Advances in asthma: New understandings of asthma’s natural history, risk factors, underlying mechanisms, and clinical management. J. Allergy Clin. Immunol..

[28] Chung K.F., Dixey P., Abubakar-Waziri H., Bhavsar P., Patel P.H., Guo S., Ji Y. (2022). Characteristics, phenotypes, mechanisms and management of severe asthma. Chin. Med. J..

[29] D’Amato G., Vitale C., Molino A., Stanziola A., Sanduzzi A., Vatrella A., Mormile M., Lanza M., Calabrese G., Antonicelli L. (2016). Asthma-related deaths. Multidiscip. Respir. Med..

[30] Shah P.A., Brightling C. (2023). Biologics for severe asthma-Which, when and why?. Respirol. Carlton. Vic..

[31] Kuruvilla M.E., Lee F.E.-H., Lee G.B. (2019). Understanding Asthma Phenotypes, Endotypes, and Mechanisms of Disease. Clin. Rev. Allergy Immunol..

[32] Oppenheimer J., Hoyte F.C., Phipatanakul W., Silver J., Howarth P., Lugogo N.L. (2022). Allergic and eosinophilic asthma in the era of biomarkers and biologics: Similarities, differences and misconceptions. Ann. Allergy Asthma Immunol..

[33] Kyriakopoulos C., Gogali A., Bartziokas K., Kostikas K. (2021). Identification and treatment of T2-low asthma in the era of biologics. ERJ Open Res..

[34] Hudey S.N., Ledford D.K., Cardet J.C. (2020). Mechanisms of non-type 2 asthma. Curr. Opin. Immunol..

[35] Adrish M., Akuthota P. (2023). Approach to non-type 2 asthma. Respir. Med..

[36] Liu T., Woodruff P.G., Zhou X. (2024). Advances in non-type 2 severe asthma: From molecular insights to novel treatment strategies. Eur. Respir. J..

[37] GINA Difficult-to-Treat & Severe Asthma Guide. https://ginasthma.org/wp-content/uploads/2024/11/GINA-Severe-Asthma-Guide-2024-WEB-WMS.pdf.

[38] Loureiro C.C., Amaral L., Ferreira J.A., Lima R., Pardal C., Fernandes I., Semedo L., Arrobas A. (2018). Omalizumab for Severe Asthma: Beyond Allergic Asthma. BioMed Res. Int..

[39] Cushen B., Menzies-Gow A. (2020). Benralizumab: An updated treatment of eosinophilic asthma. Expert Rev. Respir. Med..

[40] Sardon-Prado O., Diaz-Garcia C., Corcuera-Elosegui P., Korta-Murua J., Valverde-Molina J., Sanchez-Solis M. (2023). Severe Asthma and Biological Therapies: Now and the Future. J. Clin. Med..

[41] Grey A., Katelaris C.H. (2019). Dupilumab in the Treatment of Asthma. Immunotherapy.

[42] Panettieri R., Lugogo N., Corren J., Ambrose C.S. (2024). Tezepelumab for Severe Asthma: One Drug Targeting Multiple Disease Pathways and Patient Types. J. Asthma. Allergy.

[43] Wechsler M.E., Jackson D.J., Bernstein D., Korn S., Pfeffer P.E., Chen R., Saito J., Martinez G.d.L., Dymek L., Jacques L. (2024). Twice-Yearly Depemokimab in Severe Asthma with an Eosinophilic Phenotype. N. Engl. J. Med..

[44] Seluk L., Davis A.E., Rhoads S., Wechsler M.E. (2024). Novel asthma treatments: Advancing beyond approved novel step-up therapies for asthma. Ann. Allergy Asthma Immunol..

[45] Singh D., Fuhr R., Bird N.P., Mole S., Hardes K., Man Y.L., Cahn A., Yancey S.W., Pouliquen I.J. (2022). A Phase 1 study of the long-acting anti-IL-5 monoclonal antibody GSK3511294 in patients with asthma. Br. J. Clin. Pharmacol..

[46] Antoniu S.A. (2016). Lebrikizumab for the treatment of asthma. Expert Opin. Investig. Drugs.

[47] Marone G., Granata F., Pucino V., Pecoraro A., Heffler E., Loffredo S., Scadding G.W., Varricchi G. (2019). The Intriguing Role of Interleukin 13 in the Pathophysiology of Asthma. Front. Pharmacol..

[48] Austin C.D., Gonzalez Edick M., Ferrando R.E., Solon M., Baca M., Mesh K., Bradding P., Gauvreau G.M., Sumino K., FitzGerald J.M. (2020). A randomized, placebo-controlled trial evaluating effects of lebrikizumab on airway eosinophilic inflammation and remodelling in uncontrolled asthma (CLAVIER). Clin. Exp. Allergy.

[49] Hanania N.A., Korenblat P., Chapman K.R., Bateman E.D., Kopecky P., Paggiaro P., Yokoyama A., Olsson J., Gray S., Holweg C.T.J. (2016). Efficacy and safety of lebrikizumab in patients with uncontrolled asthma (LAVOLTA I and LAVOLTA II): Replicate, phase 3, randomised, double-blind, placebo-controlled trials. Lancet Respir. Med..

[50] Szefler S., Roberts G., Rubin A., Zielen S., Kuna P., Alpan O., Anzures-Cabrera J., Chen Q., Holweg C., Kaminski J. (2022). Efficacy, safety, and tolerability of lebrikizumab in adolescent patients with uncontrolled asthma (ACOUSTICS). Clin. Transl. Allergy.

[51] Corren J., Szefler S.J., Sher E., Korenblat P., Soong W., Hanania N.A., Berman G., Brusselle G., Zitnik R., Natalie C.R. (2024). Lebrikizumab in Uncontrolled Asthma: Reanalysis in a Well-Defined Type 2 Population. J. Allergy Clin. Immunol. Pract..

[52] England E., Rees D.G., Scott I.C., Carmen S., Chan D.T.Y., Huntington C.E.C., Houslay K.F., Erngren T., Penney M., Majithiya J.B. (2023). Tozorakimab (MEDI3506): An anti-IL-33 antibody that inhibits IL-33 signalling via ST2 and RAGE/EGFR to reduce inflammation and epithelial dysfunction. Sci. Rep..

[53] Ding W., Zou G.-L., Zhang W., Lai X.-N., Chen H.-W., Xiong L.-X. (2018). Interleukin-33: Its Emerging Role in Allergic Diseases. Molecules.

[54] Corren J., Reid F., Moate R., Jimenez E., Sadiq M., Williams A., Rytelewski M., Muthas D., Brooks D., Lindqvist E. (2024). S90 FRONTIER-3: A randomized, phase 2a study to evaluate the efficacy and safety of tozorakimab (an anti-interleukin-33 monoclonal antibody) in early-onset asthma. Thorax.

[55] Wechsler M.E., Ruddy M.K., Pavord I.D., Israel E., Rabe K.F., Ford L.B., Maspero J.F., Abdulai R.M., Hu C.-C., Martincova R. (2021). Efficacy and Safety of Itepekimab in Patients with Moderate-to-Severe Asthma. N. Engl. J. Med..

[56] Kosloski M.P., Kalliolias G.D., Xu C.R., Harel S., Lai C.H., Zheng W., Davis J.D., Kamal M.A. (2022). Pharmacokinetics and pharmacodynamics of itepekimab in healthy adults and patients with asthma: Phase I first-in-human and first-in-patient trials. Clin. Transl. Sci..

[57] Akinseye C., Crim C., Newlands A., Fairman D. (2023). Efficacy and safety of GSK3772847 in participants with moderate-to-severe asthma with allergic fungal airway disease: A phase IIa randomized, multicenter, double-blind, sponsor-open, comparative trial. PLoS ONE.

[58] Nnane I., Frederick B., Yao Z., Raible D., Shu C., Badorrek P., Boer M.v.D., Branigan P., Duffy K., Baribaud F. (2020). The first-in-human study of CNTO 7160, an anti-interleukin-33 receptor monoclonal antibody, in healthy subjects and patients with asthma or atopic dermatitis. Br. J. Clin. Pharmacol..

[59] Crim C., Stone S., Millar V., Lettis S., Bel E.H., Menzies-Gow A., Chanez P., Wenzel S., Lugogo N., Bleecker E.R. (2022). IL-33 receptor inhibition in subjects with uncontrolled asthma: A randomized, placebo-controlled trial. J. Allergy Clin. Immunol. Glob..

[60] Bagnasco D., Testino E., Nicola S., Melissari L., Russo M., Canevari R.F., Brussino L., Passalacqua G. (2022). Specific Therapy for T2 Asthma. J. Pers. Med..

[61] Kelsen S.G., Agache I.O., Soong W., Israel E., Chupp G.L., Cheung D.S., Theess W., Yang X., Staton T.L., Choy D.F. (2021). Astegolimab (anti-ST2) efficacy and safety in adults with severe asthma: A randomized clinical trial. J. Allergy Clin. Immunol..

[62] McKeage K., Duggan S. (2019). Risankizumab: First Global Approval. Drugs.

[63] Li Y., Hua S. (2014). Mechanisms of pathogenesis in allergic asthma: Role of interleukin-23. Respirology.

[64] Wakashin H., Hirose K., Maezawa Y., Kagami S.-I., Suto A., Watanabe N., Saito Y., Hatano M., Tokuhisa T., Iwakura Y. (2008). IL-23 and Th17 Cells Enhance Th2-Cell–mediated Eosinophilic Airway Inflammation in Mice. Am. J. Respir. Crit. Care Med..

[65] Brightling C.E., Nair P., Cousins D.J., Louis R., Singh D. (2021). Risankizumab in Severe Asthma—A Phase 2a, Placebo-Controlled Trial. N. Engl. J. Med..

[66] Garg D., Que L.G., Ingram J.L. (2024). Effects of biological therapies on patients with Type-2 high asthma and comorbid obesity. Front. Pharmacol..

[67] Gauvreau G.M., Hohlfeld J.M., FitzGerald J.M., Boulet L.-P., Cockcroft D.W., Davis B.E., Korn S., Kornmann O., Leigh R., Mayers I. (2023). Inhaled anti-TSLP antibody fragment, ecleralimab, blocks responses to allergen in mild asthma. Eur. Respir. J..

[68] Kamal M.A., Dingman R., Wang C.Q., Lai C., Rajadhyaksha M., DeVeaux M., Orengo J.M., Radin A., Davis J.D. (2021). REGN1908-1909 monoclonal antibodies block Fel d 1 in cat allergic subjects: Translational pharmacokinetics and pharmacodynamics. Clin. Transl. Sci..

[69] de Blay F.J., Gherasim A., Domis N., Meier P., Shawki F., Wang C.Q., Orengo J.M., DeVeaux M., Ramesh D., Jalbert J.J. (2022). REGN1908/1909 prevented cat allergen-induced early asthmatic responses in an environmental exposure unit. J. Allergy Clin. Immunol..

[70] Rymut S., Yoshida K., Sukumaran S., Cai F., Sperinde G., Sverkos V., Banerjee P., Belloni P., Lin J. (2020). Local Airway Concentration of Anti-Tryptase Antibody (MTPS9579A) Predicts Extent of Tryptase Disruption. J. Allergy Clin. Immunol..

[71] Vitte J. (2015). Human mast cell tryptase in biology and medicine. Mol. Immunol..

[72] Rymut S.M., Sukumaran S., Sperinde G., Bremer M., Galanter J., Yoshida K., Smith J., Banerjee P., Sverkos V., Cai F. (2022). Dose-dependent inactivation of airway tryptase with a novel dissociating anti-tryptase antibody (MTPS9579A) in healthy participants: A randomized trial. Clin. Transl. Sci..

[73] Rymut S.M., Henderson L.M., Poon V., Staton T.L., Cai F., Sukumaran S., Rhee H., Owen R., Ramanujan S., Yoshida K. (2023). A mechanistic PK/PD model to enable dose selection of the potent anti-tryptase antibody (MTPS9579A) in patients with moderate-to-severe asthma. Clin. Transl. Sci..

[74] Rhee H., Henderson L.M., Bauer R.N., Wong K., Staton T.L., Choy D.F., Banerjee P., Poon V., Yoshida K., Chen C. (2024). Airway tryptase levels inform the lack of clinical efficacy of the tryptase inhibitor MTPS9579A in asthma. Allergy.

[75] Corren J., Castro M., O’Riordan T., Hanania N.A., Pavord I.D., Quirce S., Chipps B.E., Wenzel S.E., Thangavelu K., Rice M.S. (2020). Dupilumab Efficacy in Patients with Uncontrolled, Moderate-to-Severe Allergic Asthma. J. Allergy Clin. Immunol. Pract..

[76] Diver S., Khalfaoui L., Emson C., Wenzel S.E., Menzies-Gow A., Wechsler M.E., Johnston J., Molfino N., Parnes J.R., Megally A. (2021). Effect of tezepelumab on airway inflammatory cells, remodelling, and hyperresponsiveness in patients with moderate-to-severe uncontrolled asthma (CASCADE): A double-blind, randomised, placebo-controlled, phase 2 trial. Lancet Respir. Med..

[77] Corren J., Parnes J.R., Wang L., Mo M., Roseti S.L., Griffiths J.M., van der Merwe R. (2017). Tezepelumab in Adults with Uncontrolled Asthma. N. Engl. J. Med..

[78] Menzies-Gow A., Corren J., Bourdin A., Chupp G., Israel E., Wechsler M.E., Brightling C.E., Griffiths J.M., Hellqvist Å., Bowen K. (2021). Tezepelumab in Adults and Adolescents with Severe, Uncontrolled Asthma. N. Engl. J. Med..

[79] Corren J., Menzies-Gow A., Chupp G., Israel E., Korn S., Cook B., Ambrose C.S., Hellqvist Å., Roseti S.L., Molfino N.A. (2023). Efficacy of Tezepelumab in Severe, Uncontrolled Asthma: Pooled Analysis of the PATHWAY and NAVIGATOR Clinical Trials. Am. J. Respir. Crit. Care Med..

[80] Ricciardolo F.L.M., Carriero V., Bertolini F. (2021). Which Therapy for Non-Type(T)2/T2-Low Asthma. J. Pers. Med..

[81] Hernandez M.L., Mills K., Almond M., Todoric K., Aleman M.M., Zhang H., Zhou H., Peden D.B. (2015). IL-1 receptor antagonist reduces endotoxin-induced airway inflammation in healthy volunteers. J. Allergy Clin. Immunol..

[82] Zhao Y., Li J.-Y., Yang B., Ding Y.-F., Wu L.-M., Zhang L.-T., Wang J.-Y., Lu Q.-J., Zhang C.-L., Zhang F.-R. (2024). Long-Term Efficacy and Safety of Stapokibart in Adults with Moderate-to-Severe Atopic Dermatitis: An Open-Label Extension, Nonrandomized Clinical Trial. BioDrugs.

[83] Gauvreau G.M., Harris J.M., Boulet L.-P., Scheerens H., Fitzgerald J.M., Putnam W.S., Cockcroft D.W., Davis B.E., Leigh R., Zheng Y. (2014). Targeting membrane-expressed IgE B cell receptor with an antibody to the M1 prime epitope reduces IgE production. Sci. Transl. Med..

[84] Solrikitug Phase II Clinical Trial|ATS 2024. https://www.delveinsight.com/ats-conference/ats-2024/solrikitug-phase-ii-clinical-trial.

[85] Innovent Announces First Participant Dosed in a Phase I Study of IBI3002 (an Anti-IL-4Rα/TSLP Bispecific Antibody) in Australia. https://pharmashots.com/press-releases/innovent-announces-first-participant-dosed-in-a-phase-i-study-of-ibi3002-an-anti-il-4r%CE%B1tslp-bispecific-antibody-in-australia.

[86] Deiteren A., Bontinck L., Conickx G., Vigan M., Dervaux N., Gassiot M., Bas S., Suratt B., Staudinger H., Krupka E. (2024). A first-in-human, single and multiple dose study of lunsekimig, a novel anti-TSLP/anti-IL-13 NANOBODY® compound, in healthy volunteers. Clin. Transl. Sci..

[87] Sun B., Shen K., Zhao R., Li Y., Xiang M., Lin J. (2024). Precision medicine for severe asthma—Biological targeted therapy. Int. Immunopharmacol..

[88] Suzaki I., Hirano K., Maruyama Y., Kobayashi H., Rubin B.K. (2020). IL-13-induced periostin production from human bronchial epithelial cells and human nasal epithelial cells. World Allergy Organ. J..

[89] Panettieri R.A., Sjöbring U., Péterffy A., Wessman P., Bowen K., Piper E., Colice G., Brightling C.E. (2018). Tralokinumab for severe, uncontrolled asthma (STRATOS 1 and STRATOS 2): Two randomised, double-blind, placebo-controlled, phase 3 clinical trials. Lancet Respir. Med..

[90] Nair P., O’Byrne P.M. (2019). The interleukin-13 paradox in asthma: Effective biology, ineffective biologicals. Eur. Respir. J..

[91] Zhao R., Shi Y., Liu N., Li B. (2023). Elevated levels of interleukin-33 are associated with asthma: A meta-analysis. Immun. Inflamm. Dis..

[92] Morikubo H., Tojima R., Maeda T., Matsuoka K., Matsuura M., Miyoshi J., Tamura S., Hisamatsu T. (2024). Machine learning using clinical data at baseline predicts the medium-term efficacy of ustekinumab in patients with ulcerative colitis. Sci. Rep..

[93] Arrais M., Lulua O., Quifica F., Rosado-Pinto J., Gama J.M.R., Taborda-Barata L. (2019). Prevalence of asthma, allergic rhinitis and eczema in 6-7-year-old schoolchildren from Luanda, Angola. Allergol. Immunopathol. (Madr).

[94] Fishbein A.B., Silverberg J.I., Wilson E.J., Ong P.Y. (2019). Update on Atopic Dermatitis: Diagnosis, Severity Assessment, and Treatment Selection. J. Allergy Clin. Immunol. Pract..

[95] Hanifin J.M., Baghoomian W.B., Grinich E., Leshem Y.A.M., Jacobson M.B., Simpson E.L.M. (2022). The Eczema Area and Severity Index—A Practical Guide. Dermatitis.

[96] Davis D.M., Drucker A.M., Alikhan A., Bercovitch L., Cohen D.E., Darr J.M., Eichenfield L.F., Frazer-Green L., Paller A.S., Schwarzenberger K. (2024). Guidelines of care for the management of atopic dermatitis in adults with phototherapy and systemic therapies. J. Am. Acad. Dermatol..

[97] Dupilumab Label, FDA-Approved Drugs Database Drugs@FDA. https://www.accessdata.fda.gov/drugsatfda_docs/label/2024/761055s064lbl.pdf.

[98] Tralokinumab Label, FDA-Approved Drugs Database. https://www.accessdata.fda.gov/drugsatfda_docs/label/2023/761180s001lbl.pdf.

[99] Simpson E.L., Guttman-Yassky E., Pawlikowski J., Ghorayeb E.G., Ota T., Lebwohl M.G. (2024). Interleukin-1α inhibitor bermekimab in patients with atopic dermatitis: Randomized and nonrandomized studies. Arch. Dermatol. Res..

[100] Blauvelt A., Thyssen J.P., Guttman-Yassky E., Bieber T., Serra-Baldrich E., Simpson E., Rosmarin D., Elmaraghy H., Meskimen E., Natalie C.R. (2023). Efficacy and safety of lebrikizumab in moderate-to-severe atopic dermatitis: 52-week results of two randomized double-blinded placebo-controlled phase III trials. Br. J. Dermatol..

[101] Drucker A.M., Lam M., Prieto-Merino D., Malek R., Ellis A.G., Yiu Z.Z., Rochwerg B., Di Giorgio S., Arents B.W., Mohan T. (2024). Systemic Immunomodulatory Treatments for Atopic Dermatitis: Living Systematic Review and Network Meta-Analysis Update. JAMA Dermatol..

[102] Blauvelt A., Guttman-Yassky E., Lynde C., Khattri S., Schlessinger J., Imafuku S., Tada Y., Morita A., Wiseman M., Kwiek B. (2024). Cendakimab in Patients With Moderate to Severe Atopic Dermatitis: A Randomized Clinical Trial. JAMA Dermatol..

[103] Simpson E., Gooderham M., Kircik L., Armstrong A.W., Schlesinger T., Del Rosso J.Q., Murrell D.F., Thng S., Szepietowski J.C., Veverka K.A. (2024). 53652 Efficacy and safety of eblasakimab in adults with severe atopic dermatitis: A posthoc analysis of the Phase 2b TREK-AD trial. J. Am. Acad. Dermatol..

[104] Silverberg J.I., Strober B., Feinstein B., Xu J., Guttman-Yassky E., Simpson E.L., Li P., Longphre M., Song J., Guo J. (2023). Efficacy and safety of rademikibart (CBP-201), a next-generation mAb targeting IL-4Rα, in adults with moderate to severe atopic dermatitis: A phase 2 randomized trial (CBP-201-WW001). J. Allergy Clin. Immunol..

[105] Zhang J., Silverberg J.I., Guo J., Yun J., Pan W., Wei Z., Collazo R. (2024). 712-Positive 52-week maintenance data observed with rademikibart in patients with moderate-to-severe atopic dermatitis (SEASIDE CHINA). Br. J. Dermatol..

[106] Kosloski M.P., Guttman-Yassky E., Cork M.J., Worm M., Nahm D., Zhu X., Ruddy M.K., Harel S., Kamal M.A., Goulaouic H. (2024). Pharmacokinetics and pharmacodynamics of itepekimab in adults with moderate-to-severe atopic dermatitis: Results from two terminated phase II trials. Clin. Transl. Sci..

[107] Danto S.I., Tsamandouras N., Reddy P., Gilbert S., Mancuso J., Page K., Peeva E., Vincent M.S., Beebe J.S. (2024). Safety, Tolerability, Pharmacokinetics, and Pharmacodynamics of PF-06817024 in Healthy Participants, Participants with Chronic Rhinosinusitis with Nasal Polyps, and Participants with Atopic Dermatitis: A Phase 1, Randomized, Double-Blind, Placebo-Controlled Study. J. Clin. Pharmacol..

[108] Maurer M., Cheung D.S., Theess W., Yang X., Dolton M., Guttman A., Choy D.F., Dash A., Grimbaldeston M.A., Soong W. (2022). Phase 2 randomized clinical trial of astegolimab in patients with moderate to severe atopic dermatitis. J. Allergy Clin. Immunol..

[109] Kabashima K., Irie H. (2021). Interleukin-31 as a Clinical Target for Pruritus Treatment. Front. Med..

[110] Silverberg J.I., Wollenberg A., Reich A., Thaçi D., Legat F.J., Papp K.A., Gold L.S., Bouaziz J.D., Pink A.E., Carrascosa J.M. (2024). Nemolizumab with concomitant topical therapy in adolescents and adults with moderate-to-severe atopic dermatitis (ARCADIA 1 and ARCADIA 2): Results from two replicate, double-blind, randomised controlled phase 3 trials. Lancet.

[111] Liang J., Hu F., Dan M., Sang Y., Abulikemu K., Wang Q., Hong Y., Kang X. (2022). Safety and Efficacy of Nemolizumab for Atopic Dermatitis With Pruritus: A Systematic Review and Meta-Regression Analysis of Randomized Controlled Trials. Front. Immunol..

[112] Labib A., Does A.V., Yosipovitch G. (2022). Nemolizumab for atopic dermatitis. Drugs Today.

[113] Jackson D.J., Wechsler M.E., Brusselle G., Buhl R. (2024). Targeting the IL-5 pathway in eosinophilic asthma: A comparison of anti-IL-5 versus anti-IL-5 receptor agents. Allergy.

[114] Guttman-Yassky E., Bahadori L., Brooks L., Clark K.L., Grindebacke H., Ho C.N., Katial R., Pham T.H., Walton C., Datto C.J. (2023). Lack of effect of benralizumab on signs and symptoms of moderate-to-severe atopic dermatitis: Results from the phase 2 randomized, double-blind, placebo-controlled HILLIER trial. J. Eur. Acad. Dermatol. Venereol. JEADV..

[115] Bangert C., Loesche C., Skvara H., Fölster-Holst R., Lacour J.-P., Jones J., Burnett P., Novak N., Stingl G. (2023). IgE Depletion with Ligelizumab Does Not Significantly Improve Clinical Symptoms in Patients with Moderate-to-Severe Atopic Dermatitis. J. Investig. Dermatol..

[116] Croft M., Esfandiari E., Chong C., Hsu H., Kabashima K., Kricorian G., Warren R.B., Wollenberg A., Guttman-Yassky E. (2024). OX40 in the Pathogenesis of Atopic Dermatitis—A New Therapeutic Target. Am. J. Clin. Dermatol..

[117] Weidinger S., Bieber T., Cork M.J., Reich A., Wilson R., Quaratino S., Stebegg M., Brennan N., Gilbert S., O’malley J.T. (2023). Safety and efficacy of amlitelimab, a fully human nondepleting, noncytotoxic anti-OX40 ligand monoclonal antibody, in atopic dermatitis: Results of a phase IIa randomized placebo-controlled trial. Br. J. Dermatol..

[118] Guttman-Yassky E., Simpson E.L., Reich K., Kabashima K., Igawa K., Suzuki T., Mano H., Matsui T., Esfandiari E., Furue M. (2023). An anti-OX40 antibody to treat moderate-to-severe atopic dermatitis: A multicentre, double-blind, placebo-controlled phase 2b study. Lancet Lond. Engl..

[119] Fukaura R., Akiyama M. (2023). Targeting IL-36 in Inflammatory Skin Diseases. BioDrugs.

[120] Bissonnette R., Abramovits W., Saint-Cyr Proulx É., Lee P., Guttman-Yassky E., Zovko E., Sigmund R., Willcox J., Bieber T. (2023). Spesolimab, an anti-interleukin-36 receptor antibody, in patients with moderate-to-severe atopic dermatitis: Results from a multicentre, randomized, double-blind, placebo-controlled, phase IIa study. J. Eur. Acad. Dermatol. Venereol..

[121] Ye Y.M., Park J.W., Kim S.H., Cho Y.S., Lee S.Y., Lee S.Y., Sim S., Song E., Kim B., Lee J. (2024). Safety, Tolerability, Pharmacokinetics, and pharmacodynamics of YH35324, a novel Long-Acting High-Affinity IgETrap-Fc protein in subjects with Atopy: Results from the First-in-Human study. Int. Immunopharmacol..

[122] Albrecht M. (2021). Turning off the alarm—Targeting alarmins and other epithelial mediators of allergic inflammation with biologics. Allergologie.

[123] Simpson E.L., Parnes J.R., She D., Crouch S., Rees W., Mo M., van der Merwe R. (2019). Tezepelumab, an anti-thymic stromal lymphopoietin monoclonal antibody, in the treatment of moderate to severe atopic dermatitis: A randomized phase 2a clinical trial. J. Am. Acad. Dermatol..

[124] Chokevittaya P., Jirattikanwong N., Thongngarm T., Phinyo P., Wongsa C. (2024). Factors Associated With Dupilumab Response in Atopic Dermatitis: A Systematic Review and Meta-Analysis. J. Allergy Clin. Immunol. Pract..

[125] Wu Y., Gu C., Wang S., Yin H., Qiu Z., Luo Y., Li Z., Wang C., Yao X., Li W. (2023). Serum biomarker-based endotypes of atopic dermatitis in China and prediction for efficacy of dupilumab. Br. J. Dermatol..

[126] Wollenberg A., Howell M.D., Guttman-Yassky E., Silverberg J.I., Kell C., Ranade K., Moate R., van der Merwe R. (2019). Treatment of atopic dermatitis with tralokinumab, an anti–IL-13 mAb. J. Allergy Clin. Immunol..

[127] Maintz L., Welchowski T., Herrmann N., Brauer J., Traidl-Hoffmann C., Havenith R., Müller S., Rhyner C., Dreher A., CK-CARE Study Group (2023). IL-13, periostin and dipeptidyl-peptidase-4 reveal endotype-phenotype associations in atopic dermatitis. Allergy.

[128] Sidbury R., Alpizar S., Laquer V., Dhawan S., Abramovits W., Loprete L., Krishnaswamy J.K., Ahmad F., Jabbar-Lopez Z., Piketty C. (2022). Pharmacokinetics, Safety, Efficacy, and Biomarker Profiles During Nemolizumab Treatment of Atopic Dermatitis in Adolescents. Dermatol. Ther..

[129] Kolkhir P., Muñoz M., Asero R., Ferrer M., Kocatürk E., Metz M., Xiang Y.-K., Maurer M. (2022). Autoimmune chronic spontaneous urticaria. J. Allergy Clin. Immunol..

[130] Peck G., Hashim M., Shaughnessy C., Muddasani S., Elsayed N., Fleischer A. (2021). Global Epidemiology of Urticaria: Increasing Burden among Children, Females and Low-income Regions. Acta Dermato-Venereologica.

[131] Abdel-Meguid A.M., Awad S.M., Noaman M., Abdel Gawad A.M., Abou-Taleb D.A.E. (2024). Does chronic urticaria affect quality of sleep and quality of life?. J. Public Health Res..

[132] Guillén-Aguinaga S., Presa I.J., Aguinaga-Ontoso E., Guillén-Grima F., Ferrer M. (2016). Updosing nonsedating antihistamines in patients with chronic spontaneous urticaria: A systematic review and meta-analysis. Br. J. Dermatol..

[133] Zuberbier T., Abdul Latiff A.H., Abuzakouk M., Aquilina S., Asero R., Baker D., Ballmer-Weber B., Bangert C., Ben-Shoshan M., Bernstein J.A. (2022). The international EAACI/GA2LEN/EuroGuiDerm/APAAACI guideline for the definition, classification, diagnosis, and management of urticaria. Allergy.

[134] Xu L., Yu H., Xu S., Wang Y., Cao Y. (2024). Comparative efficacy and safety of the treatment by Omalizumab for chronic idiopathic urticaria in the general population: A systematic review and network meta-analysis. Ski. Res. Technol..

[135] Tharp M.D., Bernstein J.A., Kavati A., Ortiz B., MacDonald K., Denhaerynck K., Abraham I., Lee C.S. (2019). Benefits and Harms of Omalizumab Treatment in Adolescent and Adult Patients With Chronic Idiopathic (Spontaneous) Urticaria: A Meta-analysis of “Real-world” Evidence. JAMA Dermatol..

[136] Agache I., Rocha C., Pereira A., Song Y., Alonso-Coello P., Solà I., Beltran J., Posso M., Akdis C.A., Akdis M. (2021). Efficacy and safety of treatment with omalizumab for chronic spontaneous urticaria: A systematic review for the EAACI Biologicals Guidelines. Allergy.

[137] Keller L., Perera E.K., Bindon B., Khatiwada A., Stitt J.M., Dreskin S.C. (2024). Total IgE as a biomarker of omalizumab response in chronic spontaneous urticaria: A meta-analysis. Allergy Asthma Proc..

[138] Paton D.M. (2017). Dupilumab: Human monoclonal antibody against IL-4Rα for moderate to severe atopic dermatitis. Drugs Today.

[139] Maurer M., Casale T.B., Saini S.S., Ben-Shoshan M., Giménez-Arnau A.M., Bernstein J.A., Yagami A., Stjepanovic A., Radin A., Staudinger H.W. (2024). Dupilumab in patients with chronic spontaneous urticaria (LIBERTY-CSU CUPID): Two randomized, double-blind, placebo-controlled, phase 3 trials. J. Allergy Clin. Immunol..

[140] Maurer M., Casale T.B., Saini S.S., Ben-Shoshan M., Laws E., Maloney J., Bauer D., Radin A., Makhija M. (2024). Dupilumab Reduces Urticaria Activity, Itch, and Hives in Patients with Chronic Spontaneous Urticaria Regardless of Baseline Serum Immunoglobulin E Levels. Dermatol. Ther..

[141] Benralizumab Label, FDA-Approved Drugs Database Drugs@FDA. https://www.accessdata.fda.gov/drugsatfda_docs/label/2024/761070s021lbl.pdf.

[142] Altrichter S., Giménez-Arnau A.M., Bernstein J.A., Metz M., Bahadori L., Bergquist M., Brooks L., Ho C.N., Jain P., Lukka P.B. (2024). Benralizumab does not elicit therapeutic effect in patients with chronic spontaneous urticaria: Results from the phase IIb multinational randomized double-blind placebo-controlled ARROYO trial. Br. J. Dermatol..

[143] An S.B., Yang B.-G., Jang G., Kim D.-Y., Kim J., Oh S.-M., Oh N., Lee S., Moon J.-Y., Kim J.-A. (2022). Combined IgE neutralization and Bifidobacterium longum supplementation reduces the allergic response in models of food allergy. Nat. Commun..

[144] Gasser P., Tarchevskaya S.S., Guntern P., Brigger D., Ruppli R., Zbären N., Kleinboelting S., Heusser C., Jardetzky T.S., Eggel A. (2020). The mechanistic and functional profile of the therapeutic anti-IgE antibody ligelizumab differs from omalizumab. Nat. Commun..

[145] Maurer M., Giménez-Arnau A.M., Sussman G., Metz M., Baker D.R., Bauer A., Bernstein J.A., Brehler R., Chu C.-Y., Chung W.-H. (2019). Ligelizumab for Chronic Spontaneous Urticaria. N. Engl. J. Med..

[146] Ensina L.F., Gimenez-Arnau A.M., Sussman G., Hide M., Grattan C., Fomina D., Rigopoulos D., Berard F., Canonica G.W., Rockmann H. (2024). Efficacy and safety of ligelizumab in adults and adolescents with chronic spontaneous urticaria: Results of two phase 3 randomised controlled trials. Lancet.

[147] Zhao A., Zhang K., Wang Z., Ye K., Xu Z., Gong X., Zhu G. (2024). Time-course and dose-effect of omalizumab in treating chronic idiopathic urticaria/chronic spontaneous urticaria. Eur. J. Clin. Pharmacol..

[148] Kuo B.-S., Li C.-H., Chen J.-B., Shiung Y.-Y., Chu C.-Y., Lee C.-H., Liu Y.-J., Kuo J.-H., Hsu C., Su H.-W. (2022). IgE-neutralizing UB-221 mAb, distinct from omalizumab and ligelizumab, exhibits CD23-mediated IgE downregulation and relieves urticaria symptoms. J. Clin. Investig..

[149] Zhou B., Lin B., Li J., Qian W., Hou S., Zhang D., Kou G., Li B., Wang H., Chen Y. (2012). Tolerability, pharmacokinetics and pharmacodynamics of CMAB007, a humanized anti-immunoglobulin E monoclonal antibody, in healthy Chinese subjects. mAbs.

[150] Youngblood B.A., Leung J., Falahati R., Williams J., Schanin J., Brock E.C., Singh B., Chang A.T., O’sullivan J.A., Schleimer R.P. (2020). Discovery, Function, and Therapeutic Targeting of Siglec-8. Cells.

[151] Altrichter S., Staubach P., Pasha M., Singh B., Chang A.T., Bernstein J.A., Rasmussen H.S., Siebenhaar F., Maurer M. (2022). An open-label, proof-of-concept study of lirentelimab for antihistamine-resistant chronic spontaneous and inducible urticaria. J. Allergy Clin. Immunol..

[152] Canakinumab Label, FDA-Approved Drugs Database Drugs@FDA. https://www.accessdata.fda.gov/drugsatfda_docs/label/2024/125319s110lbl.pdf.

[153] Maul J.-T., Distler M., Kolios A., Maul L.V., Guillet C., Graf N., Imhof L., Lang C., Navarini A.A., Schmid-Grendelmeier P. (2021). Canakinumab Lacks Efficacy in Treating Adult Patients with Moderate to Severe Chronic Spontaneous Urticaria in a Phase II Randomized Double-Blind Placebo-Controlled Single-Center Study. J. Allergy Clin. Immunol. Pract..

[154] Redd W.D., Dellon E.S. (2022). Eosinophilic Gastrointestinal Diseases Beyond the Esophagus: An Evolving Field and Nomenclature. Gastroenterol. Hepatol..

[155] Dellon E.S., Gonsalves N., Abonia J.P., Alexander J.A., Arva N.C., Atkins D., Attwood S.E., Auth M.K., Bailey D.D., Biederman L. (2022). International Consensus Recommendations for Eosinophilic Gastrointestinal Disease Nomenclature. Clin. Gastroenterol. Hepatol..

[156] Papadopoulou A., Amil-Dias J., Auth M.K., Chehade M., Collins M.H., Gupta S.K., Gutiérrez-Junquera C., Orel R., Vieira M.C., Zevit N. (2024). Joint ESPGHAN/NASPGHAN Guidelines on Childhood Eosinophilic Gastrointestinal Disorders Beyond Eosinophilic Esophagitis. J. Pediatr. Gastroenterol. Nutr..

[157] Rothenberg M.E., Hottinger S.K., Gonsalves N., Furuta G.T., Collins M.H., Talley N.J., Peterson K., Menard-Katcher C., Smith M., Hirano I. (2022). Impressions and aspirations from the FDA GREAT VI Workshop on Eosinophilic Gastrointestinal Disorders Beyond Eosinophilic Esophagitis and Perspectives for Progress in the Field. J. Allergy Clin. Immunol..

[158] Marasco G., Visaggi P., Vassallo M., Fiocca M., Cremon C., Barbaro M.R., De Bortoli N., Bellini M., Stanghellini V., Savarino E.V. (2023). Current and Novel Therapies for Eosinophilic Gastrointestinal Diseases. Int. J. Mol. Sci..

[159] Sandström T. (2009). Omalizumab in the management of patients with allergic (IgE-mediated) asthma. J. Asthma Allergy.

[160] Losa F., Cingolani A. (2023). Eosinophilic gastrointestinal disorders: New perspectives and the emerging role of biological therapies. Explor. Asthma Allergy.

[161] Egan M., Furuta G.T. (2018). Eosinophilic gastrointestinal diseases beyond eosinophilic esophagitis. Ann. Allergy Asthma Immunol..

[162] Du Z., Wang Z., Zhou W., Yin J., Zhi Y. (2024). Eosinophilic gastritis and gluten-sensitive enteropathy manifested as hypoproteinemia and treated with omalizumab: A case report. Allergy Asthma Clin. Immunol..

[163] Lee L.-Y., Hew G.S.Y., Mehta M., Shukla S.D., Satija S., Khurana N., Anand K., Dureja H., Singh S.K., Mishra V. (2021). Targeting eosinophils in respiratory diseases: Biological axis, emerging therapeutics and treatment modalities. Life Sci..

[164] Prussin C., James S., Huber M., Klion A., Metcalfe D. (2003). Pilot study of anti-IL-5 in eosinophilic gastroenteritis. J. Allergy Clin. Immunol..

[165] Kim Y.-J., Prussin C., Martin B., Law M.A., Haverty T.P., Nutman T.B., Klion A.D. (2004). Rebound eosinophilia after treatment of hypereosinophilic syndrome and eosinophilic gastroenteritis with monoclonal anti–IL-5 antibody SCH55700. J. Allergy Clin. Immunol..

[166] Pitlick M.M., Li J.T., Pongdee T. (2022). Current and emerging biologic therapies targeting eosinophilic disorders. World Allergy Organ. J..

[167] Dellon E.S., Peterson K.A., Murray J.A., Falk G.W., Gonsalves N., Chehade M., Genta R.M., Leung J., Khoury P., Klion A.D. (2020). Anti–Siglec-8 Antibody for Eosinophilic Gastritis and Duodenitis. N. Engl. J. Med..

[168] Menzella F., Ruggiero P., Ghidoni G., Fontana M., Bagnasco D., Livrieri F., Scelfo C., Facciolongo N. (2020). Anti-IL5 Therapies for Severe Eosinophilic Asthma: Literature Review and Practical Insights. J. Asthma Allergy.

[169] Kuang F.L., Legrand F., Makiya M., Ware J., Wetzler L., Brown T., Magee T., Piligian B., Yoon P., Ellis J.H. (2019). Benralizumab for *PDGFRA*-Negative Hypereosinophilic Syndrome. N. Engl. J. Med..

[170] Kliewer K.L., Murray-Petzold C., Collins M.H., Abonia J.P., Bolton S.M., DiTommaso L.A., Martin L.J., Zhang X., Mukkada V.A., Putnam P.E. (2023). Benralizumab for eosinophilic gastritis: A single-site, randomised, double-blind, placebo-controlled, phase 2 trial. Lancet Gastroenterol. Hepatol..

[171] Thomas B., Mathur N. (2023). S4185 Beyond the Esophagus: Dupilumab’s Potential in Treatment of Eosinophilic Gastritis. Am. J. Gastroenterol..

[172] Sia T., Bacchus L., Tanaka R., Khuda R., Mallik S., Leung J. (2024). Dupilumab Can Induce Remission of Eosinophilic Gastritis and Duodenitis: A Retrospective Case Series. Clin. Transl. Gastroenterol..

[173] Ito K., Shibuya T., Nomura K., Haraikawa M., Kurosawa T., Haga K., Akazawa Y., Murakami T., Nomura O., Hojo M. (2023). Successful Treatment of Steroid-resistant Eosinophilic Gastrointestinal Disease with Mepolizumab. Intern. Med..

[174] Debnath P., Rathi P.M. (2021). Vedolizumab in Inflammatory Bowel Disease: West versus East. Inflamm. Intest. Dis..

[175] Grandinetti T., Biedermann L., Bussmann C., Straumann A., Hruz P. (2019). Eosinophilic Gastroenteritis: Clinical Manifestation, Natural Course, and Evaluation of Treatment with Corticosteroids and Vedolizumab. Dig. Dis. Sci..

[176] Janssens J., Vanuytsel T. (2023). Non-esophageal eosinophilic gastrointestinal diseases: A narrative review. Acta Gastro Enterol. Belg..

[177] Kim H.P., Reed C.C., Herfarth H.H., Dellon E.S. (2018). Vedolizumab Treatment May Reduce Steroid Burden and Improve Histology in Patients With Eosinophilic Gastroenteritis. Clin. Gastroenterol. Hepatol..

[178] Laidlaw T.M., Buchheit K.M. (2020). Biologics in chronic rhinosinusitis with nasal polyposis. Ann. Allergy Asthma Immunol..

[179] Stevens W.W., Schleimer R.P., Kern R.C. (2016). Chronic Rhinosinusitis with Nasal Polyps. J. Allergy Clin. Immunol. Pract..

[180] Sedaghat A.R., Kuan E.C., Scadding G.K. (2022). Epidemiology of Chronic Rhinosinusitis: Prevalence and Risk Factors. J. Allergy Clin. Immunol. Pract..

[181] Rank M.A., Chu D.K., Bognanni A., Oykhman P., Bernstein J.A., Ellis A.K., Golden D.B., Greenhawt M., Horner C.C., Ledford D.K. (2023). The Joint Task Force on Practice Parameters GRADE Guidelines for the Medical Management of Chronic Rhinosinusitis with Nasal Polyposis. J. Allergy Clin. Immunol..

[182] Hellings P., Fokkens W., Orlandi R., Adriaensen G., Alobid I., Baroody F., Bjermer L., Senior B., Cervin A., Cohen N. (2022). The EUFOREA pocket guide for chronic rhinosinusitis. Rhinology.

[183] Masson E. Comparative Efficacy and Safety of Monoclonal Antibodies and Aspirin Desensitization for Chronic Rhinosinusitis with Nasal Polyposis: A Systematic Review and Network Meta-Analysis. https://www.em-consulte.com/article/1509938/comparative-efficacy-and-safety-of-monoclonal-anti.

[184] Fokkens W.J., Viskens A.-S., Backer V., Conti D., De Corso E., Gevaert P., Scadding G.K., Wagemann M., Bernal-Sprekelsen M., Chaker A. (2023). EPOS/EUFOREA update on indication and evaluation of Biologics in Chronic Rhinosinusitis with Nasal Polyps 2023. Rhinology.

[185] Li T., Yin J., Yang Y., Wang G., Zhang Y., Song X. (2023). Dupilumab in chronic rhinosinusitis with nasal polyposis: Current status, challenges, and future perspectives. Expert Rev. Clin. Immunol..

[186] De Corso E., Pasquini E., Trimarchi M., La Mantia I., Pagella F., Ottaviano G., Garzaro M., Pipolo C., Torretta S., Seccia V. (2023). Dupilumab in the treatment of severe uncontrolled chronic rhinosinusitis with nasal polyps (CRSwNP): A multicentric observational Phase IV real-life study (DUPIREAL). Allergy.

[187] Geng B., Bachert C., Busse W.W., Gevaert P., Lee S.E., Niederman M.S., Chen Z., Lu X., Khokhar F.A., Kapoor U. (2021). Respiratory Infections and Anti-Infective Medication Use From Phase 3 Dupilumab Respiratory Studies. J. Allergy Clin. Immunol. Pract..

[188] Hopkins C., Wagenmann M., Bachert C., Desrosiers M., Han J.K., Hellings P.W., Lee S.E., Msihid J., Radwan A., Rowe P. (2021). Efficacy of dupilumab in patients with a history of prior sinus surgery for chronic rhinosinusitis with nasal polyps. Int. Forum Allergy Rhinol..

[189] Papacharalampous G.X., Constantinidis J., Fotiadis G., Zhang N., Bachert C., Katotomichelakis M. (2024). Chronic rhinosinusitis with nasal polyps (CRSwNP) treated with omalizumab, dupilumab, or mepolizumab: A systematic review of the current knowledge towards an attempt to compare agents’ efficacy. Int. Forum Allergy Rhinol..

[190] De Prado Gomez L., Khan Mbbs A.H., Peters A.T., Bachert C., Wagenmann M., Heffler E., Hopkins C., Hellings P.W., Zhang M., Xing J. (2022). Efficacy and Safety of Dupilumab Versus Omalizumab in Chronic Rhinosinusitis With Nasal Polyps and Asthma: EVEREST Trial Design. Am. J. Rhinol. Allergy.

[191] Namazy J.A., Blais L., Andrews E.B., Scheuerle A.E., Cabana M.D., Thorp J.M., Umetsu D.T., Veith J.H., Sun D., Kaufman D.G. (2020). Pregnancy outcomes in the omalizumab pregnancy registry and a disease-matched comparator cohort. J. Allergy Clin. Immunol..

[192] Klimek L., Förster-Ruhrmann U., Olze H., Beule A.G., Chaker A.M., Hagemann J., Huppertz T., Hoffmann T.K., Dazert S., Deitmer T. (2024). Monitoring mepolizumab treatment in chronic rhinosinusitis with nasal polyps (CRSwNP): Discontinue, change, continue therapy?. Allergol. Sel..

[193] Mullol J., Lund V.J., Wagenmann M., Han J.K., Sousa A.N., Smith S.G., Mayer B., Chan R.H., Fokkens W.J. (2024). Mepolizumab improves sense of smell in severe chronic rhinosinusitis with nasal polyps: SYNAPSE. Rhinology.

[194] Fujieda F., Wang C., Yoshikawa M., Asako M., Suzaki I., Bachert C., Han J.K., Fuller A., Baylis L., Su L. (2024). Mepolizumab in CRSwNP/ECRS and NP: The phase III randomised MERIT trial in Japan, China, and Russia. Rhinology.

[195] Chupp G., Alobid I., Lugogo N., Kariyawasam H., Bourdin A., Chaker A., Sousa A., Martin N., Yang S., Mayer B. (2023). Mepolizumab Reduces Systemic Corticosteroid Use in Chronic Rhinosinusitis With Nasal Polyps. J. Allergy Clin. Immunol. Pract..

[196] Fokkens W.J., Mullol J., Kennedy D., Philpott C., Seccia V., Kern R.C., Coste A., Sousa A.R., Howarth P.H., Benson V.S. (2023). Mepolizumab for chronic rhinosinusitis with nasal polyps (SYNAPSE): In-depth sinus surgery analysis. Allergy.

[197] Desrosiers M., Diamant Z., Castelnuovo P., Hellings P.W., Han J.K., Peters A.T., Silver J., Smith S.G., Fuller A., Sousa A.R. (2024). Sustained efficacy of mepolizumab in patients with severe chronic rhinosinusitis with nasal polyps: SYNAPSE 24-week treatment-free follow-up. Int. Forum Allergy Rhinol..

[198] Emson C., Han J.K., Hopkins C., Asimus S., Cann J.A., Chain D., Wu Y., Reddy Y., McCrae C., Cohen D. (2024). Pharmacokinetics/pharmacodynamics of benralizumab in chronic rhinosinusitis with nasal polyps: Phase III, randomized, placebo-controlled OSTRO trial. Br. J. Clin. Pharmacol..

[199] Bachert C., Han J.K., Desrosiers M.Y., Gevaert P., Heffler E., Hopkins C., Tversky J.R., Barker P., Cohen D., Emson C. (2022). Efficacy and Safety of Benralizumab in Chronic Rhinosinusitis with Nasal Polyps: A Randomized, Placebo-controlled Trial. J. Allergy Clin. Immunol..

[200] Takabayashi T., Asaka D., Okamoto Y., Himi T., Haruna S., Yoshida N., Kondo K., Yoshikawa M., Sakuma Y., Shibata K. (2021). A Phase II, Multicenter, Randomized, Placebo-Controlled Study of Benralizumab, a Humanized Anti-IL-5R Alpha Monoclonal Antibody, in Patients With Eosinophilic Chronic Rhinosinusitis. Am. J. Rhinol. Allergy.

[201] Canonica G.W., Harrison T.W., Chanez P., Menzella F., Louis R., Cosio B.G., Lugogo N.L., Mohan A., Burden A., Gil E.G. (2022). Benralizumab improves symptoms of patients with severe, eosinophilic asthma with a diagnosis of nasal polyposis. Allergy.

[202] Parnes J.R., Molfino N.A., Colice G., Martin U., Corren J., Menzies-Gow A. (2022). Targeting TSLP in Asthma. J. Asthma Allergy.

[203] Xiong Y.Q., Estellés A., Li L., Abdelhady W., Gonzales R., Bayer A.S., Tenorio E., Leighton A., Ryser S., Kauvar L.M. (2017). A Human Biofilm-Disrupting Monoclonal Antibody Potentiates Antibiotic Efficacy in Rodent Models of both Staphylococcus aureus and Acinetobacter baumannii Infections. Antimicrob. Agents Chemother..

[204] Conway J., Delanois R.E., Mont M.A., Stavrakis A., McPherson E., Stolarski E., Incavo S., Oakes D., Salvagno R., Adams J.S. (2024). Phase 1 study of the pharmacokinetics and clinical proof-of-concept activity of a biofilm-disrupting human monoclonal antibody in patients with chronic prosthetic joint infection of the knee or hip. Antimicrob. Agents Chemother..

[205] Siddiqui Z., Walker A., Pirwani M., Tahiri M., Syed I. (2022). Allergic rhinitis: Diagnosis and management. Br. J. Hosp. Med..

[206] Yang S.I., Lee I.H., Kim M., Ryu G., Kang S.Y., Kim M.A., Lee S.M., Kim H.J., Park D.Y., Lee Y.J. (2023). KAAACI Allergic Rhinitis Guidelines: Part 1. Update in Pharmacotherapy. Allergy Asthma Immunol. Res..

[207] Tidke M., Borghare P.T., Pardhekar P., Nasre Y., Gomase K., Chaudhary M. (2024). Recent Advances in Allergic Rhinitis: A Narrative Review. Cureus.

[208] Tsabouri S., Ntritsos G., Koskeridis F., Evangelou E., Olsson P., Kostikas K. (2021). Omalizumab for the treatment of allergic rhinitis: A systematic review and meta-analysis. Rhinology.

[209] Peters A.T., Wagenmann M., Bernstein J.A., Khan A.H., Nash S., Jacob-Nara J.A., Siddiqui S., Rowe P.J., Deniz Y. (2023). Dupilumab efficacy in patients with chronic rhinosinusitis with nasal polyps with and without allergic rhinitis. Allergy Asthma Proc..

[210] Corren J., Larson D., Altman M.C., Segnitz R.M., Avila P.C., Greenberger P.A., Baroody F., Moss M.H., Nelson H., Burbank A.J. (2022). Effects of combination treatment with tezepelumab and allergen immunotherapy on nasal responses to allergen: A randomized controlled trial. J. Allergy Clin. Immunol..

[211] Shamji M.H., Singh I., Layhadi J.A., Ito C., Karamani A., Kouser L., Sharif H., Tang J., Handijiev S., Parkin R.V. (2021). Passive Prophylactic Administration with a Single Dose of Anti-Fel d 1 Monoclonal Antibodies REGN1908-1909 in Cat Allergen-induced Allergic Rhinitis: A Randomized, Double-Blind, Placebo-controlled Clinical Trial. Am. J. Respir Crit. Care Med..

[212] Zhang Y., Yan B., Zhu Z., Wang X., Song X., Zhu D., Ma T., Zhang Y., Meng C., Wang G. (2024). Efficacy and safety of stapokibart (CM310) in uncontrolled seasonal allergic rhinitis (MERAK): An investigator-initiated, placebo-controlled, randomised, double-blind, phase 2 trial. eClinicalMedicine.

[213] Atanasio A., Franklin M.C., Kamat V., Hernandez A.R., Badithe A., Ben L.-H., Jones J., Bautista J., Yancopoulos G.D., Olson W. (2022). Targeting immunodominant Bet v 1 epitopes with monoclonal antibodies prevents the birch allergic response. J. Allergy Clin. Immunol..

[214] Rand K., Ramos-Goñi J.M., Akmaz B., Solé-Feu L., Armario-Hita J.-C. (2023). Matching-Adjusted Indirect Comparison of the Long-Term Efficacy Maintenance and Adverse Event Rates of Lebrikizumab versus Dupilumab in Moderate-to-Severe Atopic Dermatitis. Dermatol. Ther..

[215] Katoh N., Tsunemi Y., Kamata M., Dossenbach M., Hanada T. An indirect comparison of lebrikizumab, dupilumab, and tralokinumab in Japanese patients with moderate to severe atopic dermatitis by network meta-analysis methodology. Proceedings of the European Academy of Dermatology and Venerology Congress.

[216] Liu W., Zhao Y., He Y., Yan X., Yu J., Song Q., Zhang L., Dong B., Xu G., Wang C. (2024). Stapokibart (CM310) targets IL-4Rα for the treatment of type 2 inflammation. iScience.

[217] Zhao Y., Zhang L., Zhang J. (2024). Efficacy and safety of stapokibart (CM310) in adults with moderate-to-severe atopic dermatitis: A multicenter, randomized, double-blind, placebo-controlled phase 3 trial. J. Am. Acad. Dermatol..

[218] Wang J., White J., Sansone K.J., Spelman L., Sinclair R., Yang X., Pan W., Wei Z. (2023). Rademikibart (CBP-201), a next-generation monoclonal antibody targeting human IL-4Rα: Two phase I randomized trials, in healthy individuals and patients with atopic dermatitis. Clin. Transl. Sci..

[219] Nishi K., Matsumoto H., Sunadome H., Nagasaki T., Oguma T., Tashima N., Hayashi Y., Terada S., Morita K., Yoshimura C. (2024). *IL1RL1*variant may affect the response to type 2 biologics in patients with severe asthma. ERJ Open Res..

[220] Porsbjerg C.M., Townend J., Bergeron C., Christoff G.C., Katsoulotos G.P., Larenas-Linnemann D., Tran T.N., Al-Lehebi R., Bosnic-Anticevich S.Z., Busby J. (2024). Association between pre-biologic T2-biomarker combinations and response to biologics in patients with severe asthma. Front. Immunol..

[221] Thomas V.A., Balthasar J.P. (2019). Understanding Inter-Individual Variability in Monoclonal Antibody Disposition. Antibodies.

[222] Silverberg J.I., Wollenberg A., Legat F.J., Laquer V.T., Armstrong A.W., Herranz P., Naldi L., Ahmad F., Ulianov L., Piketty C. (2024). 667-Maintenance of efficacy and safety with nemolizumab at week 48: Results from two global phase 3 pivotal studies (ARCADIA-1 and ARCADIA-2) in patients with moderate-to-severe atopic dermatitis. Br. J. Dermatol..

[223] Boada-Fernández-Del-Campo C., García-Sánchez-Colomer M., Fernández-Quintana E., Poza-Guedes P., Rolingson-Landaeta J.L., Sánchez-Machín I., González-Pérez R. (2024). Real-World Safety Profile of Biologic Drugs for Severe Uncontrolled Asthma: A Descriptive Analysis from the Spanish Pharmacovigilance Database. J. Clin. Med..

[224] Gargiulo L., Ibba L., Malagoli P., Burroni A.G., Chiricozzi A., Dapavo P., Ferrucci S.M., Gola M., Napolitano M., Ortoncelli M. (2024). Management of Patients Affected by Moderate-to-Severe Atopic Dermatitis with JAK Inhibitors in Real-World Clinical Practice: An Italian Delphi Consensus. Dermatol. Ther..

[225] Padilla-Galo A., García-Ruiz A.J., Abitbol R.C.L., Olveira C., Rivas-Ruiz F., Soler N.G.-A., Morales M.P., Azcona B.V., Tortajada-Goitia B., Moya-Carmona I. (2021). Real-life cost-effectiveness of benralizumab in patients with severe asthma. Respir. Res..

[226] Habash M., Guiang H., Mayers I., Quinton A., Vuong V., Dineen A., Singh S., Gibson D., Turner A.P. (2023). Cost-effectiveness of tezepelumab in Canada for severe asthma. J. Med. Econ..

[227] Tugay D., Top M., Aydin Ö., Bavbek S., Damadoğlu E., Erkekol F.Ö., Kalkan I.K., Kalyoncu A.F., Karakaya G., Oğuzülgen I.K. (2023). Real-world patient-level cost-effectiveness analysis of omalizumab in patients with severe allergic asthma treated in four major medical centers in Turkey. J. Med. Econ..

[228] Anderson W.C., Szefler S.J. (2019). Cost-effectiveness and comparative effectiveness of biologic therapy for asthma: To biologic or not to biologic?. Ann. Allergy Asthma Immunol..

[229] Bousquet J., Shamji M.H., Anto J.M., Schünemann H.J., Canonica G.W., Jutel M., Del Giacco S., Zuberbier T., Pfaar O., Fonseca J.A. (2023). Patient-centered digital biomarkers for allergic respiratory diseases and asthma: The ARIA-EAACI approach—ARIA-EAACI Task Force Report. Allergy.

