# Supplementary material for: Advances in Biologic Therapies for Allergic Diseases: Current Trends, Emerging Agents, and Future Perspectives
Source: J Clin Med. 2025 Feb 8;14(4):1079. doi: 10.3390/jcm14041079 (PMC11856668; doi:10.3390/jcm14041079)
Supplement: Supplementary file 1 [file jcm-14-01079-s001.zip › jcm-3409848-supplementary.docx]

**Supplementary Table S1.** Summary of clinical trials investigating novel biologics in allergic diseases (2019–2024).

| **Drug** | **Molecular target** | **Administration** | **Cohort** | **Study register** | **Study type** | **Phase** | **Main findings/ outcomes** | **Ref.** |
| --- | --- | --- | --- | --- | --- | --- | --- | --- |
| **Asthma** | | | | | | | | |
| Depemokimab | IL-5 | Subcutaneous | Severe asthma with ≥300 blood eosinophils count and  ≥2 asthma exacerbations in the last 12 months. | NCT04719832  NCT04718103 | Randomized, double-blind, placebo-controlled, parallel-group, multicenter study | 3a | Reduction in the AER.  No changes in St. George’s Respiratory Questionnaire score between groups | [43] |
| Lebrikizumab | IL-13 | Subcutaneous | Adults aged 18-75 with uncontrolled asthma with prebronchodilator FEV_1_ 40-80%, and documented bronchodilator reversibility ( ≥12% relative improvement). | NCT02099656 | Randomized, double-blind, placebo-controlled, multicenter study | 2 | No significant change in adjusted mean subepithelial eosinophils/mm^2^ at week 12.  Decrease of subepithelial collagen thickness, and FeNO. | [48] |
| Lebrikizumab | IL-13 | Subcutaneous | Adolescents aged 12-17 with uncontrolled asthma on high-dose ICS for ≥6 months with prebronchodilator FEV_1_ 40-90%. | NCT01875003 | Randomized, double-blind, placebo-controlled, parallel-group, multicenter study | 3 | 37,5 and 125 mg lebrikizumab reduced asthma exacerbation rates by 40 and 51%, respectively, compared to the placebo.  Reduction in exacerbation rate was more prominent in a group with higher compared to lower blood eosinophil count (≥300 vs <300 cells/μl) | [50] |
| Tozorakimab  (MEDI3506) | IL-33 | Subcutaneous | Adults aged between 18-65 with moderate-to-severe uncontrolled, early-onset asthma. | NCT04570657 | Randomized, double-blind, placebo-controlled, parallel-group, multicenter study | 2a | No significant improvement in prebronchodilatator FEV_1_ compared to placebo at week 16.  Tozorakimab improved prebronchodilatator FEV_1_ compared to placebo in patients with ≥2 exacerbations in the previous 12 months. | [54] |
| Astegolimab | ST2/IL-33R | Subcutaneous | Adults aged 18-75 with uncontrolled, severe asthma on high-dose ICS with ≥1 additional controller medication, with FEV_1_ 40-80%. | NCT02918019 | Randomized, double-blind, placebo-controlled, multicenter, multi-arm study | 2b | 490 mg astegolimab resulted in AER reduction of 43% compared to placebo.  490 mg astegolimab resulted in 54% AER reduction in the eosinophil-low subgroup compared to placebo. | [61] |
| Itepekimab | IL-33 | Subcutaneous | Adults aged 18-70 with moderate-to-severe asthma with prebronchodilatator FEV_1_ 50-85%. | NCT03387852 | Randomized, double-blind, placebo-controlled, parallel-group, multicenter study | 2 | 12 weeks of itepekimab was associated with asthma control loss in 22% of patients compared to 27% in combined itepekimab and dupilumab, and 41% in the placebo group.  Itepekimab monotherapy increased prebronchodilatator FEV_1_, asthma control, and QoL compared to placebo.  Combined itepekimab and dupilumab did not improve lung function.  The incidence of adverse events was similar among trial groups with the most mild-to-moderate severity. | [56] |
| Melrilimab (CNTO 7160) | IL33R | Intravenous | Adults with severe asthma, prebronchodilatator FEV_1_ 35-79%, blood eosinophil count ≥300 cells/μl, evidence of allergic fungal airway disease (fungal sensitization to *A.fumigatus* or *P*.*chrysogenum* measured by serum sIgE). | NCT03393806 | Randomized, double-blind, placebo-controlled, parallel-group, multicenter study | 2a | The study was terminated early due to a high rate of screen failure, and low enrollment.  There was no difference in change from baseline in blood eosinophil count at week 12 between melrilimab and placebo.  Melrilimab reduced free sST2 concentration. | [57] |
| Melrilimab (CNTO 7160) | IL33R | Intravenous | Adults with moderate-to-severe asthma using LABA and high-dose ICS for ≥4 months, with ≥1 asthma exacerbation within the last 12 months. | NCT03207243 | Randomized, double-blind, placebo-controlled, parallel-group, multicenter study | 2a | Melrilimab for 16 weeks decreased the rate of asthma control loss compared to placebo (18% reduction).  Treatment-related adverse events (TRAEs) incidence was higher in melrilimab group (10% vs 4%).  The most common TRAEs were cardiac disorders and musculoskeletal/connective tissue disorders. | [59] |
| Risankizumab | IL-23 | Subcutaneous | Adults aged 18-75 with severe asthma, prebronchodilatator FEV_1_ 40-85%, using at least medium dose ICS and at least one other controller medication, with a history of ≥2 exacerbations in the last 12 months. | NCT02443298 | Randomized, double-blind, placebo-controlled, parallel-group, multicenter study | 2a | In the risankizumab-treated group time to first asthma worsening was significantly shorter than in the placebo-treated group (HR 1.46).  The annualized rate of asthma worsening was higher in the risankizumab group.  Risankizumab was well-tolerated. | [65] |
| Ecleralimab (CSJ117) | TSLP | Inhaled | Adults aged 18-60 with stable, mild, atopic asthma requiring no treatment expect SABA < than twice weekly, with FEV_1_ ≥70%. | NCT03138811 | Randomized, double-blind, placebo-controlled, parallel-group, multicenter, bronchoprovocation study | 1 | Ecleralimab at week 12 significantly attenuated late asthmatic response and allergen-induced sputum eosinophil count.  FeNO was significantly reduced from baseline throughout the study, except 24h after the allergen challenge.  Ecleralimab exhibited a good safety profile. | [67] |
| Ecleralimab (CSJ117) | TSLP | Inhaled | Adults aged 18-75 years with severe, uncontrolled asthma, treated with medium or high ICS dose plus LABA with up to additional controllers, with morning prebronchodilatator FEV_1_ 40-85%, | NCT04410523 | Randomized, double-blind, placebo-controlled, parallel-group, multicenter, multi-national study | 2 | Ecleralimab in dose 0.5 mg, but not in doses 1, 2, 4, and 8 mg, did meet the primary endpoint of the study i.e. significant change (12% improvement) from baseline in pre-dose FEV_1_ at week 8 and week 1.  None of the ecleralimab doses resulted in a significant change from baseline in FeNO at week 8 and week 12. | NA; no official publication to date of this manuscript submission |
| REGN1908-1909 (1:1 cocktail of monoclonal antibodies) | Fel d 1 | Subcutaneous | Adults aged 18-55 with positive allergen skin prick test using standardized cat hair extract, positive Fel d 1 sIgE, and a negative galactose‐alpha‐1,3‐galactose sIgE antibody test. | NCT01922661 | Randomized, double-blind, placebo-controlled study | 1 | A single 600 mg dose of REGN1908-1909 maintained mean concentrations in serum above the target level (10 mg/L) for 8-12 weeks.  REGN1908-1909 showed a favorable safety profile and was well-tolerated. | [68] |
| REGN1908-190 (1:1 cocktail of monoclonal antibodies) | Fel d 1 | Subcutaneous | Adults aged 18-65 with mild asthma, history of symptomatic cat-allergen-triggered asthma with rhinitis with or without conjunctivitis, and no cat exposure at home for the past year. | NCT03838731 | Randomized, double-blind, placebo-controlled, parallel-group, single-center study | 2 | A single dose of REGN1908/1909 significantly prevented cat allergen-induced decrease in FEV_1_ from 8 days to 85 days compared with placebo.  The mean FEV_1_ was preserved on days 8, 29, and 57 in the REGN1908-1909-treated group.  A single dose of REGN1909/1909 significantly prevented early asthmatic response to cat allergen, and increased the quantity of cat allergen tolerated relative to the baseline on day 85 compared to placebo (54 vs 12.9 ng). | [69] |
| MTPS9579A | Tryptase | Intravenous | Adults aged 18-75 with uncontrolled asthma treated with ICS and at least one additional controller therapy, with a history of at least 2 asthma exacerbations within the last 12 months. | NCT04092582 | Randomized, double-blind, placebo-controlled, multicenter study | 2a | MTPS9579A did not meet the primary endpoint i.e. time to the first composite exacerbation event- there was no significant difference between MTPS9579A- and placebo-treated groups during the 48-week trial period.  MTPS9579A did not meet any of the secondary endpoints. | [74] |
| **Atopic dermatitis** | | | | | | | | |
| Bermekimab | IL-1α | 1) Subcutaneous  2) Subcutaneous 3) Subcutaneous  4) Intravenous | Participants with moderate-to-severe AD. | NCT03496974  NCT04021862  NCT04791319  NCT04990440 | 1) Open-label, dose-escalation study  2) Double-blind, placebo-controlled, randomized study  3) Double-blind, placebo- and active-comparator-controlled, randomized study  4) Double-blind, placebo-controlled, randomized study | 2 | No efficacy in larger controlled trials. | [99] |
| Lebrikizumab | IL-13 | Subcutaneous | Participants with moderate-to-severe AD. | NCT04146363  NCT04178967 | Randomized, double-blind, placebo-controlled study | 3 | 16-week induction period with lebrikizumab every 2 weeks (Q2W), an IGA score improvement was maintained with lebrikizumab Q2W (71.2%), and Q4W (76.9%), EASI-75 score improvement was maintained in 78.4% in Q2W and 81.7% in Q4W.  63% of patients in the lebrikizumab-treated group report any TEAEs, with most events being mild-to-moderate in severity. | [100] |
| Stapokibart ( CM310) | IL-4 | Subcutaneous | Participants with moderate-to-severe AD, who previously completed parent trials of stapokibart. | NCT04893707 | Multicenter, open-label, nonrandomized study | 2 | High clinical efficacy of stapokibart treatment with acceptable safety profile of the drug. | [84] |
| Itepekimab | IL-33 | 1) Subcutaneous  2) Subcutaneous | Participants with moderate-to-severe AD. | NCT03738423  NCT03736967 | 1) Dose-ranging, randomized, double-blind, placebo-controlled, parallel-group study  2) Proof-of-concept randomized, double-blind, placebo-controlled study | 2 | Trials were prematurely terminated.  No clinical benefits of itepekimab were demonstrated. | [106] |
| Nemolizumab | IL-31Rα | Subcutaneous | Participants with moderate-to-severe AD associated pruritus, and inadequate response to topical steroids. | NCT03985943  NCT03989349 | Replicate, randomized, double-blind, placebo-controlled, multicenter, parallel-group study | 3 | Nemolizumab significantly reduced pruritus scores on the VAS scale and improved EASI scores compared to placebo.  There was no significant difference in the incidence of adverse events compared to placebo. | [110] |
| Cendakimab | IL-13Rα1 and IL-13Rα2 | Subcutaneous | Adult patients with moderate-to-severe AD. | NCT04800315 | Randomized, double-blind, placebo-controlled, parallel-group, dose-ranging study | 2 | Cendakimab was effective, well-tolerated, and generally safe for patients with moderate to severe AD. | [102] |
| Rademikibart (CBP-201) | IL-4Rα | Subcutaneous | Adult patients with moderate-to-severe AD. | NCT04444752  NCT05017480 | Randomized, double-blind, placebo-controlled, multi-centered study | 2 | Studies demonstrated high rates of sustained efficacy, further improvements in treatment outcomes with continued use, and consistent clinically meaningful benefits in pruritus and QoL. | [104,105] |
| Benralizumab | IL-5Rα | Subcutaneous | Adolescents and adults with moderate-to-severe AD who remain symptomatic despite topical therapy, with IGA ≥3, EASI ≥16, BSA ≥10%, PPNRS ≥4. | NCT04605094 | Randomized, double-blind, placebo-controlled, parallel-group, multinational study | 2 | There were no differences between the benralizumab- and placebo-treated groups on the primary endpoint i.e. IGA response (IGA 0/1 and a decrease in IGA score ≥2 points at week 16) compared to baseline.  There was no difference between both groups on any secondary endpoints, including EASI-75, EASI-90, itch improvement, symptom control, and HRQoL. | [114] |
| Eblasakimab | IL-13Rα1 | Subcutaneous | Participants with moderate-to-severe AD. | NCT05158023 | Randomized, double-blind, placebo-controlled study | 2b | Eblasakimab treatment for 8 weeks significantly improved EASI score compared to placebo (-65% vs -27%).  71% of eblaskimab-treated patients (compared to 47% in the placebo group) experienced TEAEs with most of them being mild-to-moderate severity. | [103] |
| PF-06817924 | IL-33 | Intravenous | 1) Healthy participants.  2) Patients with chronic sinusitis with nasal polyps.  3) Patients with severe AD | NCT02743871 | Randomized, placebo-controlled study | 1 | The drug was well tolerated in all patient groups.  The study showed the linear pharmacokinetics of the drug.  Participants with AD had the lowest percentage of anti-drug antibody production compared to other study groups. | [107] |
| Ligelizumab | IgE | Subcutaneous | Participants with moderate-to-severe AD. | EudraCT Number 2011-002112-84 | Randomized, double-blind, placebo-controlled, parallel-group, proof-of-concept study | 2 | The efficacy of ligelizumab was not significantly better compared to placebo.  Patients with high baseline IgE levels responded better to treatment than patients with low baseline IgE. | [115] |
| Amlitelimab | OX40 | Intravenous | Participants with moderate-to-severe atopic dermatitis. | NCT03754309 | Multi-center, randomized double-blind, placebo-controlled, parallel-group study | 2a | The drug was well tolerated, with no hypersensitivity events demonstrated.  Amlitelimab treatment resulted in a decrease in EASI score with the greatest intensity at weeks 2-16. | [117] |
| Rocatinlimab | OX40 | Subcutaneous | Participants with moderate-to-severe AD | NCT03703102 | Multicenter, randomized, placebo-controlled, double-blind, parallel-group study | 2b | Rocatinlimab treatment led to progressive improvements in AD, with most patients maintaining these benefits even after discontinuation.  The treatment was well tolerated. | [118] |
| Spesolimab | IL-36R | Intravenous | Participants with moderate-to-severe AD | NCT03822832 | Multicenter, randomized, double-blind, placebo-controlled | 2a | The study showed that the drug was well tolerated, and at week 16 the EASI score decreased | [120] |
| Astegolimab | IL-33/ST2 | Subcutaneous | Participants with chronic AD. | NCT03747575 | Randomized, double-blind, placebo-controlled multicenter | 2 | Despite the good tolerability of the drug, astegolimab did not show clinical efficacy in patients with AD. | [108] |
| Melrilimab (CNTO 7160) | IL-33R | Intravenous | Part 1) Healthy participants  Part 2) Subjects with mild asthma or mild atopic dermatitis. | NCT02345928. | Randomized, double-blind, placebo-controlled study | 1 | The results of the study indicate good tolerability of the drug, with a single situation of a severe adverse event.  Although target engagement was confirmed, no clear clinical activity of CNTO 7160 was observed in patients with asthma or AD. | [58] |
| YH35324 | IgE |  | 1) Healthy participants  2) Patients with mild allergic rhinitis, atopic dermatitis, food allergy, or urticaria, and a serum total IgE level of 30-700 IU/mL (Part A)  3) Patients with mild allergic rhinitis, atopic dermatitis, food allergy, or urticaria, and a serum total IgE level of > 700 IU/mL (Part B). | NCT05061524 | Randomized, double-blind, placebo/active-controlled, single ascending dose study | 1 | The study showed a good safety profile for the drug.  YH35324 treatment was associated with a reduction of serum-free IgE levels in subjects with atopic conditions including AD. | [121] |
| Tezepelumab | TSLP | Subcutaneous | Adults with moderate-to-severe AD, affecting ≥10% of BSA, EASI score ≥12, IGA ≥3, treated with topical corticosteroids (TCS) | NCT02525094 | Randomized, double-blind, placebo-control, multicenter study | 2a | Tezepelumab plus TCS was associated with a numerically greater percentage of EASI50 achievement at week 12 compared to placebo (64.7% vs 48.2%) with no statistical significance.  TEAE incidence was similar between treatment groups. | [123] |
| Tezepelumab | TSLP | Subcutaneous | Adults with moderate-to-severe AD, affecting ≥10% of BSA, EASI score ≥16, IGA ≥3, with a history of inadequate response to treatment with TCS. | NCT03809663 | Randomized, double-blind, placebo-controlled, dose-ranging study | 2b | The study did not meet his primary endpoints i.e. IGA score of 0 or 1 at week 16 and a 75% reduction from baseline in EASI75 score at week 16. | NA; no official publication to date of manuscript submission |
| Solrikitug (MK-8226) | TSLP | Intravenous | Adults with moderate-to-severe AD (affecting ≥10% of BSA, EASI score ≥16, IGA ≥3), with a history of inadequate response to a stable regimen of TCS or calcineurin inhibitors. | [NCT01732510](https://clinicaltrials.gov/study/NCT01732510) | Randomized, double-blind, placebo-controlled, multiple rising dose study | 1b | Solrikitug at a dose of 3 mg/kg significantly reduced the EASI score compared to placebo after 12 weeks of treatment.  10 out of 13 patients in the 3 mg/kg subgroup experienced at least one adverse event compared to 7 out of 8 in the placebo group. No one in solrikitug-treated discontinued the study drug due to an adverse event. | NA; no official publication to date of manuscript submission |
| **Chronic spontaneous urticaria** | | | | | | | | |
| Dupilumab | IL-4Rα | Subcutaneous | Study A: omalizumab naive  Study B: omalizumab-intolerant or incomplete responders.  All patients were symptomatic despite H1-AH treatment. | NCT04180488 | Randomized, double-blind, placebo-controlled, parallel-group, multicenter study | 3 | In study A: UAS7 and ISS7 improved significantly compared to the placebo  In study B: small improvement in UAS7 and ISS7 without statistical significance. | [139] |
| YH35324 | IgE | Subcutaneous | Healthy subjects with atopy or subjects with mild allergic diseases including CSU.  Serum total IgE level of 30 to 700 IU/mL or > 700 IU/mL. | NCT05061524 | Randomized, double-blind, placebo/active-controlled, single ascending dose study | 1 | No serious adverse events (AE), AE-related treatment discontinuation, or anaphylaxis were reported.  YH35324 significantly suppressed serum-free IgE levels with longer duration than standard treatment omalizumab. | [121] |
| Benralizumab | IL-5Rα | Subcutaneous | Adult, symptomatic patients despite receiving standard of care including second-generation H1 antihistamine treatment as monotherapy or combined with LTRAs and/or H2 blockers. | NCT04612725 | Randomized, double-blind, placebo-controlled, parallel-group, multicenter study | 2b | No changes from baseline ISS7 and UAS7 scores at week 12 compared to the placebo.  Benralizumab resulted in significant blood eosinophil level depletion at week 24. | [142] |
| Ligelizumab | IgE | Subcutaneous | Subjects ≥ 12 years of age with  CSU refractory to non-sedating H1-AH. | NCT03580369 (PEARL-1), NCT03580356 (PEARL-2) | Randomized, double-blind, placebo-controlled, parallel-group, multicenter study | 3 | Both studies demonstrated significant improvement in UAS7 score compared to placebo but not to omalizumab. | [146] |
| Ligelizumab | IgE | Subcutaneous | Subjects 17 to 75 years with CSU for at least 6 months remaining refractory to standard care. | NCT02477332 | Randomized, double-blind, placebo, and active-controlled multicenter study | 2b | Ligelizumab in 72 mg or 240 mg was associated with a higher percentage of patients with complete control of symptoms compared to omalizumab at 300 mg or placebo. | [145] |
| Lirentelimab | Siglec-8 | Intravenous | Adults with CSU refractory to antihistamine treatment in single or 4-fold dosage. | NCT03436797 | Open-label study | 2a | In omalizumab-naive and omalizumab-refractory cohorts, lirentelimab resulted in disease activity decrease (at 22 weak) of 73% and 47% respectively with UAS7 response rates of 77% and 45% respectively.  No treatment-related serious AEs were reported. | [151] |
| Lirentelimab | Siglec-8 | Subcutaneous | Adults with moderate-to-severe CSU refractory to H1-antihistamine at a minimum of the licensed dose. | [NCT05528861](https://clinicaltrials.gov/study/NCT05528861) | Randomized, double-blind, placebo-controlled, multicenter study | 2 | Lirentelimab did not meet his primary endpoint i.e. absolute change in weekly UAS7 score from baseline at week 12.  Lirentelimab did not meet any secondary endpoints including absolute change in HSS7 from baseline at week 12, and absolute change in ISS7 from baseline at week 12. | NA; no official publication to date of manuscript submission |
| Canakinumab | IL-1ꞵ | Subcutaneous | Adults with moderate to severe CSU remaining symptomatic despite the use of non-sedating antihistamines with or without LTRAs/corticosteroids. | NCT01635127 | Randomized, double-blind, placebo-controlled, single-center study | 2 | No significant changes in the UAS7 score compared to the placebo at week 4.  No significant difference between canakinumab and placebo in clinical improvement at week 8.  Canakinumab was well tolerated with mild AEs reported. | [153] |
| **Non-esophageal eosinophilic gastrointestinal disorders** | | | | | | | | |
| Lirentelimab (AK002) | Siglec-8 | Subcutaneous | -Aged 18-80  -Biopsy ≥30 eosinophils/hpf in 5 hpf in the stomach and/or ≥30 eosinophils/hpf in 3 hpf in the duodenum  -Inadequate/loss of response to standard therapies. | [NCT04322604](https://www.clinicaltrials.gov/study/NCT04322604?cond=NCT04322604&rank=1) | Multi-center, randomized, double-blind, placebo-controlled | 3 | Lirentelimab met his primary endpoint i.e. proportion with gastric eosinophils ≤4 eos/hpf and mean absolute change in total symptom score.  Lirentelimab did not alleviate symptoms reported by patients. | NA; no official publication to date of manuscript submission |
| Lirentelimab (AK002) | Siglec-8 | Subcutaneous | -Age 18-80  -Biopsy ≥30 eosinophils/hpf in 3 hpf in the duodenum  -Symptomatic (abdominal pain, nausea, or diarrhea)  -Intolerant to or failed standard treatments. | [NCT04856891](https://clinicaltrials.gov/study/NCT04856891?cond=NCT04856891&rank=1) | Multi-center, randomized, double-blind, placebo-controlled | 3 | The trial met its histologic co-primary endpoint i.e. proportion with duodenal eosinophils ≤15 eos/hpf.  Lirentelimab did not achieve statistical significance on the patient-reported symptomatic co-primary endpoint. | NA; no official publication to date of manuscript submission |
| Lirentelimab (AK002) | Siglec-8 | Subcutaneous | -Prior completion of NCT04322604 trial  -Aged 18-80  -Biopsy ≥30 eosinophils/hpf in 5 hpf in the stomach and/or ≥30 eosinophils/hpf in 3 hpf in the duodenum  -Inadequate/loss of response to standard therapies. | [NCT04620811](https://clinicaltrials.gov/study/NCT04620811?cond=NCT04620811&rank=1) | Multi-center, open-label, extension study | 3 | The incidence of TEAE during the extended study period was higher in the group treated initially with a placebo (in the main study) compared to the group treated only with lirentelimab (during the main and extended study) (44% vs 34.5%).  Extended treatment with lirentelimab is associated with a higher change in patient-reported Total Symptom Score (TSS) compared to the group initially treated with a placebo (in the main study).  Tissue eosinophil response was more frequent in the placebo+lirentelimab group (96.3% vs 92.3%) compared to only the lirentelimab group. | NA; no official publication to date of manuscript submission |
| Lirentelimab (AK002) | Siglec-8 | Subcutaneous | -Aged 18-80  -Biopsy ≥30 eosinophils/hpf in 5 hpf in the stomach and/or ≥30 eosinophils/hpf in 3 hpf in the duodenum without any other cause for the gastric eosinophilia  -Inadequate/failure of response to standard therapies  -Moderate-to-severe symptoms. | NCT03496571 | Multicenter, randomized, double-blind, placebo-controlled | 2 | Lirentelimab treatment was associated with a significant reduction in gastrointestinal eosinophilia (95% compared to 10% in the placebo group).  63% of patients responded to lirentelimab treatment (defined as >30% reduction in total symptom score and >75% reduction in gastrointestinal eosinophil count) compared to 5% in the placebo group.  Lirentelimab was associated with significant improvement in TSS compared to placebo (48% vs 22%).  Lirenteliamb was well tolerated with an increased prevalence of mild-to-moderate infusion-related reactions compared to placebo (60% vs 23%). | [167] |
| Lirentelimab (AK002) | Siglec-8 | Subcutaneous | -Prior completion of NCT03496571 trial  -Aged 18-80  -Biopsy ≥30 eosinophils/hpf in 5 hpf in the stomach and/or ≥30 eosinophils/hpf in 3 hpf in the duodenum without any other cause for the gastric eosinophilia  -Inadequate/failure of response to standard therapies  -Moderate-to-severe symptoms. | [NCT03664960](https://clinicaltrials.gov/study/NCT03664960) | Multicenter, open-label, extension Study | 2 | Extended treatment with lirentelimab is associated with a lower change in patient-reported Total Symptom Score (TSS) compared to the group initially treated with a placebo (in the main study).  The percentage of tissue eosinophil reduction was higher in the group treated with extender lirentelimab compared to the group initially treated with a placebo (99.7% vs 93.5%). | NA; no official publication to date of manuscript submission |
| Benralizumab | IL-5Rα | Subcutaneous | Age 12-60 with symptomatic and histologically active EoG (eosinophil count ≥ 30 per hpf in ≥5 hpfs) with blood eosinophilia count >500 cells/µL at least once in the 6 months prior to enrollment. | NCT03473977 | Randomized, double-blind, placebo-controlled | 2 | Benralizuamb treatment for 12 weeks was associated with significantly higher remission rates compared to placebo (77% vs 8%).  Benralizuamb resulted in improvement in EoG histology total score, histology inflammatory score, and blood eosinophil levels.  There were no statistically significant changes in the SODA pain intensity score, SODA non-pain symptom score, SODA satisfaction score, or PROMIS pain intensity between both groups. | [170] |
| **Chronic rhinosinusitis with nasal polyps** | | | | | | | | |
| Dupilumab | IL-4Rα | Subcutaneous | Adult patients with severe, diffuse, uncontrolled CRSwNP with a history of at least two cycles of SCS over the last year and/or of previous ESS. | NCT04181190 | Real-life, observational, multicenter study | 4 | Dupilumab reduced polyp size and improved QoL, severity of symptoms, nasal congestion, and smell. | [186] |
| Dupilumab | IL-4Rα | Subcutaneous | Severe, bilateral CRSwNP despite INCS treatment with previous systemic corticosteroids (in the last 2 years) or previous sinonasal surgery. | NCT02898454 | Randomized, multicenter, double-blind, placebo-controlled, parallel-group study | 3 | Dupilumab treatment was associated with a significantly lower incidence of upper and lower respiratory tract infections in patients with CRSwNP (38% lower).  Dupilumab-treated patients had significantly less systemic anti-infective medication used (49% lower compared to placebo). | [187] |
| Dupilumab | IL-4Rα | Subcutaneous | Adults with severe, bilateral CRSwNP with prior SCS treatment or contraindication/intolerance to SCS or prior sinonasal surgery. | NCT02912468  NCT02898454 | Randomized, multicenter, double-blind, placebo-controlled, parallel-group studies | 3 | Dupilumab significantly improved nasal congestion (NC) score in patients with ≥3 prior sinus surgeries than in patients with no prior surgery.  Dupilumab significantly improved nasal polyp score (NPS) and Lund-Mackay (LMK) score in patients <3 years after sinonasal surgery compared with the ≥5 to <10 and ≥10 years subgroups.  Dupilumab significantly reduced SCS use and/or rate of sinonasal surgery compared to placebo during the trial period. | [188] |
| Mepolizumab | IL-5 | Subcutaneous | Blood eosinophil >2%, endoscopic bilateral NP score ≥5, nasal obstruction VAS score >5, ≥2 sinonasal symptoms, and either previous sinus surgery or SC use/intolerance. | NCT04607005 | Randomized, double-blind, placebo-controlled, parallel-group | 3 | Mepolizumab is effective (significantly improved nasal obstruction and ENPS score) and well-tolerated (no serious adverse events) in patients with CRSwNP/ECRS. | [194] |
| Mepolizumab | IL-5 | Subcutaneous | Adults with severe, bilateral CRSwNP eligible for repeat surgery despite standard-of-care treatment using stable maintenance therapy with mometasone furoate for ≥8 weeks before screening | NCT03085797 | Randomized, double-blind, placebo-controlled, parallel-group, multi-center trial | 3 | Mepolizumab reduced the risk of further sinus surgery irrespective of the time since the last sinus surgery and blood eosinophil count. | [196] |
| Mepolizumab | IL-5 | Subcutaneous | Adults with severe bilateral CRSwNP with a history of at least one previous surgery in the previous 10 years for the removal of nasal polyps | NCT03085797 | Randomized, double-blind, placebo-controlled, parallel-group, multi-center trial | 3 | 52 weeks of mepolizumab treatment resulted in significant improvement of nasal polyp size and nasal obstruction with no new safety indications. In addition, mepolizumab treatment improved CRSwNP-associated loss of smell and taste.  52 weeks of mepolizumab significantly decreased the risk of SCS use compared to placebo (37.5% vs 25.4%)- SCS-sparing effect.  In patients requiring at least 1 dose of SCS, treatment with mepolizumab was associated with a decrease in total prednisolone-equivalent oral CS dose (irrespective of prior sinus surgeries and blood eosinophilia). | [193,195] |
| Mepolizumab | IL-5 | Subcutaneous | Adults with severe bilateral CRSwNP with a history of at least one previous surgery in the previous 10 years for the removal of nasal polyps and ≥8 weeks of stable mometasone furoate maintenance treatment before screening. | NCT03085797 | Randomized, double-blind, placebo-controlled study | 3 | Fifty-two weeks of mepolizumab treatment is associated with partially sustained clinical benefits (including overall symptoms, QoL, corticosteroid use) up to 24 weeks follow-up after biologics discontinuation vs placebo. | [197] |
| Benralizumab | IL-5Rα | Subcutaneous | Patients aged 18-75 years with a diagnosis of severe, eosinophilic asthma with at least 2 prior exacerbations in the last year despite treatment with medium to high-dosage of ICS plus additional controllers with physician-diagnosed nasal polyps of any severity | NCT03170271 | Randomized, double-blind, placebo-controlled, parallel-group, multicenter study | 3b | Among patients with high baseline SNOT-22 scores, benralizumab was associated with significantly improved nasal symptoms compared to placebo.  Benralizumab was associated with a 69% reduction in annualized asthma exacerbation rate (AER), improvement in SGRQ total score, FEV_1_, and ACQ-6 scores compared to placebo.  The incidence of adverse events was similar between the benralizuamb and placebo groups. | [201] |
| Benralizumab | IL-5Rα | Subcutaneous | Aged 18-75 years with severe, bilateral CRSwNP despite maintenance treatment with INCS for at least 4 weeks and a history of SCS use and/or sinus surgery. | NCT03401229 | Randomized, double-blind, placebo-controlled, parallel-group, international, multicenter study | 3 | Blood eosinophils were almost fully depleted between weeks 16 and 56 of the study.  Nasal polyp tissue eosinophils were significantly reduced in the benralizumab group (from 57.6 cells mm−2 at baseline to 0 cells mm−2 at week 56) compared to the placebo. | [198] |
| Benralizumab | IL-5Rα | Subcutaneous | Aged 18-75 years with severe, bilateral CRSwNP despite maintenance treatment with INCS for at least 4 weeks and a history of SCS use and/or sinus surgery. | NCT03401229 | Randomized, double-blind, placebo-controlled, parallel-group, multi-center trial | 3 | 40 weeks of benralizumab significantly improved NPS, nasal blockage score, and sense of smell score compared to placebo.  Benralizumab was well tolerated. During the period of study, 77.3% and 78.8% of patients in the benralizumab and placebo groups, respectively, experienced at least 1 adverse event. Most of them were mild-to-moderate in severity. | [199] |
| Benralizumab | IL-5Rα | Subcutaneous | Bilateral CRSwNP patients aged between 20-75 years with weight **≥** 40 kg and a total ECRS score of **≥**11. | NCT02772419 | Multicenter, randomized, double-blind, placebo-controlled trial | 2 | Benralizumab did not meet the primary efficacy endpoint (change in nasal polyp score from baseline at week 12 between benralizumab and placebo group).  Benralizuamb treatment was associated with a decrease of NPS of at least 2 points in 42.2% of patients.  Greater blood eosinophil count was associated with greater tendency to benralizuamb response. | [200] |
| PF-06817024 | IL-33 | Subcutaneous, intravenous | Females of non-childbearing potential and males aged 18-64 years with symptomatic (at least 2 symptoms listed in study protocol), bilateral CRSwNP with minimum NPS 5/8 score | NCT02743871 | Randomized, double-blind, third-party open, placebo-controlled, dose-escalating study | 1 | 20 patients with CRSwNP were randomized with 16 completing treatment.  45.5% of participants treated with PF-06817024 experienced TEAEs with the most common reported being general disorders and administration site conditions.  Most treatment-related TEAEs were mild-to-moderate in severity. | [107] |
| **Allergic rhinitis** | | | | | | | | |
| Dupilumab | IL-4Rα | Subcutaneous | Participants with CRSwNP with coexisting AR and patients with CRSwNP without AR. | NCT02912468  NCT02898454 | Randomized, double-blind, placebo-controlled, parallel assignment study | 3 | Duplimumab improved measures of CRSwNP and reduced the use of steroids or surgical treatment compared to placebo. | [209] |
| Tezepelumab | TSLP | Subcutaneous | Participants with cat-allergen-induced AR during subcutaneous allergen immunotherapy (SCIT). | NCT02237196 | Randomized, triple-masking, placebo-controlled, parallel assignment study | 2 | Tezepelumab strengthened the effect of SCIT during its duration and also reduced the clinical response one year after the end of therapy in patients with AR. | [210] |
| REGN1908-1909 | Fel d 1 | Subcutaneous | Participants with cat allergen-induced AR. | NCT02127801 | Randomized, double-blind, placebo-controlled study | 1b | A single dose of REGN1908-1909 significantly reduced nasal symptoms and inhibited the allergic response mediated by FcεRI- and FcεRII (CD23) and T-cell activation. | [211] |
| Stapokibart (CM310) | IL-4Rα | Subcutaneous | Participants with uncontrolled seasonal allergic rhinitis (SAR). | NCT05470647 | Randomized, double-blind, placebo-controlled, parallel assignment study | 2 | Administration of stapokibart did not significantly improve the clinical status of patients with uncontrolled seasonal AR. | [212] |
| REGN5713-5714-5715 | Bet v 1 | Subcutaneous | Adult patients with SAR. | [NCT04709575](https://clinicaltrials.gov/study/NCT04709575?cond=NCT04709575&rank=1) | Randomized, double-blind, placebo-controlled, parallel-group study | 3 | A single dose of REGN5713-5714-5715 improved symptom control compared to the placebo group. | NA; no official publication to date of manuscript submission |

**Supplementary Table S2.** Summary of ongoing clinical trials investigating novel biologics in allergic diseases.

| **Drug** | **Molecular target** | **Administration** | **Cohort** | **Study register** | **Study type** | **Phase** | **Status** |  |  |  |  |
| --- | --- | --- | --- | --- | --- | --- | --- | --- | --- | --- | --- |
| **Asthma** | | | | | | | |  |  |  |  |
| MG-K10 | IL-4Rα | Subcutaneous | Adults with moderate-to-severe asthma with prebronchodilatator FEV_1_ ≤80%, at least one severe acute asthma attack within 12 months. | [NCT05382910](https://www.clinicaltrials.gov/study/NCT05382910?cond=NCT05382910%20&rank=1) | Randomized, placebo-controlled study | 1b/2 | Recruiting |  |  |  |  |
| Telikibart (GR1802) | IL-4Rα | Subcutaneous | Adults aged 18-70 with uncontrolled, moderate-to-severe asthma, with prebronchodilatator FEV_1_ 35-80%, with a history of at least one severe asthma exacerbation in the last 12 months. | [NCT06642961](https://clinicaltrials.gov/study/NCT06642961?cond=NCT06642961&rank=1) | Randomized, double-blind, placebo-controlled, multicenter study | 2 | Recruiting |  |  |  |  |
| FB825 | CεmX domain of membrane IgE (mIgE) | Intravenous | Adults aged 18-75 with moderate-to-severe asthma, with prebronchodilatator FEV_1_ 40-80%, using medium or high-dose ICS plus LABA, with total serum IgE ≥360 IU/mL. | [NCT05008965](https://www.clinicaltrials.gov/study/NCT05008965?cond=NCT05008965&rank=1) | Randomized, double-blind, placebo-controlled study | 2 | Recruiting |  |  |  |  |
| Stapokibart (CM310) | IL-4Rα | Intravenous | Individuals with 12-75 years old with moderate-to-severe asthma, prebronchodilatator FEV_1_ ≤80%, using medium or high-dose ICS plus at least one controller drug, experienced at least one severe asthma exacerbation within 12 months. | [NCT05761028](https://www.clinicaltrials.gov/study/NCT05761028?cond=NCT05761028&rank=1) | Randomized, double-blind, placebo-controlled, multicenter study | 2/3 | Recruiting |  |  |  |  |
| Solrikitug (MK-8226) | IlL-4Rα | Subcutaneous | Adults aged 18-75 with uncontrolled asthma, using ICS plus LABA for at least 3 months, and FEV1 of ≥50% to 90%. | [NCT06496607](https://www.clinicaltrials.gov/study/NCT06496607?cond=NCT06496607&rank=1) | Randomized, double-blind, placebo-controlled, multiple dose-ranging study | 2a | Recruiting |  |  |  |  |
| Depemokimab vs mepolizumab or benralizumab | IL-5 | Subcutaneous | Individuals aged ≥12 years receiving either mepolizumab 100 mg or benralizuamb 30 mg for ≥12 months prior to screening with documented benefit from anti-IL-5/IL-5R therapy, requiring regular medium-to-high dose of ICS with or without maintenance OCS, using at least one additional controller medication. | [NCT04718389](https://www.clinicaltrials.gov/study/NCT04718389?cond=NCT04718389&rank=1) | Randomized, double-blind, double-dummy, parallel-group, multicenter, non-inferiority study | 3 | Active, not recruiting |  |  |  |  |
| Depemokimab | IL-5 | Subcutaneous | Participants who completed either SWIFT-1 (NCT04719832) or SWIFT-2 (NCT04718103) study. | [NCT05243680](https://www.clinicaltrials.gov/study/NCT05243680?cond=NCT05243680&rank=1) | Open-label, single-arm, multicenter, extension study | 3 | Active, not recruiting |  |  |  |  |
| IBI3002 | IL-4Rα, TSLP | Subcutaneous | Adults aged 18-55 with documented diagnosis of asthma for at least 12 months, elevated FeNO, stable step 1-3 controller treatment (according to GINA 2023 guidelines), with prebronchodilatator FEV_1_ ≥60%. | [NCT06213844](https://www.clinicaltrials.gov/study/NCT06213844?cond=NCT06213844&rank=1) | Randomized, double-blind, placebo-controlled, single-center, single-ascending dose study | 1 | Recruiting |  |  |  |  |
| Lunsekimig (SAR443765) | IL-13/TSLP | Subcutaneous | Adults aged 18-80 with mild-to-moderate asthma for more than 12 months, at least 1 asthma exacerbation in the last 12 months, prebronchodilatator FEV_1_ ≥40%. | [NCT06676319](https://www.clinicaltrials.gov/study/NCT06676319) | Randomized, double-blind, placebo-controlled, parallel-group, two-arm study | 2 | Recruiting |  |  |  |  |
| Lunsekimig (SAR443765) | IL-13/TSLP | Subcutaneous | Adults aged 18-80 with uncontrolled, moderate-to-severe asthma for ≥12 months, using moderate-to-high doses of ICS in combination with 1-2 additional controller medications, with at least 1 asthma exacerbation in the past 12 months. | [NCT06102005](https://www.clinicaltrials.gov/study/NCT06102005?cond=NCT06102005&rank=1) | Randomized, double-blind, placebo-controlled, parallel-group, dose-ranging, multicenter study | 2 | Recruiting |  |  |  |  |
| Lunsekimig (SAR443765) | IL-13/TSLP | Subcutaneous | Participants who completed the 48-week trial (NCT06102005), with stable background therapy of moderate-to-high ICS in combination with 1-2 additional controller medications with or without OCS. | [NCT06609239](https://www.clinicaltrials.gov/study/NCT06609239?cond=NCT06609239&rank=1) | Open-label, extension study | 2 | Recruiting |  |  |  |  |
| **Atopic dermatitis** | | | | | | | |  |  |  |  |
| Lebrikizumab | IL-13 | Subcutaneous | Adult and adolescent patients with moderate-to-severe AD. | [NCT05916365](https://www.clinicaltrials.gov/study/NCT05916365?cond=NCT05916365%20&rank=1) | Interventional, single-group assignment, open-label study | 3 | Active, not recruiting |  |  |  |  |
| Lebrikizumab | IL-13 | Subcutaneous | Adult and adolescent patients with moderate-to-severe AD. | [NCT04392154](https://www.clinicaltrials.gov/study/NCT04392154?cond=NCT04392154&rank=1) | Interventional, non-randomized study | 3 | Active, not recruiting |  |  |  |  |
| Lebrikizumab | IL-13 | Subcutaneous | Adult and adolescent participants with moderate-to-severe AD who were previously treated with dupilumab. | [NCT05369403](https://www.clinicaltrials.gov/study/NCT05369403?cond=NCT05369403&rank=1) | Interventional, single-group assignment, open-label study | 3 | Active, not recruiting |  |  |  |  |
| Lebrikizumab | IL-13 | Subcutaneous | Adult and adolescent participants with moderate-to-severe AD. | [NCT06526182](https://www.clinicaltrials.gov/study/NCT06526182?cond=NCT06526182&rank=1) | Interventional, single-group assignment, open-label study | 3 | Recruiting |  |  |  |  |
| Lebrikizumab | IL-13 | Subcutaneous | Adult and adolescent participants with moderate-to-severe AD. | [NCT05990725](https://www.clinicaltrials.gov/study/NCT05990725?cond=NCT05990725&rank=1) | Interventional, single-group assignment, open-label study | 3 | Recruiting |  |  |  |  |
| Stapokibart ( CM310) | IL-4Rα | Injection, not specified | Adolescent patients with moderate-to-severe AD. | [NCT06277765](https://www.clinicaltrials.gov/study/NCT06277765?cond=NCT06277765&rank=1) | Multi-center, randomized, double-blind, placebo-controlled study | 3 | Not yet recruiting |  |  |  |  |
| Stapokibart ( CM310) | IL-4Rα | Injection, not specified | Adolescent patients with moderate-to-severe AD. | [NCT06495229](https://www.clinicaltrials.gov/study/NCT06495229?cond=NCT06495229&rank=1) | Interventional, multi-center, single-arm, open-label study | 3 | Not yet recruiting |  |  |  |  |
| Stapokibart ( CM310) | IL-4Rα | Injection, not specified | Adult patients with moderate-to-severe AD. | [NCT06116565](https://www.clinicaltrials.gov/study/NCT06116565?cond=NCT06116565&rank=1) | Open-label, single-arm, multicenter study | 2 | Not yet recruiting |  |  |  |  |
| Telikibart (GR1802) | IL-4Rα | Subcutaneous | Adult patients with moderate-to-severe AD with inadequate response or intolerance to topical GC. | [NCT06216392](https://clinicaltrials.gov/study/NCT06216392?cond=GR-1802&rank=1) | Randomized, double-blind, placebo-controlled, multicenter study | 3 | Not yet recruiting |  |  |  |  |
| Nemolizumab | IL-31Rα | Subcutaneous | Adult and adolescent participants with moderate-to-severe AD. | [NCT03989206](https://www.clinicaltrials.gov/study/NCT03989206?cond=NCT03989206&rank=1) | Interventional, prospective, multicenter, long-term study | 3 | Active, not recruiting |  |  |  |  |
| Nemolizumab | IL-31Rα. | Subcutaneous | Children patients with moderate-to-severe AD | [NCT04921345](https://www.clinicaltrials.gov/study/NCT04921345?cond=NCT04921345&rank=1) | Interventional, multicenter, open-label, single-group study | 2 | Recruiting |  |  |  |  |
| Rademikibart (CBP-201) | IL-4Rα | Subcutaneous | Adult patients with moderate-to-severe AD. | [NCT05905133](https://www.clinicaltrials.gov/study/NCT05905133?cond=NCT05905133&rank=1) | Interventional, single-arm, open-label, multicenter study | 2 | Active, not recruiting |  |  |  |  |
| Eblasakimab (ASLAN004) | IL-13Rα1 | Subcutaneous | Participants with moderate-to-severe AD were previously treated with dupilumab. | [NCT05694884](https://www.clinicaltrials.gov/study/NCT05694884?cond=NCT05694884&rank=1) | Multicenter, randomized, double-blind, placebo-controlled, parallel-arm study | 2 | Recruiting |  |  |  |  |
| Amlitelimab | OX40 | Subcutaneous | Adult and adolescent participants with moderate-to-severe AD who have had an inadequate response to prior biologic or oral JAKi therapy. | [NCT06241118](https://www.clinicaltrials.gov/study/NCT06241118?cond=NCT06241118&rank=1) | Parallel-group, multinational, multicenter, randomized, double-blind, placebo-controlled study | 3 | Recruiting |  |  |  |  |
| Amlitelimab | OX40 | Subcutaneous | Adult and adolescent participants with moderate-to-severe AD. | [NCT06224348](https://www.clinicaltrials.gov/study/NCT06224348?cond=NCT06224348&rank=1) | Parallel group, multinational, multicenter, randomized, double-blind, placebo-controlled, three-arm study | 3 | Recruiting |  |  |  |  |
| Amlitelimab | OX40 | Subcutaneous | Adult and adolescent participants with moderate-to-severe AD. | [NCT06407934](https://www.clinicaltrials.gov/study/NCT06407934?cond=NCT06407934&rank=1) | Multinational, multicenter, randomized, double-blind, placebo-controlled, parallel-group study | 3 | Recruiting |  |  |  |  |
| Amlitelimab | OX40 | Subcutaneous | Adult and adolescent participants with moderate-to-severe AD. | [NCT06181435](https://www.clinicaltrials.gov/study/NCT06181435?cond=NCT06181435&rank=1) | Parallel-group, multinational, multicenter, randomized, double-blind, placebo-controlled, three-arm study | 3 | Recruiting |  |  |  |  |
| Amlitelimab | OX40 | Subcutaneous | Adult and adolescent participants with moderate-to-severe AD. | [NCT06130566](https://www.clinicaltrials.gov/study/NCT06130566?cond=NCT06130566&rank=1) | Parallel group, multinational, multicenter, randomized, double-blind, placebo-controlled, three-arm monotherapy study | 3 | Recruiting |  |  |  |  |
| Amlitelimab | OX40 | Subcutaneous | Participants with moderate to severe atopic dermatitis (AD) who have previously been enrolled in an amlitelimab clinical trial. | [NCT05492578](http://clinicaltrials.gov/study/NCT05492578?cond=NCT05492578&rank=1) | Interventional, single-group assignment, open-label study | 2/3 | Recruiting |  |  |  |  |
| Amlitelimab | OX40 | Subcutaneous | Adult and adolescent participants with moderate-to-severe AD. | [NCT05769777](https://www.clinicaltrials.gov/study/NCT05769777?cond=NCT05769777&rank=1) | Open-label, multinational, multicenter, single-arm study | 2 | Recruiting |  |  |  |  |
| Rocatinlimab | OX40 | Subcutaneous | Adolescent participants with moderate-to-severe AD. | [NCT05633355](https://www.clinicaltrials.gov/study/NCT05633355) | Interventional, single-group assignment, open-label study | 3 | Active, not recruiting |  |  |  |  |
| Rocatinlimab | OX40 | Subcutaneous | Adult and adolescent participants with moderate-to-severe AD. | [NCT06224192](https://www.clinicaltrials.gov/study/NCT06224192) | Multicenter, randomized, open-label study | 3 | Recruiting |  |  |  |  |
| Rocatinlimab | OX40 | Subcutaneous | Adult patients with moderate-to-severe AD. | [NCT05398445](https://www.clinicaltrials.gov/study/NCT05398445) | Interventional, randomized, placebo-controlled, double-blind study | 3 | Active, not recruiting |  |  |  |  |
| Rocatinlimab | OX40 | Subcutaneous | Adult patients with moderate-to-severe AD. | [NCT05724199](https://www.clinicaltrials.gov/study/NCT05724199) | Interventional, randomized, placebo-controlled, double-blind study | 3 | Active, not recruiting |  |  |  |  |
| Rocatinlimab | OX40 | Subcutaneous | Adult and adolescent with moderate-to-severe AD. | [NCT05882877](https://www.clinicaltrials.gov/study/NCT05882877) | Interventional, multicenter, double-blind study | 3 | Recruiting |  |  |  |  |
| Rocatinlimab | OX40 | Subcutaneous | Adolescent participants with moderate-to-severe AD. | [NCT05704738](https://www.clinicaltrials.gov/study/NCT05704738) | Interventional, randomized, placebo-controlled, double-blind study | 3 | Active, not recruiting |  |  |  |  |
| YH35324 | Long-acting IgETrap-Fc fusion protein | Subcutaneous | Healthy subjects or subjects with allergic diseases, who have atopy. | [NCT05564221](https://www.clinicaltrials.gov/study/NCT05564221?cond=NCT05564221&rank=1) | Randomized, double-blind, placebo/active-controlled, multiple ascending dose study | 1 | Active, not recruiting |  |  |  |  |
| **Chronic spontaneous urticaria** | | | | | | | |  |  |  |  |
| Dupilumab | IL-4Rα | Subcutaneous | Children aged between 2 and 12 years with uncontrolled disease despite regular H1-AH treatment. | [NCT05526521](https://www.clinicaltrials.gov/study/NCT05526521) | Single-arm, multi-center study | 3 | Recruiting |  |  |  |  |
| YH35324 | IgE | Subcutaneous | Male or female adults aged ≥ 19 to ≤ 55 years.  Serum total IgE level ≥ 30 IU/mL or 30 to 700 IU/mL or >700IU/mL.  Healthy subjects, subjects with a history of mild allergic diseases (including CS), and individuals with moderate to severe atopic dermatitis. | [NCT05564221](https://www.clinicaltrials.gov/study/NCT05564221) | Randomized, double-blind, placebo/active-controlled, multiple ascending dose | 1b | Active, not recruiting |  |  |  |  |
| UB-221 | IgE | Intravenous | Adults with CSU lasting for more than six weeks. | [NCT04175704](https://www.clinicaltrials.gov/study/NCT04175704?cond=UB-221&rank=3) | Randomized, single-blind, placebo-controlled, parallel-group, single ascending dose study | 1 | Not yet recruiting |  |  |  |  |
| CMAB007 | IgE | Subcutaneous | 15 to 75 years old with CSU at least for 6 months being asymptomatic despite the use of H1-AH treatment. | [NCT06365879](https://www.clinicaltrials.gov/study/NCT06365879) | Randomized, double-blind, positive parallel controlled, multicenter study | 3 | Recruiting |  |  |  |  |
| **Non-esophageal eosinophilic gastrointestinal disorders** | | | | | | | | |  |  | **Non-esophageal eosinophilic gastrointestinal disorders** |
| Dupilumab | IL-4Rα | Subcutaneous | Aged 12-70 years with histologically active EoG with a peak gastric count of ≥ 30 eos/hpf in at least 5 hpfs in the gastric antrum and/or body, with moderate-to-severe disease symptoms, and stable medical management of EoG. | [NCT03678545](https://www.clinicaltrials.gov/study/NCT03678545?cond=NCT03678545&rank=1) | Multi-center, randomized, double-blind, placebo-controlled trial | 2 | Active, not recruiting |  |  |  |  |
| Dupilumab | IL-4Rα | Subcutaneous | Documented endoscopic biopsy supporting a pathologic diagnosis of EoG, with a history of ≥2 episodes of EoG (with or without EoD) symptoms per week in 8 weeks before screening, with moderate-to-severe severity of disease. | [NCT05831176](https://www.clinicaltrials.gov/study/NCT05831176?cond=NCT05831176&rank=1) | Randomized, double-blind,placebo-controlled, 3-part study | 2/3 | Recruiting |  |  |  |  |
| Cendakimab | anti-IL-13Rα1 and α2 | Not specified | Aged between 12 and 75 years with histologically-proved EoGE while on stable background therapy for EoGE with weekly symptom scores of ≥4/15 evaluated with the Izumo Scale. | [NCT05214768](https://clinicaltrials.gov/study/NCT05214768) | Multicenter, randomized, double-blind, placebo-controlled trial | 3 | Active, not recruiting |  |  |  |  |
| **Chronic rhinosinusitis with nasal polyps** | | | | | | | |  |  |  |  |
| Dupilumab vs Omalizumab | IL-4Rα | Subcutaneous | Severe, bilateral CRSwNP, symptoms of nasal congestion and loss of smell, and coexisting asthma. | [NCT04998604](http://clinicaltrials.gov/show/NCT04998604) | Multicenter, randomized, double-blind, active-controlled trial | 4 | Active, not recruiting |  |  |  |  |
| TQC2731 | anti-TSLP | Injection, not specified | Adults aged between 18-75 years with severe, bilateral CRSwNP who received nasal polyp surgery or received SCS treatment in the last 2 years. | [NCT06451640](https://www.clinicaltrials.gov/study/NCT06451640?cond=NCT06451640&rank=1)  [NCT06036927](https://clinicaltrials.gov/study/NCT06036927) | Multicenter, randomized, double-blind, placebo-controlled | 2 | Recruiting |  |  |  |  |
| Calpurbatug ( TRL1068) | Pathogen-protecting biofilm | Intravenous | Adults aged 18-85 with CRSwNP with a history of acute exacerbation with increased sinonasal discharge OR acute exacerbation post-FESS with increased sinonasal discharge AND sinonasal culture positive for *S.aureus* or *P.aeruginosa* without concomitant fungal infection in culture or PCR. | [NCT05355207](https://www.clinicaltrials.gov/study/NCT05355207?cond=NCT05355207%20&rank=1) | Interventional study | 1 | Not yet recruiting |  |  |  |  |
| Tezepelumab | anti-TSLP | Subcutaneous | Severe CRSwNP for at least 12 months with any standard of care treatment provided the participant is stable on that treatment for 30 days prior to screening with a history of SCS within the last 1 year and/or any history of sinonasal surgery. | [NCT04851964](https://www.clinicaltrials.gov/study/NCT04851964?cond=NCT04851964%20&rank=1) | Multicentre, randomized, double-blind, placebo-controlled, parallel-group study | 3 | Active, not recruiting |  |  |  |  |
| TQH2722 | IL-4Rα | Injection, not specified | Adults aged 18-75 years with bilateral chronic sinusitis (with or without nasal polyps) with a history of SCS within 2 years before the screening and/or sinus surgery in the prior 6 months. | [NCT06439381](https://www.clinicaltrials.gov/study/NCT06439381?cond=NCT06439381&rank=1)  [NCT06089278](https://www.clinicaltrials.gov/study/NCT06089278?cond=NCT06089278&rank=1) | Multicenter, randomized, continuing trial | 2 | Recruiting |  |  |  |  |
| Telikibart (GR1802) | IL-4Rα | Subcutaneous | Adults who participated and completed the NCT05873803 trial. | [NCT06015243](https://clinicaltrials.gov/study/NCT06015243?cond=GR-1802&rank=4) | Open-label, single-arm, multicenter study | 2 | Not yet recruiting |  |  |  |  |
| Telikibart (GR1802) | IL-4Rα | Subcutaneous | Adults aged 18-75 with bilateral, severe CRSwNP with prior treatment with SCS, and/or contraindicate to or intolerance to SCS, and/or with prior surgery to nasal polyps. | [NCT05873803](https://clinicaltrials.gov/study/NCT05873803?cond=GR-1802&rank=2) | Randomized, double-blind, placebo-controlled, multicenter study | 2 | Active, not recruiting |  |  |  |  |
| Telikibart (GR1802) | IL-4Rα | Subcutaneous | Adults aged 18-75 with bilateral, severe CRSwNP, treated with SCS within the last two years, and/or those who had undergone surgery for nasal polyps, with moderate-to-severe symptom severity. | [NCT06516302](https://clinicaltrials.gov/study/NCT06516302?cond=GR-1802&rank=3) | Randomized, double-blind, placebo-controlled, multicenter study | 3 | Not yet recruiting |  |  |  |  |
| Mepolizumab | IL-5 | Subcutaneous | Adults with bilateral CRSwNP eligible for biologics with a history of sinonasal surgery and three additional criteria including evidence of type 2 inflammation, need/contraindication to SCS, significantly impaired QoL, and severe loss of smell. | [NCT05923047](https://www.clinicaltrials.gov/study/NCT05923047?cond=NCT05923047&rank=1) | Randomized, controlled multicenter trial | 4 | Not yet recruiting |  |  |  |  |
| Mepolizumab | IL-5 | Subcutaneous | CRSwNP patients eligible for biologics suffering or not from comorbid severe asthma. | [NCT06069310](https://www.clinicaltrials.gov/study/NCT06069310?cond=NCT06069310&rank=1) | Observational study | NA | Recruiting |  |  |  |  |
| Mepolizumab | IL-5 | Subcutaneous | Adults with a doctor's diagnosis of bilateral, severe CRSwNP with a history of one FESS in general anesthesia performed before inclusion (no time limitation), no course of systemic steroids within the last 3 months, and confirmation of type-2 inflammation. | [NCT05598814](https://www.clinicaltrials.gov/study/NCT05598814?cond=NCT05598814&rank=1) | Randomized, two-arm trial | 4 | Recruiting |  |  |  |  |
| **Allergic rhinitis** | | | | | | | |  |  |  |  |
| Tezepelumab | TSLP | Subcutaneous | Patients with severe asthma and coexisting AR. | [NCT06189742](https://clinicaltrials.gov/study/NCT06189742) | Non-randomized, single-group assignment, open-label study | 2 | Active, recruiting |  |  |  |  |
| Stapokibart (CM310) | IL-4Rα | Subcutaneous | Adults aged 18-85 with SAR. | [NCT06171074](https://clinicaltrials.gov/study/NCT06171074?cond=CM310&rank=8) | Single-arm, multicenter study | 2 | Not yet recruiting |  |  |  |  |
| Stapokibart (CM310) | IL-4Rα | Subcutaneous | Adults aged 18-65 with uncontrolled SAR. | [NCT06300203](https://clinicaltrials.gov/study/NCT06300203?cond=CM310&rank=26) | Randomized, double-blind, placebo-controlled study | 2 | Not yet recruiting |  |  |  |  |
| Stapokibart (CM310) | IL-4Rα | Subcutaneous | Patients aged 12-65 with SAR. | [NCT06525597](https://clinicaltrials.gov/study/NCT06525597?cond=stapokibart&rank=1) | Randomized, double-blind, placebo-controlled study | 2 | Recruiting |  |  |  |  |
| Telikibart (GR1802) | IL-4Rα | Injection, not specified | Adults aged 18-75 with poor control of SAR with nasal CS. | [NCT06315426](https://clinicaltrials.gov/study/NCT06315426?cond=NCT06315426&rank=1) | Randomized, double-blind, placebo-controlled, multicenter study | 2 | Not yet recruiting |  |  |  |  |
| Telikibart (GR1802) | IL-4Rα | Subcutaneous | Adults aged 18-75 with uncontrolled SAR under conventional treatment. | [NCT06028490](https://clinicaltrials.gov/study/NCT06028490?cond=NCT06028490&rank=1) | Randomized, double-blind, placebo-controlled study | 2 | Recruiting |  |  |  |  |
